# RCytoscape: tools for exploratory network analysis

**DOI:** 10.1186/1471-2105-14-217

**Published:** 2013-07-09

**Authors:** Paul T Shannon, Mark Grimes, Burak Kutlu, Jan J Bot, David J Galas

**Affiliations:** 1 Fred Hutchison Cancer Research Institute, Seattle Washington, and the Institute for Systems Biology, 401 Terry Ave. N, Seattle, WA, USA; 2 Division of Biological Sciences, Center for Structural and Functional Neuroscience, University of Montana, Missoula, MT, USA; 3 Institute for Systems Biology, 401 Terry Ave. N, Seattle, WA, USA; 4 Delft University of Technology, Delft Bioinformatics Lab, Delft, The Netherlands; 5 Pacific Northwest Diabetes Research Institute, 720 Broadway, Seattle, WA 98120, USA

**Keywords:** Biological networks, Visualization, Exploratory data analysis, Statistical programming, Bioinformatics

## Abstract

**Background:**

Biomolecular pathways and networks are dynamic and complex, and the perturbations to them which cause disease are often multiple, heterogeneous and contingent. Pathway and network visualizations, rendered on a computer or published on paper, however, tend to be static, lacking in detail, and ill-equipped to explore the variety and quantities of data available today, and the complex causes we seek to understand.

**Results:**

RCytoscape integrates R (an open-ended programming environment rich in statistical power and data-handling facilities) and Cytoscape (powerful network visualization and analysis software). RCytoscape extends Cytoscape's functionality beyond what is possible with the Cytoscape graphical user interface. To illustrate the power of RCytoscape, a portion of the Glioblastoma multiforme (GBM) data set from the Cancer Genome Atlas (TCGA) is examined. Network visualization reveals previously unreported patterns in the data suggesting heterogeneous signaling mechanisms active in GBM Proneural tumors, with possible clinical relevance.

**Conclusions:**

Progress in bioinformatics and computational biology depends upon exploratory and confirmatory data analysis, upon inference, and upon modeling. These activities will eventually permit the prediction and control of complex biological systems. Network visualizations -- molecular maps -- created from an open-ended programming environment rich in statistical power and data-handling facilities, such as RCytoscape, will play an essential role in this progression.

## Background

Molecular biology has made great progress in recent years by measuring the abundance and characteristics of many kinds of molecules, often at a global level. Whole genomes have been sequenced, global mRNA and miRNA levels assessed, protein expression measured, phosphorylation and methylation states assayed. Many protein structures have been determined. Progress towards understanding the dynamic relations and interactions among these molecular components, however, has lagged significantly [1]. It is precisely these complex system behaviors which must be understood in order to comprehensively predict and control cellular processes in health and disease.

Causal explanations in molecular biology of sufficient depth and completeness to explain disease, and to create the basis for successful therapy, are almost never simple. Cancer, for example, is currently understood to consist of six separate processes, or “hallmarks”, each of which is controlled by redundant and overlapping pathways [2]. Even classic single gene disorders show variable age of onset and severity, apparently due to the influence of modifier genes [3]. A recent theoretical framework establishes that the control of gene regulatory networks requires prior control of more than half the constituent nodes [4]. Phosphorylation networks exhibit similar complexity and resistance to manipulation [5]. As we explore and map this complex terrain, using ever larger amounts of heterogeneous (and often noisy) data, network visualization tools integrated within a statistically powerful programming environment will prove indispensable. RCytoscape provides one such set of tools.

Many and diverse kinds of software will be needed in order to achieve prediction and control of cellular processes. We distinguish two broad classes on the basis of novelty. Software for routine bioinformatics, in which well-studied algorithms are applied to well-understood kinds of data, can be distinguished from software required for novel bioinformatics and computational biology, in which the data are often less well understood, and for which new algorithms must be developed. Routine bioinformatics is often accomplished with web-based and point-and-click desktop applications. Gene Set Enrichment Analysis (GSEA), a Java application offered by the Broad Institute calculates over-representation in curated gene data sets for an experimenter’s expression data: one loads mRNA expression data from a file, chooses the gene set categories of interest, obtains a list of enriched categories. No opportunity is provided to filter the input data, to transform it in possibly revealing ways, to correlate with related data, to display in the context of known gene and protein interactions, to apply experimental algorithms before and after the enrichment step. Novel bioinformatics and computational biology, however, require a programming language (or languages). They depend upon robust and full-featured statistical and modeling libraries, easy access to many kinds of data and annotation, and strong visualization capabilities, harnessed together into a programming environment for exploration, modeling and analysis.

In a recent review of network display software incorporating high throughput molecular biology data [6] Gehlenborg et al. conclude that “truly integrated visualization of systems biology data across the entire range of possible data types is still very much in its infancy.” With one notable exception, the software included in this review are point-and-click web or desktop applications. (The exception is a small set of multivariate gene expression analyses and visualizations accomplished with R, whose special capabilities and utility we discuss below). The effective integration and visualization of large quantities of multiple kinds of data requires frequent recourse to statistical programming and exploratory visualization. Normalization techniques, for example, must be chosen and applied with caution, iteratively and provisionally, with frequent recourse to visual assessment when data from diverse sources are combined. For the foreseeable future these integration and visualization activities will not be routine, will require programming and interactive engagement with the data, and will thus be beyond the capabilities of point-and-click software performing routinized analyses.

In addition to the above classification of software by novelty, we also distinguish, on another axis, different types of bioinformatic and computational biology activities. Drawing in part upon an NIH classification [7] these categories include: exploratory data analysis, confirmatory data analysis (including hypothesis testing), statistical inference (including clustering and classification), mathematical modeling, and simulation. Listed in an ascending order of complexity and rigor, and sorted into what might be early, medium and late stage bioinformatic or computational research activities, in fact these activities are interleaved and repeated, in an improvised manner, throughout such research projects.

Exploratory Data Analysis (EDA) [8] and information graphics [9] are two related disciplines associated, respectively, with statistician John Tukey and data visualization pioneer Edward Tufte. Together they provide the rationale for high quality network visualization, and describe the role it can play in novel bioinformatics and computational biology. Their combined claim may be summarized thus: that the judicious display and exploration of data contributes insight into the data and into possible causal relationships which may otherwise be missed. John Tukey: “[Visualization methods] … are there, not as a technique, but rather as recognition that the picture-examining eye is the best finder we have of the wholly unanticipated” [10]. As a proponent of good techniques for visualizations, Tufte has said, “There is no such thing as information overload, just bad design. If something is cluttered and/or confusing, fix your design” [11]. Integrating a powerful scripting language with network visualization software, as we argue for here, empowers Tukey’s picture-examining eye, and makes possible Tufte’s injunction to “fix your [visual] design”.

Thus the goal is to improve network visualization: to increase the ease and sophistication with which detailed molecular maps can be constructed, in order that they may contribute to all of the novel bioinformatic and computational biology, network-related activities listed above.

### Software

Cytoscape, which we first released in 2002 [12], has become the standard open source network visualization software used in molecular biology [13]. Over the years it has been refined, extended, and has attracted a large number of users and developers. The core data type is a network (a mathematical graph, or multigraph) having nodes and edges, and accompanied by any number of data attributes on those nodes and edges. Cytoscapes’s “vizmapper” translates node and edge data attributes into visible attributes (from gene expression to node color, for instance). A plugin architecture allows for extensions to the core code. Over one hundred plugins are available, providing access to many bioinformatic resources and analyses.

Cytoscape, despite its many strengths, has not been well-suited to novel bioinformatics and computational biology, because it has lacked a full-featured, bioinformatically-capable scripting language. Three are three candidate open-source scripting languages in common use today: Perl, Python, and R. Cytoscape’s ScriptingPluginEngine only supports languages implemented to run in the Java Virtual Machine, thus ruling out Perl, R and anything but a limited version of Python (“Jython”). Ruby, JavaScript, Groovy, Clojure, and the aforementioned Jython are supported, but these languages are little used in and somewhat limited for bioinformatics. Jython, for instance, is unable to run *NumPy*, “the fundamental package for scientific computing with Python” [14] or to call out to other compiled code.

Lacking a full-featured, bioinformatically-capable scripting language, the most effective way to extend the capabilities of Cytoscape beyond those offered by the Cytoscape core development team, and by Cytoscape plugin writers, has been to write another plugin. Unfortunately, this is a task for a seasoned Java software developer, and largely impractical and/or inefficient for research bioinformaticians and computational biologists, for two reasons. First, Java programming is complex and time-consuming: object inheritance must be understood, Java classes designed and implemented, the Cytoscape API grasped, the code compiled, classpaths resolved, and jar files dynamically loaded. Second, the new plugin will be subject to the same constraints and lack of flexibility found in all plugins and in Cytoscape itself: only pre-conceived operations which can be accomplished via a point-and-click interface are permitted, with parameterization limited to the provided GUI, and with the reproducibility of any analysis reduced to recording and repeating a precise series of mouse clicks. Cytoscape, in the absence of a strong bioinformatics scripting language, can be useful in routine bioinformatics, but is not well-suited for the practice of novel bioinformatics and computational biology.

With the appearance of the CytoscapeRPC plugin in 2011 [15] full scripting finally became possible. CytoscapeRPC employs XMLRPC [16], a well-known HTTP-based inter-process communication protocol which is supported by the three top scripting language candidates, Perl, Python and R. For the first time, most of Cytoscape’s point-and-click commands, and many other internal operations, could be accomplished by function calls from a scripting language, running in another process. Data and networks residing in Cytoscape can in addition, be transmitted to the environment of the scripting language. Thus augmented, Cytoscape becomes well-suited to novel bioinformatics and computational biology. The first version of RCytoscape, using CytoscapeRPC, appeared in the fall of 2011.

To make these capabilities convenient to use, and to insulate RCytoscape’s programmer interface from changes to Cytoscape API (about which more below, in the discussion of the recent release of Cytoscape 3.0) one task remained: to create an R programing interface to Cytoscape so that the details of the XMLRPC protocol are hidden, and so that Cytoscape commands appears as “natural” operations in the scripting language. Thus, instead of

RCytoscape scripters can write

We chose R from among the three candidate languages. Perl was rejected because it is not interactive, and because its popularity within bioinformatics appears to have declined in recent years, perhaps due a perceived deficiency in statistical and modeling domains, for which bioinformaticians and computational biologists usually turn to Python, R, or a compiled language such as C or C++.

Python and R are both strong candidates for scripting Cytoscape. Both work in interactive and ‘batch’ modes, and both are used in a wide variety of scientific and engineering domains. They each support an interface to compiled languages (C, C++, Fortran) providing access to extra speed or special libraries as needed.

We selected R over Python. As the open source implementation of the S programming language, R has been shaped by more than 30 years of use in statistics, data mining, and numerical modeling. Though R has neither the syntactic elegance of Python, nor the dynamic power of Ruby, its long history, and its broad adoption as the standard open source “software environment for statistical computing and graphics” [17] means that it offers unparalleled convenience and power for analyzing data. Python, while generally acknowledged to be the better-designed language, has always lagged R in features needed by those doing serious statistics, data-mining, and other data- and mathematically-oriented computing. If mailing list volume is a reliable indicator, the R/Bioconductor bioinformatics community is substantially more active than Biopython [18]. Among data-mining and statistical programming languages, R in recent years has become the most popular [19].

Many basic and sophisticated statistical functions are built into the language itself (‘base R’); these often embody the state of the art in these algorithms. Their easy availability in the language has for many years attracted those wanting to create new capabilities and analyses. This positive feedback loop continues to this day: existing mathematical and visualization capabilities attract additional capabilities because those new capabilities are easier to create in R than in other languages. For those working in the field of network inference, in statistically robust analysis of high throughput and next-generation sequencing data, R’s mathematical and visualization strengths confer a substantial advantage. An integration of Python and Cytoscape will be useful, welcome and popular. However, the statistical, data-mining, visualization and bioinformatic strengths and popularity of R, and the Bioconductor Project, led us to choose R as our scripting language, and to create a new Bioconductor R package, “RCytoscape” [20].

Another strength of R over Python - if only by a matter of degree - is software support for, and wide adoption of the practice of - “reproducible research” [21]. R has evolved an integrated set of coding and data distribution practices, embodied in the standard R “package” structure, expressly designed to support reproducible research The R package is a standardized collection of directories and files, including R source code; C, C++ and Fortran source code when applicable; documentation files with executable demonstration code snippets; any required data; unit tests to establish the reliability of the code; and not least, a “vignette”. The latter is a text document which implements Donald Knuth’s “literate programming” [22]: R code is interspersed with narrative text which explains the logic and details of the analysis undertaken. Whenever the package is built or tested, the code is run, and a pdf version of the vignette is created. A well-written vignette and its package thus convey everything an interested reader needs in order to reproduce an analysis. It thus sets the stage for reliably disseminating data, software, and results. We include such a package and vignette to complement the necessarily less detailed treatment of glioblastoma proneural tumors presented in the Discussion section below.

As Gehlenborg et al. [2] emphasize, the assimilation and integration of diverse data types into network analysis and visualization is an urgent task. With new types of molecular measurement data appearing frequently -- as seen in a large public release from the ENCODE project [23] in 2012 -- an optimal environment will have easy access to the data and tools for analyzing it. The open-source R-based Bioconductor [24] project, along with BioPython and BioPerl, have a long history of providing routine access to such data and tools.

In the case study we present below, our focus will be upon data visualization in the context of exploratory data analysis to demonstrate the cartographic capabilities of RCytoscape. Heterogeneous experimental data is marshaled and filtered in R, molecular pathways assembled, a sequence of interactive maps displayed in Cytoscape, leading to the identification of molecular interaction patterns missed by the clustering analysis upon which the case study builds.

## Implementation

RCytoscape connects R and Cytoscape using XMLRPC, a standard WWW protocol for transmitting messages and data between programs. It is implemented in the ‘CytoscapeRPC’ plugin and by the complementary open source RCytoscape package, written in R and freely available through the Bioconductor web site. Nearly all of Cytoscape’s operations appear as function calls in R. Networks can be assembled from data marshaled in R from public or proprietary network databases and then displayed in Cytoscape. Conversely, data and networks loaded into Cytoscape using native Cytoscape methods may be imported into an R session via a simple function call. Network layout and visual mapping for nodes and edges can be specified and applied. Zooming and selection, filtering based on attributes, animation (changing node positions, changing node and edge colors, shapes and sizes) all become possible. R is a complete, interpreted and batch-oriented programming environment, so network creation, manipulation, exploration and analysis can proceed one command at a time, or be combined into scripts and programs, encouraging reproducible analysis, and thus reproducible research. By contrast, recreating visualizations in Cytoscape, and any analyses performed through the traditional Cytoscape combination of interactive commands and plugins, can only be accomplished by recapitulating a precise sequence of mouse clicks, which is error-prone and which restricts the dissemination of useful analyses.

Another virtue of scripting control of Cytoscape is the ease which with animations (dynamic displays) can be made. When a sequence of Cytoscape maps are displayed, or when a single map is modified by scripted commands, an extra dimension of information is added. This extra dimension may be time, based upon time-series experimental data, but it can equally well, for example, be cell-type, or treatment. The underlying scripting and display techniques are the same: several frames are shown in sequence, and optionally saved as replayable images (see Additional files 1, 2 and 3).

RCytoscape provides three methods for distributing (‘publishing’) biomolecular maps, static or dynamic, each tailored to a different audience. Simple browser publishing is supported by exporting dynamic network images from RCytoscape into a web page. For experienced Cytoscape users, networks created with RCytoscape may be saved and shared as Cytoscape session files. In laboratory settings where some programming skill is available, and particularly where cross-laboratory collaborations are involved, code and data can be combined into easily shared R packages so that networks, experimental data, algorithms and visualization become ‘live documents’ shared and evolving over time (the case study, below, is provided as an R package; Additional files 4 and 5 demonstrate this valuable scientific practice).

With the release of Cytoscape 3.0 in February of 2013, a new application architecture is available. With version 3.1, expected in late 2013, built-in support for a “RESTful” [25] inter-process communication protocol is planned, coupled to the new “TaskFactory” API. This will provide the basis for the next version of RCytoscape. A native Python scripting interface will very likely appear then as well. However, even with the availability of native Python scripting for Cytoscape, R and RCytoscape will continue to offer unique capabilities, power and convenience due to R’s long history, and its current capabilities and popularity for doing bioinformatics and computational biology.

## Results and discussion

We demonstrate the synergistic capabilities of RCytoscape with a small biological case study. (An extended version of this case study will be found in Additional file 6). Verhaak et al. (2010) used consensus average linkage clustering of gene expression and genomic abnormalities to distinguish four subclasses of Glioblastoma multiforme (GBM) tumors. Interestingly, these data-derived subclasses correspond to the neural lineage of the tumor cells and, to a limited extent, to differing treatment strategies. We restrict our attention to the least treatable of these four subclass: fifty-five tumors in the “Proneural” class. From these we select thirteen which best match the signature proposed by Verhaak et al. for this subclass: high expression and amplification of PDGFRA. We reason that if the pathway neighbors of PDGFRA display heterogeneity in mRNA expression, copy number and mutations, despite PDGFRA consistency, that this heterogeneity may have implications for tumor treatment. Combined visual and simple statistical exploratory analysis does indeed reveal such heterogeneity.

Complete code and data to reproduce the analysis described here is provided in the supplemental R package "ProneuralHeterogeneity" (Additional file 4). For narrative clarity and to economize on space, we present a typical RCytoscape session in “pseudocode”, leaving out the many fine-grained coding details. In many cases, the pseudocode show here differs little from calls to functions provided in the supplemental ProneuralHeterogeneity package.

### Data retrieval

We use two R packages to retrieve selected KEGG pathways and to obtain high throughput assay data from TCGA. These packages deliver R data objects to the RCytoscape session via a few function calls, returning standard data objects which are ready for further computation. These simple steps contrast with traditional bioinformatic data retrieval, usually accomplished via web browsing and ftp, and followed by reformatting of the downloaded data before it is in a computable form.

We retrieved and combined three pathways from the many curated by KEGG: “Pathways in Cancer”, “Cell Cycle” and “Glioma”, eliminating redundant reactions, and creating a preliminary single network of molecular interactions with which to illuminate the TCGA data. This combined network consists primarily of signaling and regulatory relationships, and provides good coverage of the Proneural tumor signature gene (PDGFRA) and the signaling pathways in which it is involved.

Use the CGDSR package to etrieve expression and genomic variation data for the genes in the combined KEGG pathway.

These three TCGA data tables created above contain one row for each geneID, and one column for each tumor sample in the TCGA repository. We are interested here only in those categorized as "Proneural" and more specifically, only those which match or exceed the signature described in Verhaak et al.: log fold change mRNA expression of the receptor tyrosine kinase PDGFRA greater than 2.0 (compared to reference) and PDGFRA copy number classified as “amplified”. We call these signature-consistent tumors "strong Proneural", and created a function to identify them whose details are elided here:

Visualization begins by examining three TCGA-derived assays (expression, mutations, copy number) of each of the thirteen strong proneural tumors, projecting that data onto the Cytoscape display of the combined KEGG network. Since the network is large, individual details may be missed, but the broad characteristics, and heterogeneity of the tumors is easily seen. “Visual mapping” rules specify how node color, size and shape are to reflect the assay data. Each tumor is visualized in turn, and the result (an animated image file, “twoTumorsPDGFRAneighborhood.gif”) can be found in Additional file 1.

### Programmatic visual mapping

Visual mapping rules are traditionally specified in Cytoscape via a graphical user interface (called the “vizmapper”) but are arguably far easier to handle with R commands. We learn the distribution of the mRNA log-fold expression, first numerically (expressed as min, max, median, first and third quartile), then visually, with a histogram. We use this knowledge to parameterize node color mapping.

Similarly, the distribution of copy number variation is mapped to node border color and thickness, conveying deletion and amplification. Amino acid substitutions are added to node labels (“PDGFRA C235Y") for the tumors in which they appear. We use node shape to indicate molecule type (ligand, receptor kinase, transcription factor, etc.). Finally, we devise a vizmapping rule for node size which uses an informal estimate of overall tumorigenic signifcance of each gene so that, for instance, a highly expressed, amplified gene with a non-synonymous mutation is rendered as a node with a large diameter.

Network layout can be a complex problem. Automated algorithms, of which Cytoscape offers a useful collection, have many strengths. We find that these algorithms, though useful, can always be improved upon as we seek an optimal display for large and/or complex networks. RCytoscape encourages a combined layout strategy: an appropriate automated Cytoscape layout algorithm is applied in the early development stages of a project, and is then manually fine-tuned using direct placement of some nodes. The final layout is saved for reuse in the future via a single function call:

Any network displayed in Cytoscape can be saved as an image, and any succession of such displays can be assembled into an animation easily viewed in a web browser. Three such animations will be found in Additional file 1 (two tumors, PDGFRA neighborhood); Additional file 2 (thirteen tumors, large cancer-related network); Additional file 3 (thirteen tumors, PDGFRA neighborhood). Two representative images from the first animation (Additional file 1) are presented and discussed below.

To create the zoomed-in animation, select PDGFRA, and its first two network neighbors, then zoom in on this subnetwork before running the data animation:

### Receptor Tyrosine Kinases (RTKs)

**Figure 1:**
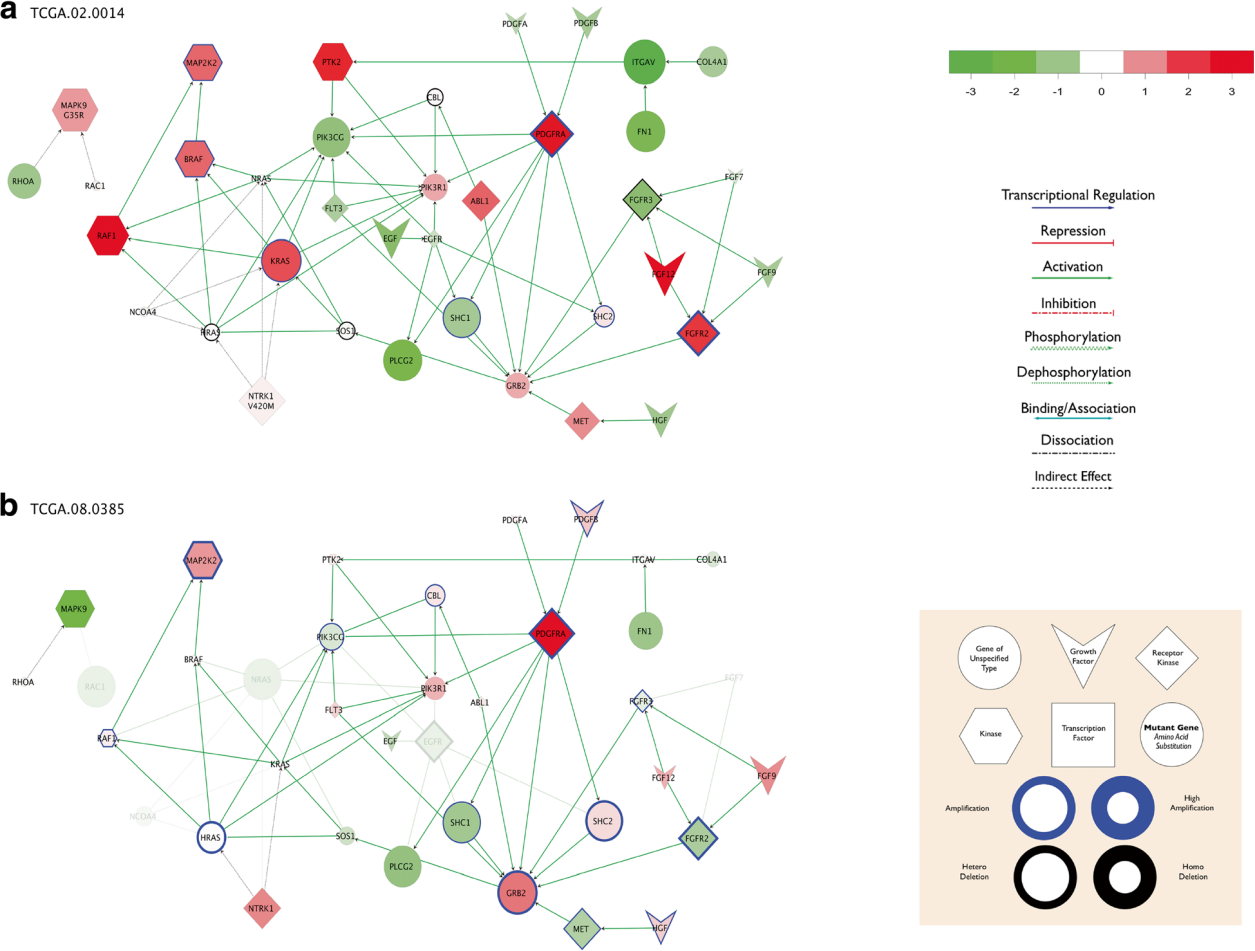
**A static display of (a) tumor TCGA.02.0014 and (b) TCGA.0.0835.** mRNA differential expression, copy number and mutation data mapped onto the same composite reference PDGFRA-neighborhood subnetwork (KEGG ‘Pathways in Cancer’, ‘Glioma’, ‘Cell Cycle’). **(c)** Key: Node color indicates differential expression (log-fold-change with respect to normal brain tissue). **(d)** Edges indicate molecular relationships as indicated. **(e)** Node shape indicates functional gene type; node border color and width indicates genomic copy number. Node size indicates possible tumorigenic importance, a function of mRNA expression, copy number and non-synonymous mutation. Non-aberrant genes and relationships are dimmed out.

The animations created above, as well as the two static images (Figure 1) selected from the second sequence, reveal heterogeneous expression and copy number in the immediate network vicinity of PDGFRA, the gene whose abnormalities define the Proneural subtype. PDGFRA is a receptor tyrosine kinase (RTK), a class of proteins which, when constituitively activated, contribute to unrestrained cell growth and tumorigenesis. A full discussion of RTKs and strong proneural tumor heterogeneity will be found in Additional file 6, but we here briefly note that although both tumors exhibit amplified and over-expressed PDGFRA, only tumor 385 shows the ligand PDGFB to be amplified and over-expressed as well, a phenomenon which suggests a possible autocrine loop, and a more important role for PDGRFA signaling than in tumor 014. Tumor 014, by contrast, shows an over-expressed and amplified RTK ligand/receptor pair (FGR12 and FGFR2) which is inactive in tumor 0365. This contrast, which is immediately visible in the RCytoscape-created map, may have implications for the selection of therapeutic targets in the treatment of glioblastoma Figure 1.

## Conclusions

Detailed, data-driven network visualization and the open-ended computational power and statistical richness of the R programming environment can be useful at all stages of analysis of biological data. RCytoscape enables the construction of detailed molecular maps that reveal significant heterogeneity beyond statistically robust classifier consensus average linkage hierarchical clustering obtained by Verhaak et al. [16]. Differences seen in the RCytoscape-derived maps suggest different autocrine loops may be active in proneural glioblastoma tumors, with two complimentary gene/protein pairs in each. Such a classification is valuable, and can set the stage for the careful characterization of individual tumors and design of new treatment strategies. Subsequent steps along the road to clinically useful prediction and control of these phenomena will involve more rounds of confirmatory and exploratory data analysis, simulation and modeling.

Linking R with Cytoscape has value for many different kinds of analyses beyond what is described here. Recently, Grimes et al., [26] used RCytoscape to graph nodes in two and three dimensions using coordinates derived from dimension reduction (embedding) algorithms, and plot networks using protein-protein interaction edges merged from three different databases, setting visual properties for nodes and edges based on quantitative information from a lung cancer phosphoproteomic data set. RCytoscape will be useful to anyone who wishes to add network visualization and analysis to the rich resources available in the R programming environment.

### Availability of supporting software and data

We include an R package “ProneuralHeterogeneity” in Additional file 4, which includes complete data, documentation, and unit-tested executable code with which to reproduce the two maps shown above. Code is also provided for the creation of more comprehensive networks, and for visualizing any number of the TCGA GBM tumors, along with an animation showing each of the thirteen “strong proneural” tumors in a loop for comparison and exploration.

## Availability and requirements

**Project name:** RCytoscape

**Project home page:**http://bioconductor.org/packages/release/bioc/html/RCytoscape.html

**Operating system(s):** Platform independent

**Programming language:** R

**Other requirements:** R >= 2.15, Cytoscape >= 2.8.0

**License:** GNU GPL-2

## Competing interests

There are no competing interests.

## Authors’ contributions

PS conceived, designed and wrote the software, and wrote the manuscript, MG contributed to the software design and the manuscript, BK contributed to the software design and the manuscript, JB advised in the software design and implementation, DG contributed to the manuscript. All authors read and approved the final manuscript.

## Supplementary Material

Additional file 1 (twoTumorsPDGFRAneighborhood.gif: an animated data display of the PDGFRA neighborhood of the two tumors discussed in the body of the article). Click here for file

Additional file 2 **(thirteenTumorsFullNetwork.gif: an animated data display of thirteen tumors (a superset of the two discussed in the paper and animated in Additional file **4**) in the context of a much larger collection of cancer-related networks).** Click here for file

Additional file 3 (thirteenTumorsPDGFRAneighborhood.gif. an animated data display of the "strong proneural" tumors, focused in upon the network neighborhood of PDGFRA). Click here for file

Additional file 4 (The ProneuralHeterogeneity R package, with annotated executable code and data with which to reproduce a superset of the analysis presented in the article). Click here for file

Additional file 5 (Proneural Heterogeneity vignette). Click here for file

Additional file 6 (An extended discussion of Receptor Tyrosine Kinase activation in heterogeneous Proneural GBM tumors (an abbreviated version appears in the body of the article). Click here for file
