# Supplementary figures and images for: RCytoscape: tools for exploratory network analysis
Source: BMC Bioinformatics. 2013 Jul 9;14:217. doi: 10.1186/1471-2105-14-217 (PMC3751905; doi:10.1186/1471-2105-14-217)

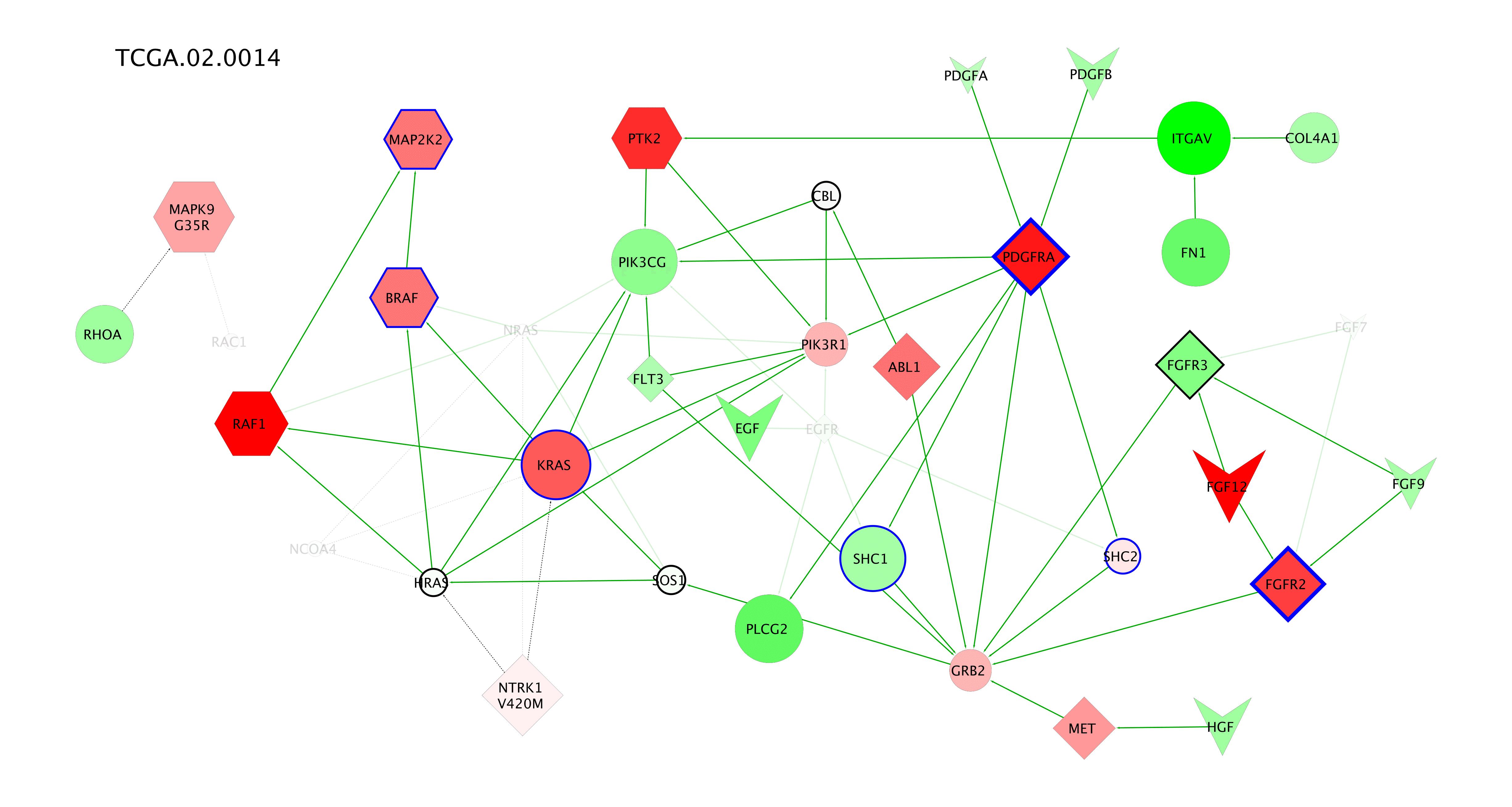

Supplement: Additional file 1 — (twoTumorsPDGFRAneighborhood.gif: an animated data display of the PDGFRA neighborhood of the two tumors discussed in the body of the article). [file 1471-2105-14-217-S1.gif]

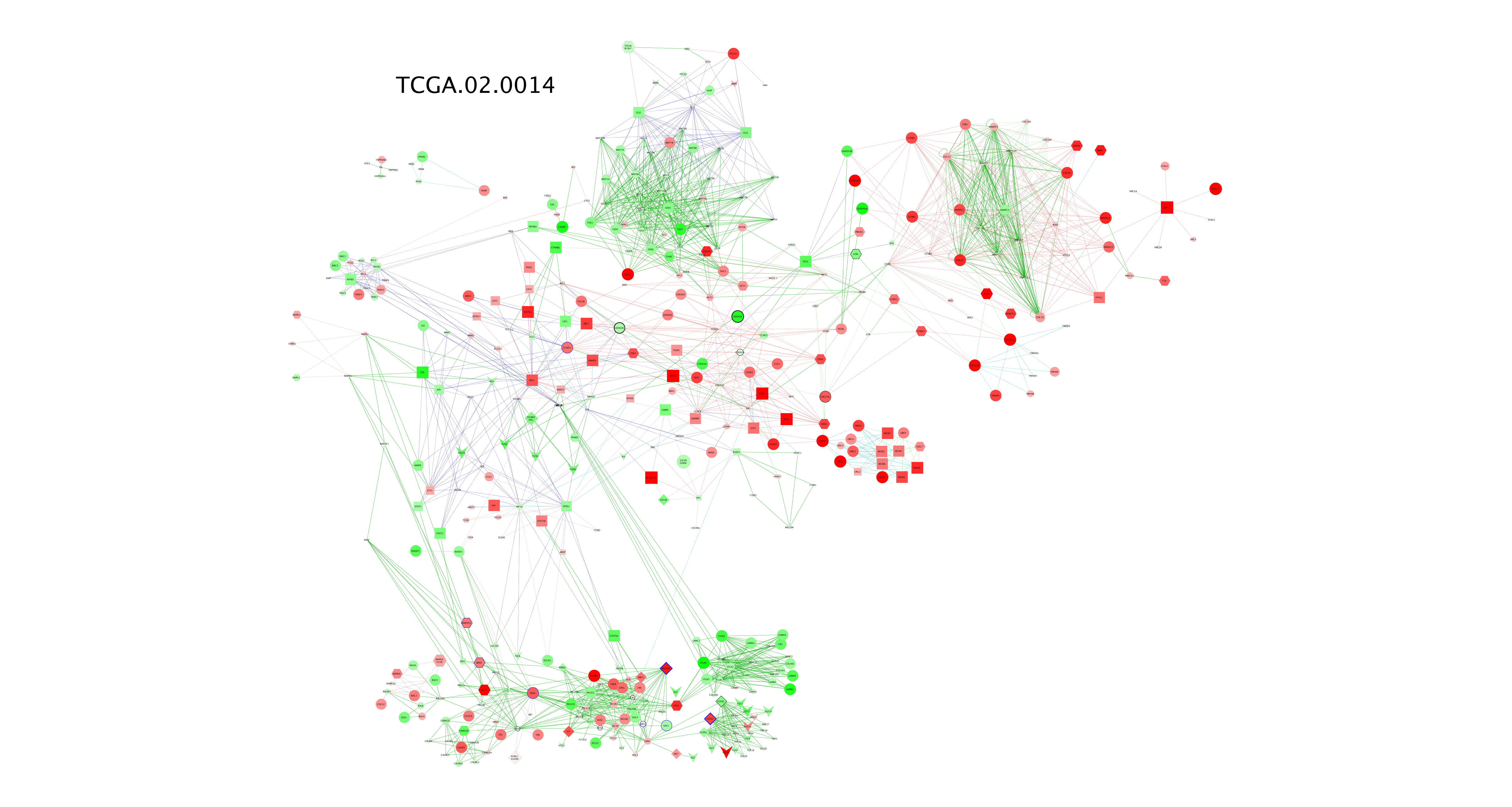

Supplement: Additional file 2 — (thirteenTumorsFullNetwork.gif: an animated data display of thirteen tumors (a superset of the two discussed in the paper and animated in Additional file 4) in the context of a much larger collection of cancer-related networks). [file 1471-2105-14-217-S2.gif]

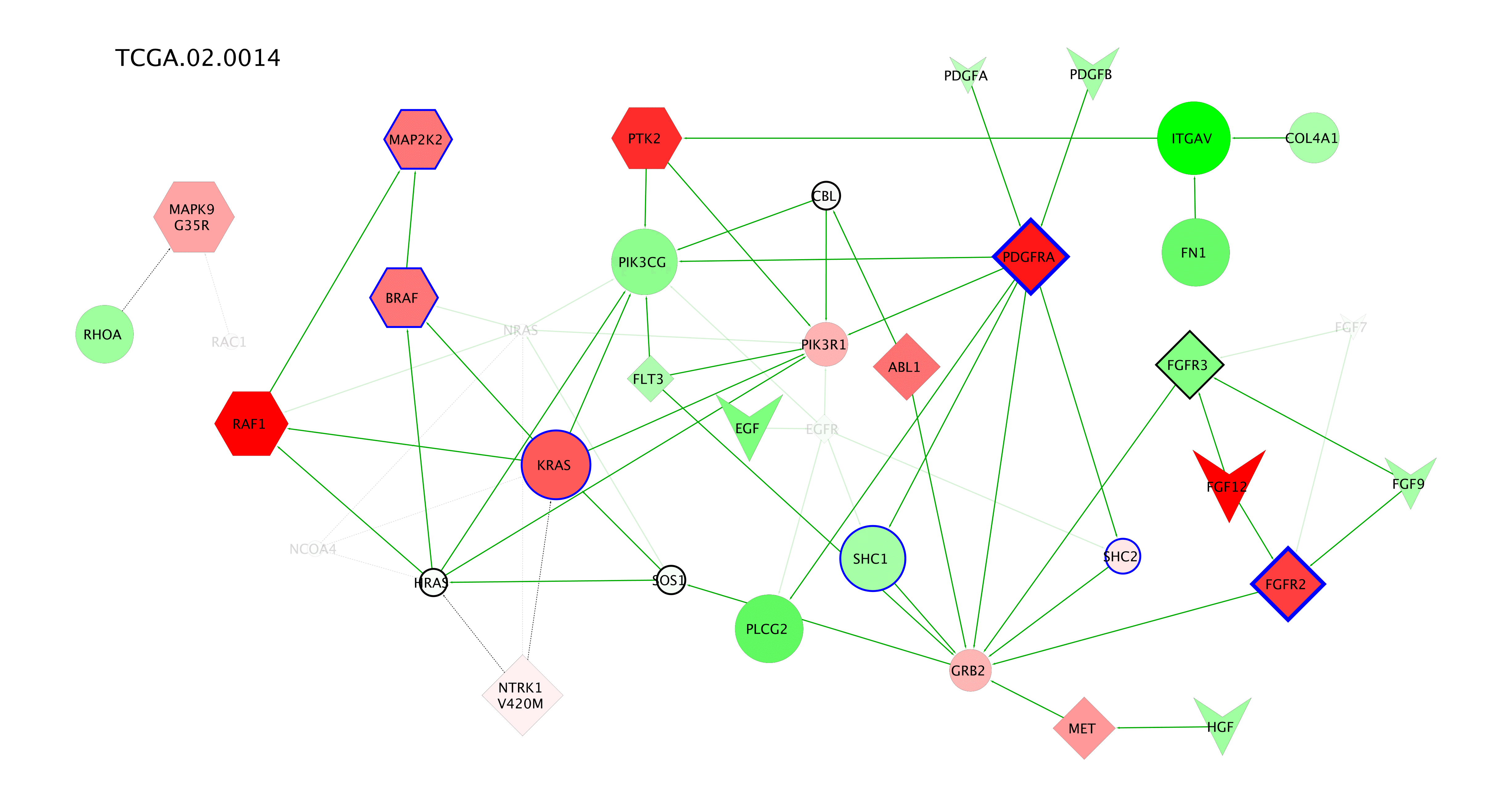

Supplement: Additional file 3 — (thirteenTumorsPDGFRAneighborhood.gif. an animated data display of the "strong proneural" tumors, focused in upon the network neighborhood of PDGFRA). [file 1471-2105-14-217-S3.gif]

# Strong Proneural Tumors Clustered on Gene Expression in Three KEGG Pathways

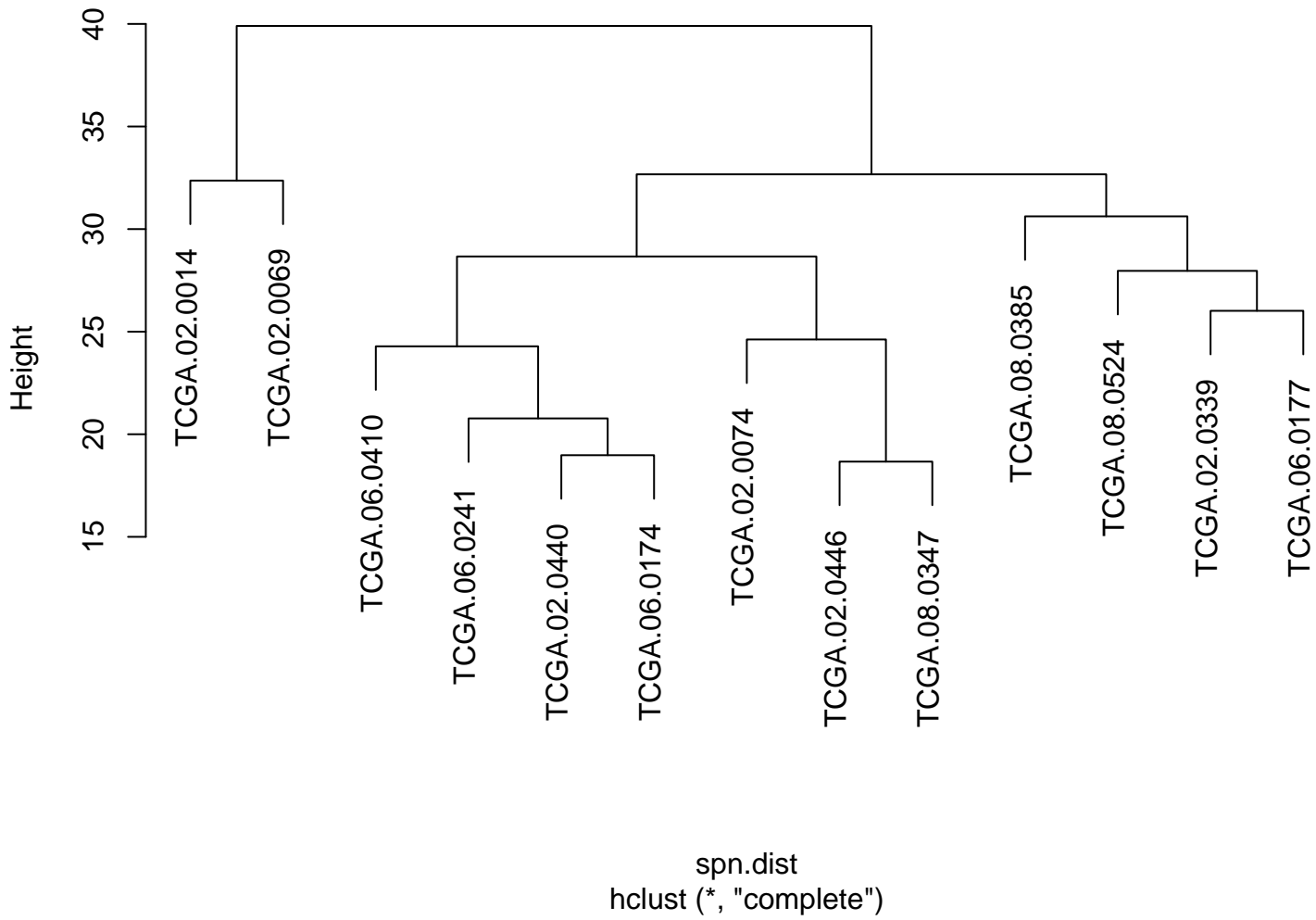

Supplement: Additional file 5 — (Proneural Heterogeneity vignette). [file 1471-2105-14-217-S5.gz › ProneuralHeterogeneity/inst/doc/ProneuralHeterogeneity-cluster-strong-proneurals.pdf]

## Z-score log fold mRNA expression

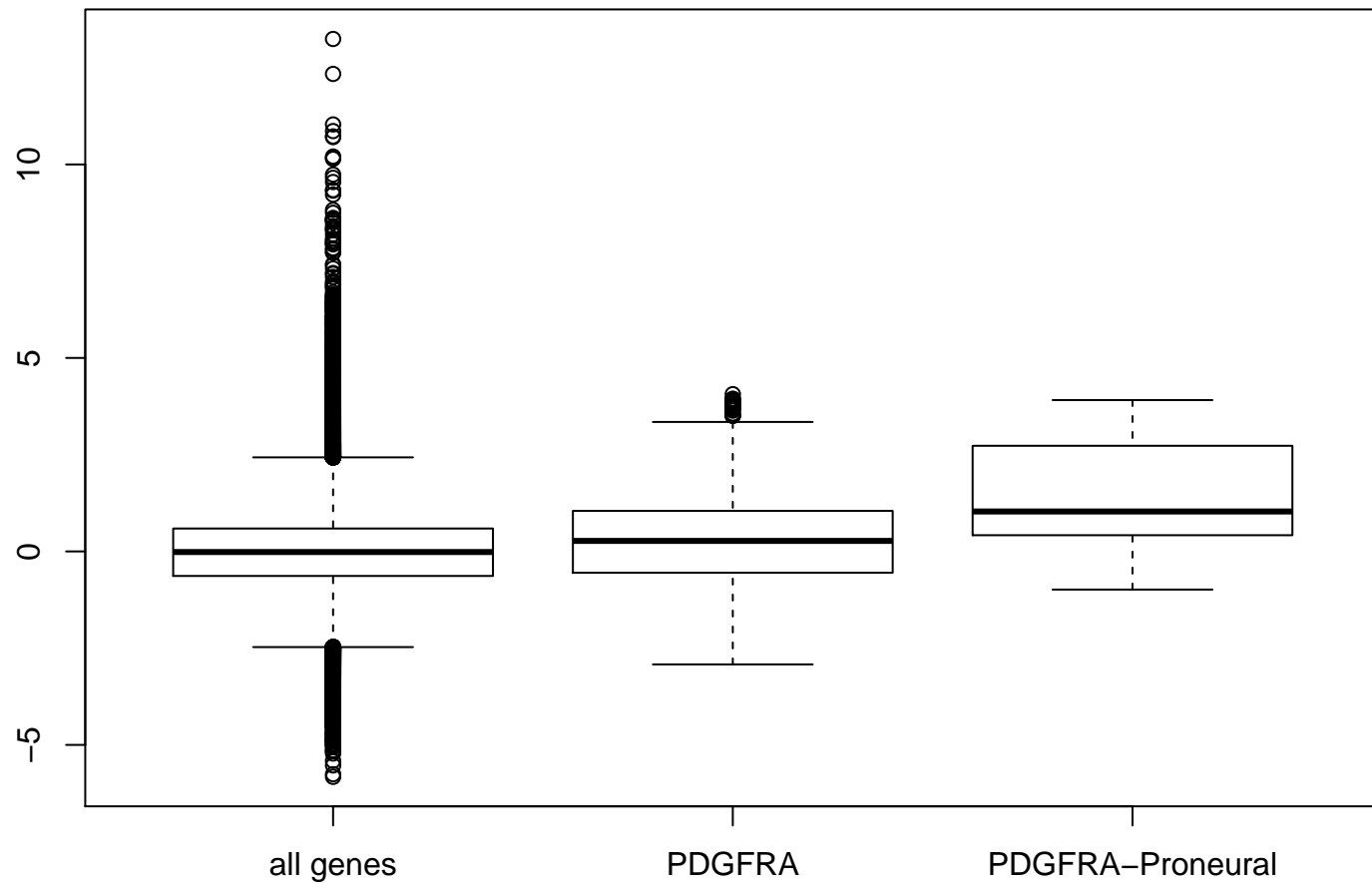

Supplement: Additional file 5 — (Proneural Heterogeneity vignette). [file 1471-2105-14-217-S5.gz › ProneuralHeterogeneity/inst/doc/ProneuralHeterogeneity-dist.mrna.pdf]

# Proneural Tumors Clustered on Gene Expression in Three KEGG Cancer-related Pathways

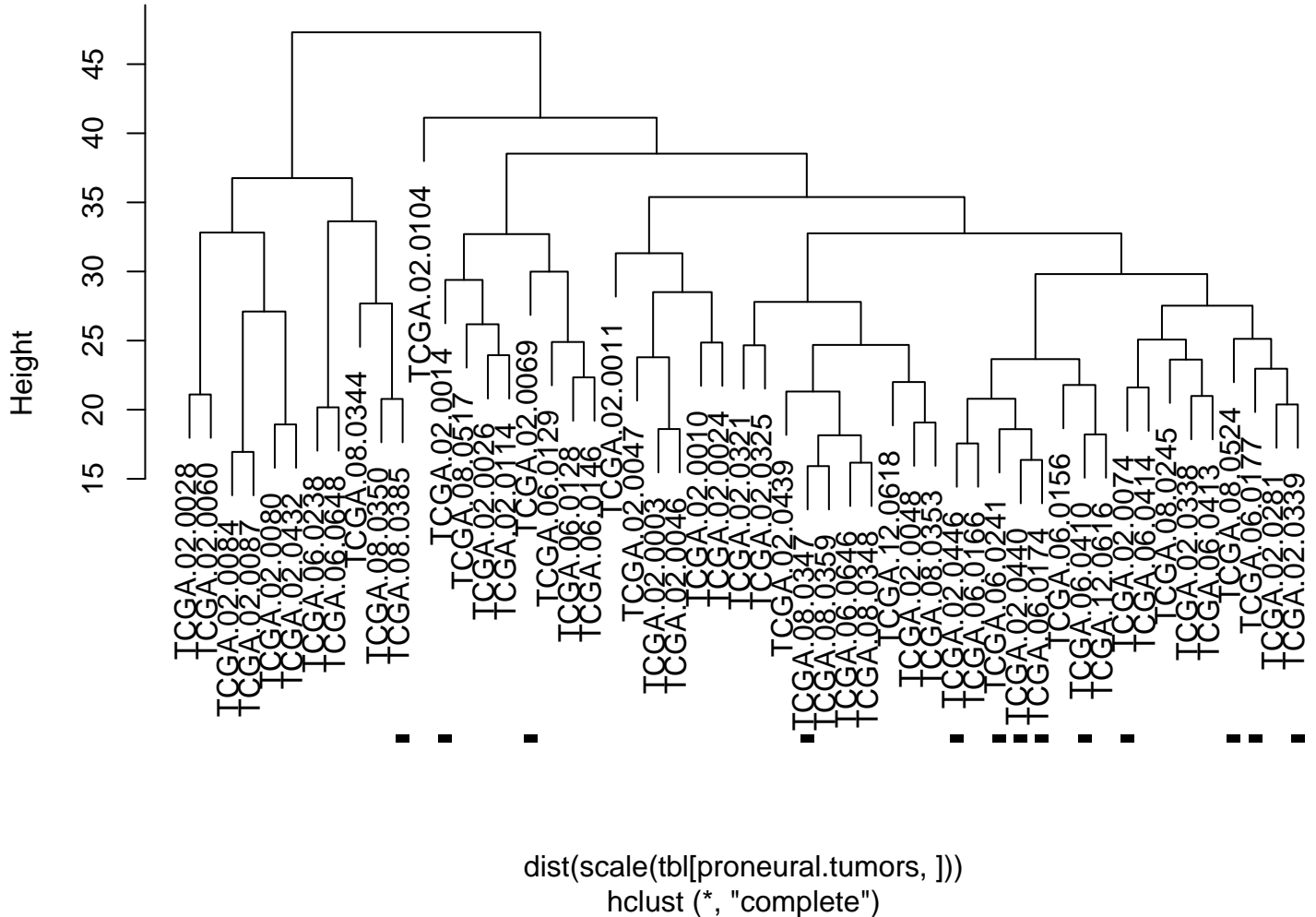

Supplement: Additional file 5 — (Proneural Heterogeneity vignette). [file 1471-2105-14-217-S5.gz › ProneuralHeterogeneity/inst/doc/ProneuralHeterogeneity-hclustWithStrongMarked.pdf]

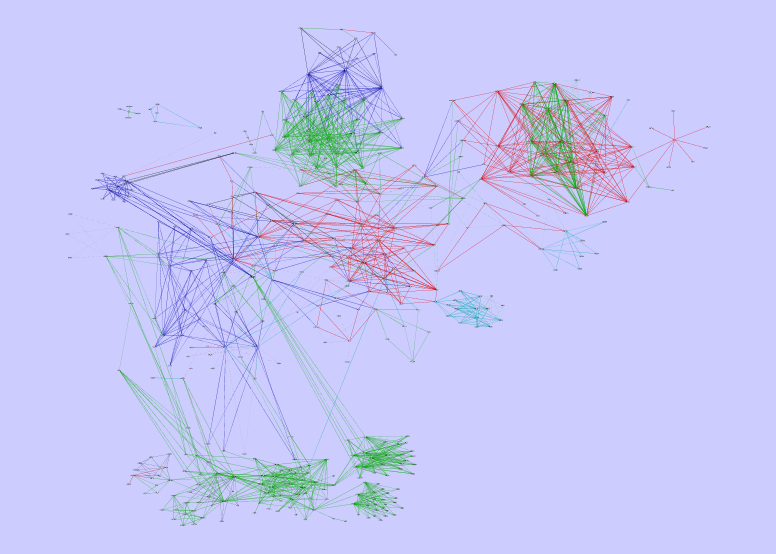

Supplement: Additional file 5 — (Proneural Heterogeneity vignette). [file 1471-2105-14-217-S5.gz › ProneuralHeterogeneity/inst/doc/fullNetwork.png]

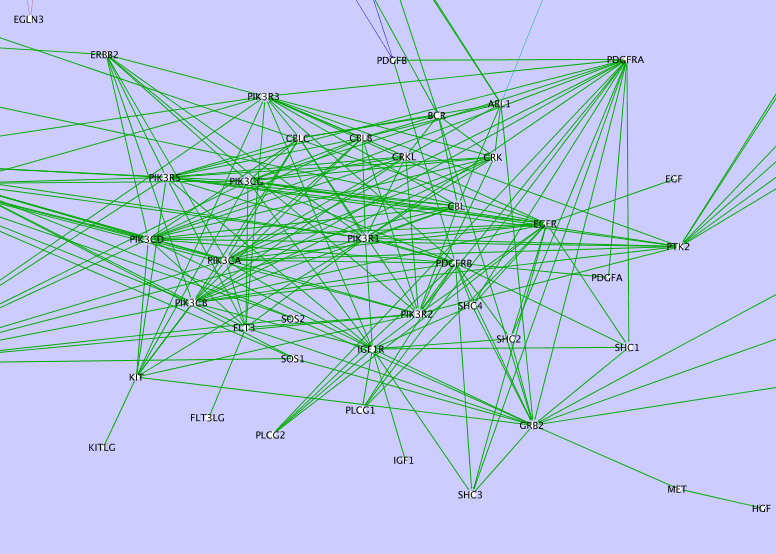

Supplement: Additional file 5 — (Proneural Heterogeneity vignette). [file 1471-2105-14-217-S5.gz › ProneuralHeterogeneity/inst/doc/smallNetwork.png]

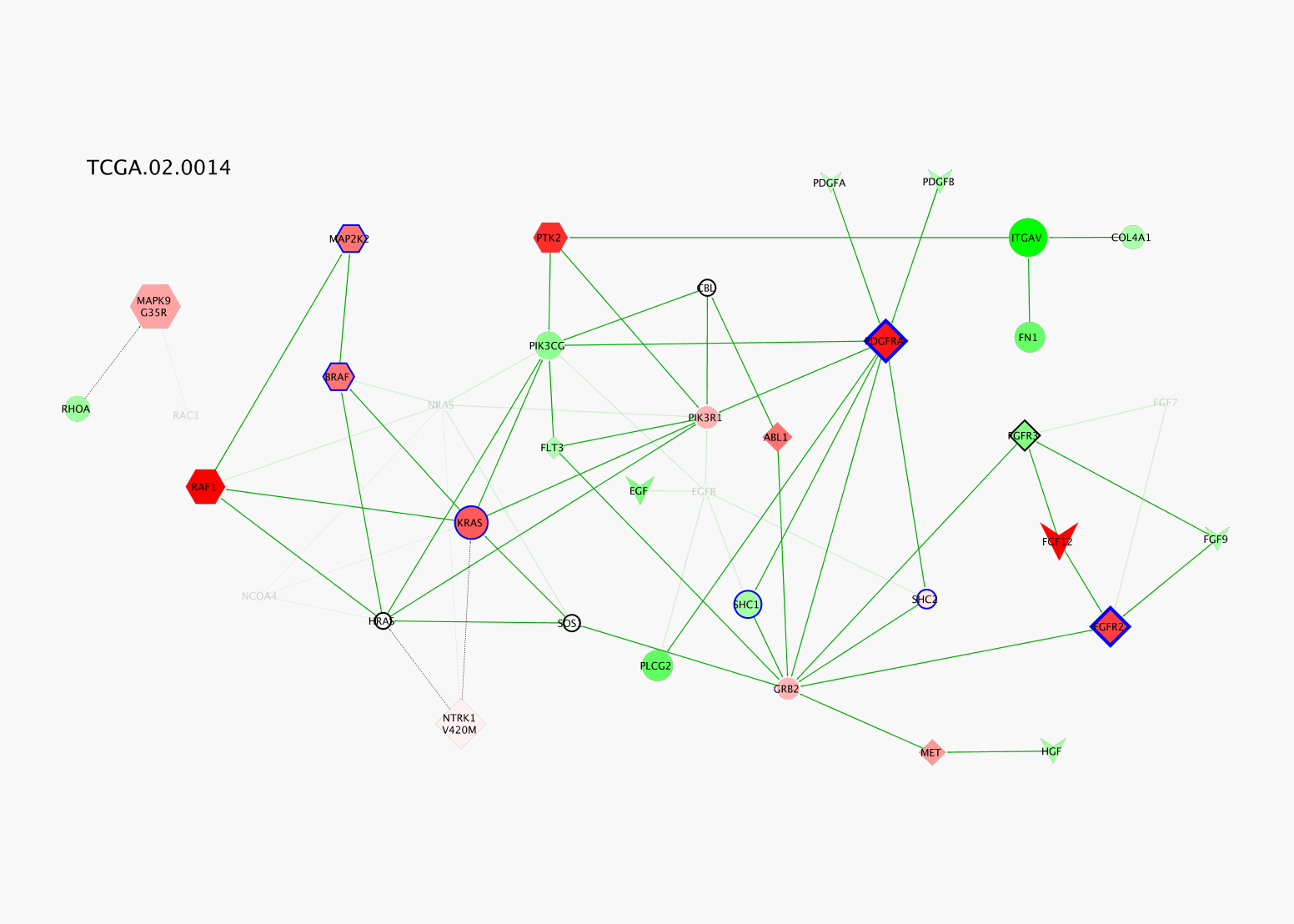

Supplement: Additional file 5 — (Proneural Heterogeneity vignette). [file 1471-2105-14-217-S5.gz › ProneuralHeterogeneity/inst/doc/tcga-02-0014.png]

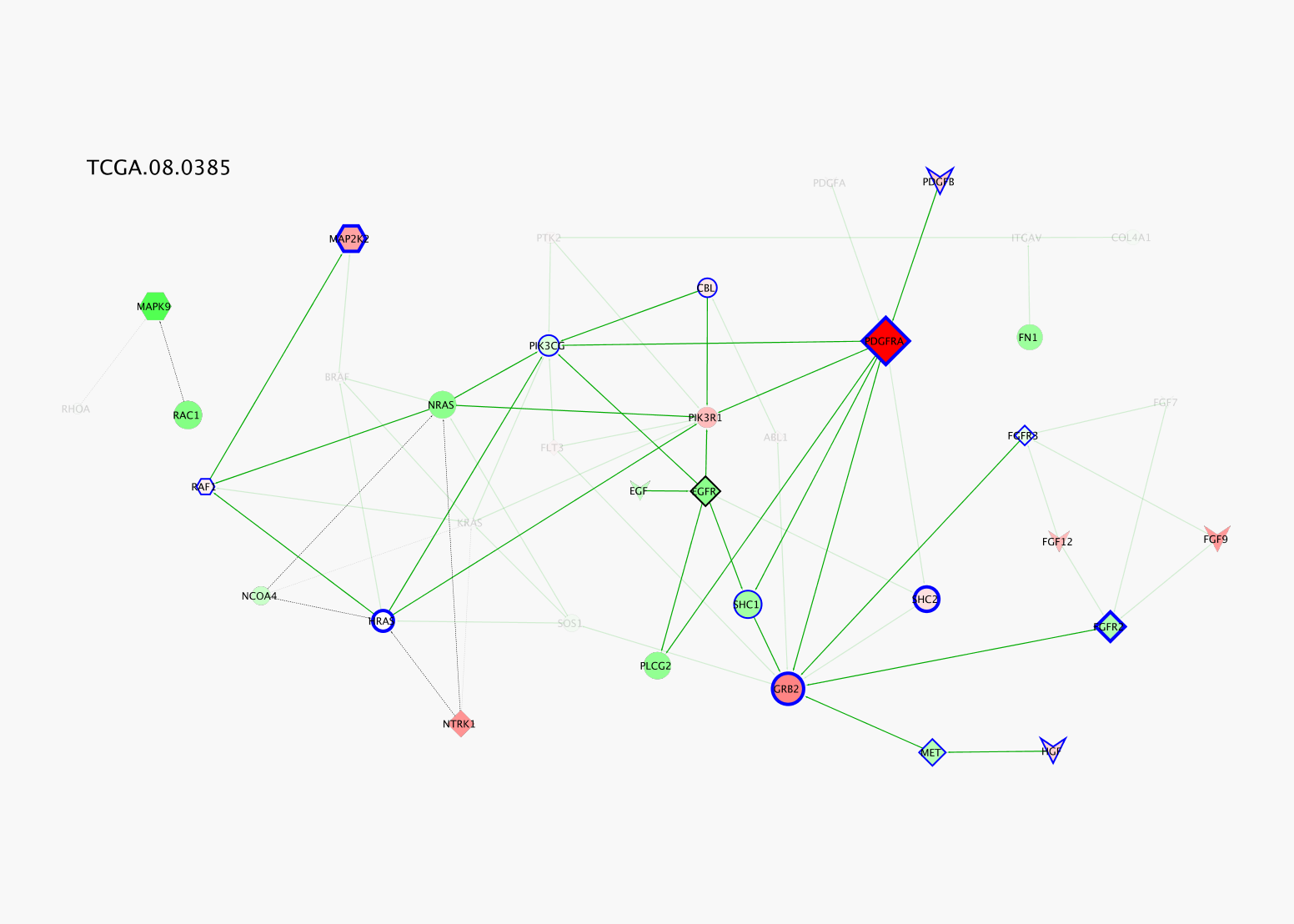

Supplement: Additional file 5 — (Proneural Heterogeneity vignette). [file 1471-2105-14-217-S5.gz › ProneuralHeterogeneity/inst/doc/tcga-08-0385.png]

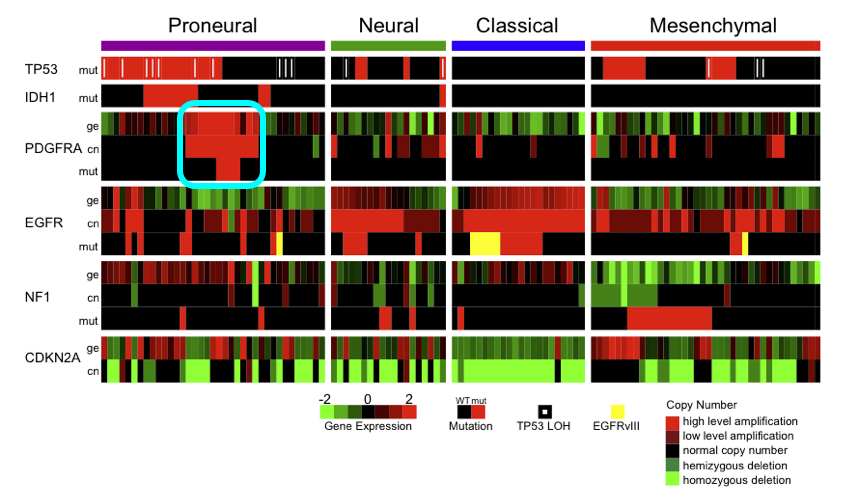

Supplement: Additional file 5 — (Proneural Heterogeneity vignette). [file 1471-2105-14-217-S5.gz › ProneuralHeterogeneity/inst/doc/verhaakClassification.png]

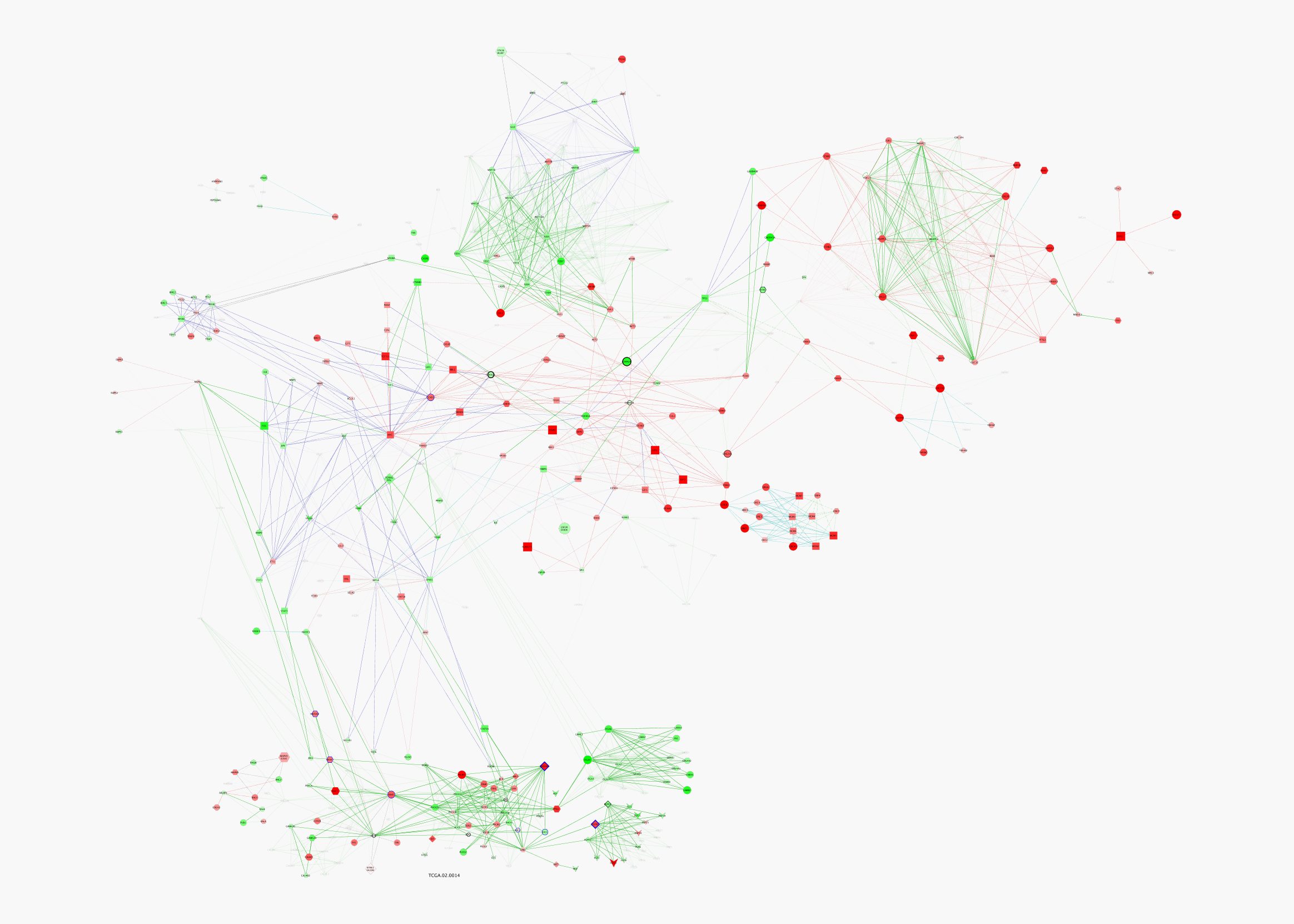

Supplement: Additional file 5 — (Proneural Heterogeneity vignette). [file 1471-2105-14-217-S5.gz › ProneuralHeterogeneity/inst/extdata/TCGA.02.0014-full.png]

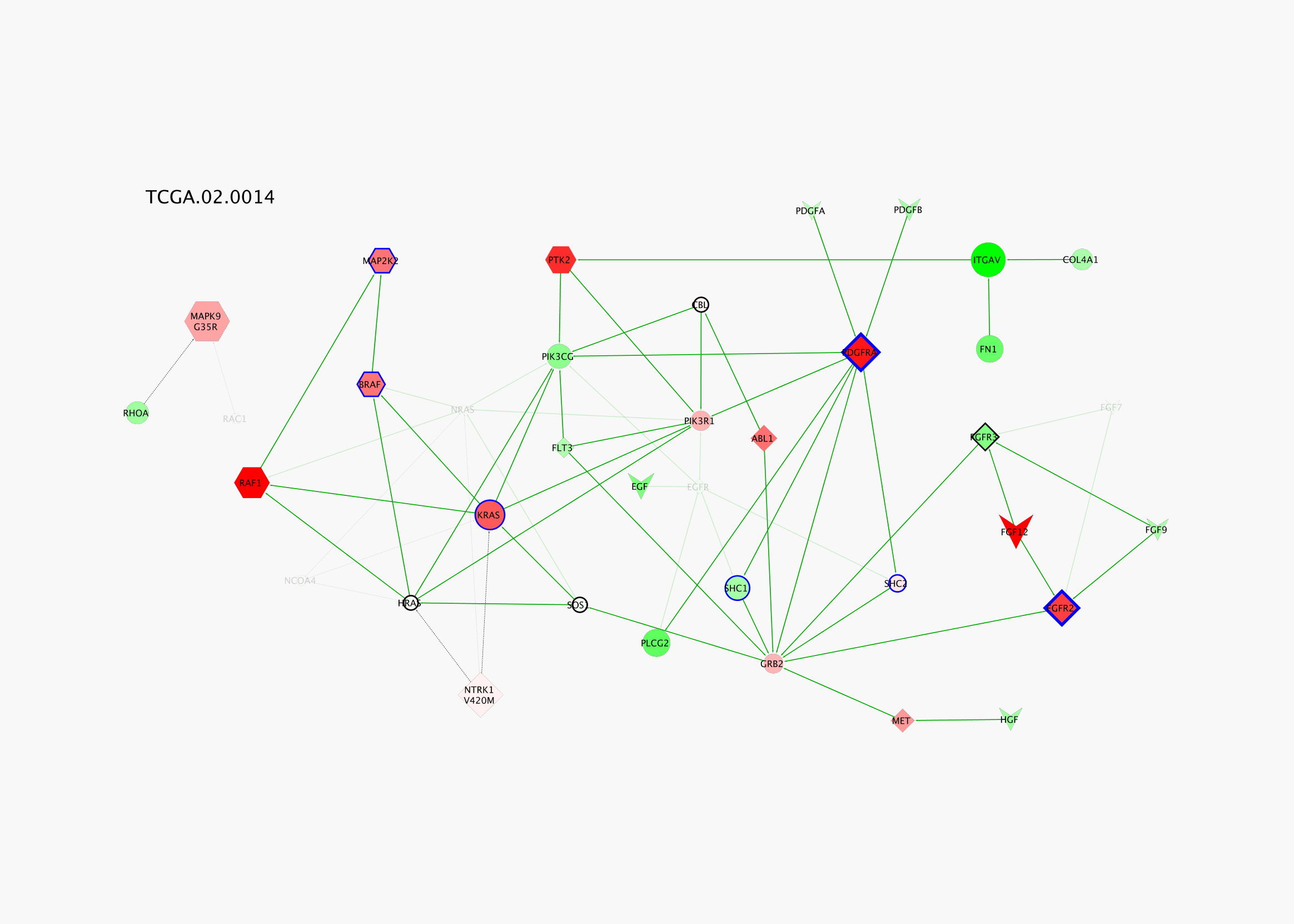

Supplement: Additional file 5 — (Proneural Heterogeneity vignette). [file 1471-2105-14-217-S5.gz › ProneuralHeterogeneity/inst/extdata/TCGA.02.0014.png]

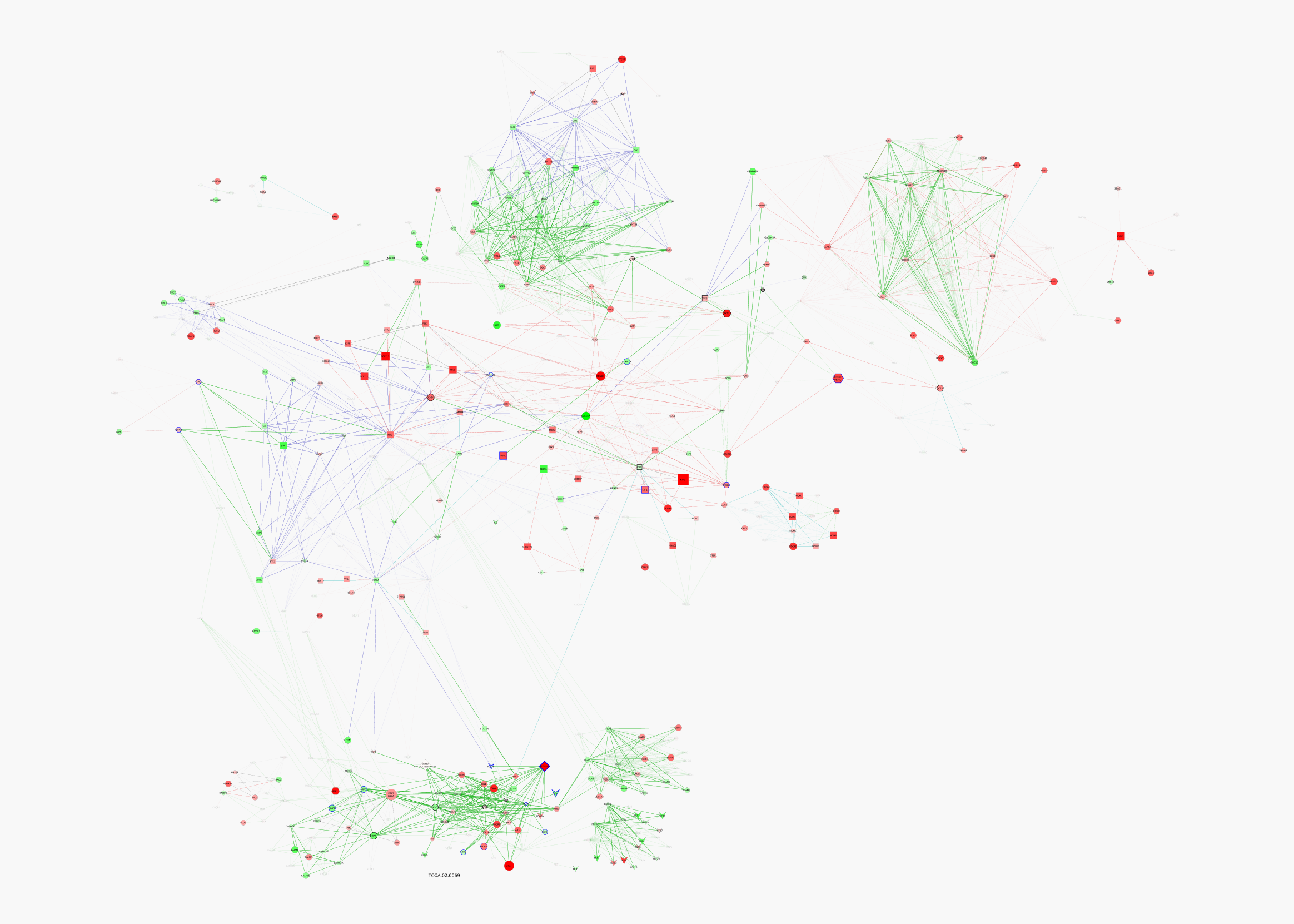

Supplement: Additional file 5 — (Proneural Heterogeneity vignette). [file 1471-2105-14-217-S5.gz › ProneuralHeterogeneity/inst/extdata/TCGA.02.0069-full.png]

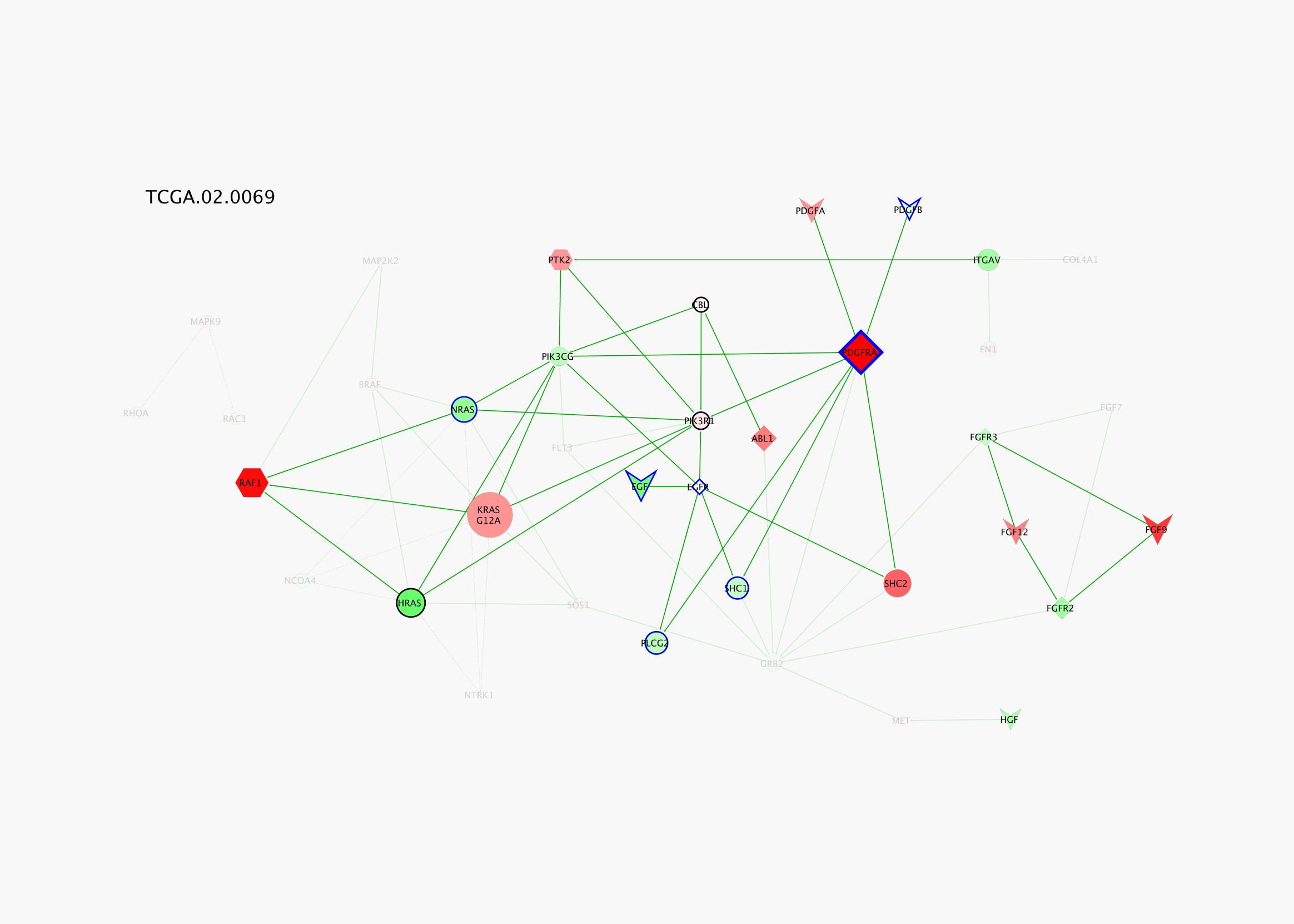

Supplement: Additional file 5 — (Proneural Heterogeneity vignette). [file 1471-2105-14-217-S5.gz › ProneuralHeterogeneity/inst/extdata/TCGA.02.0069.png]

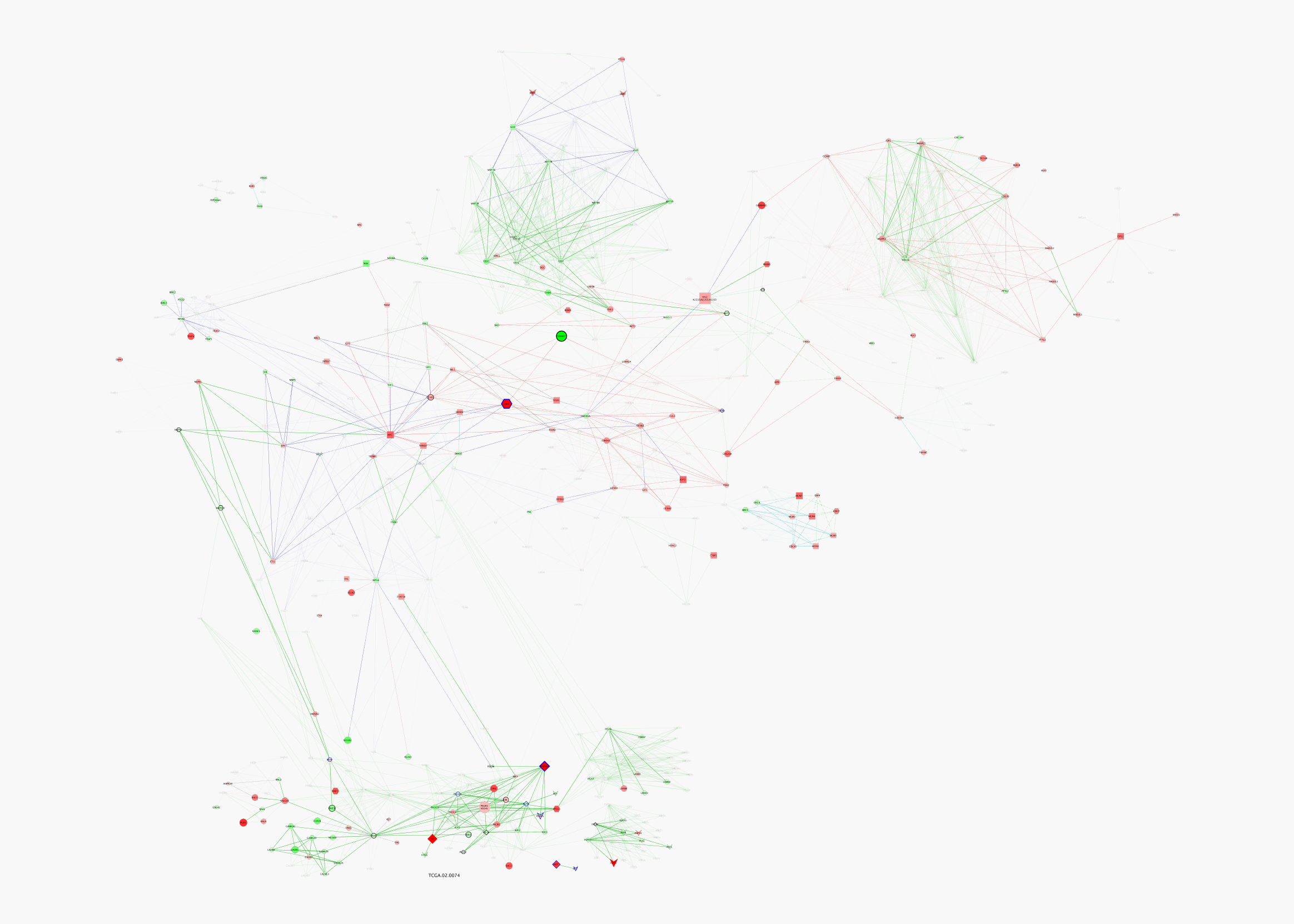

Supplement: Additional file 5 — (Proneural Heterogeneity vignette). [file 1471-2105-14-217-S5.gz › ProneuralHeterogeneity/inst/extdata/TCGA.02.0074-full.png]

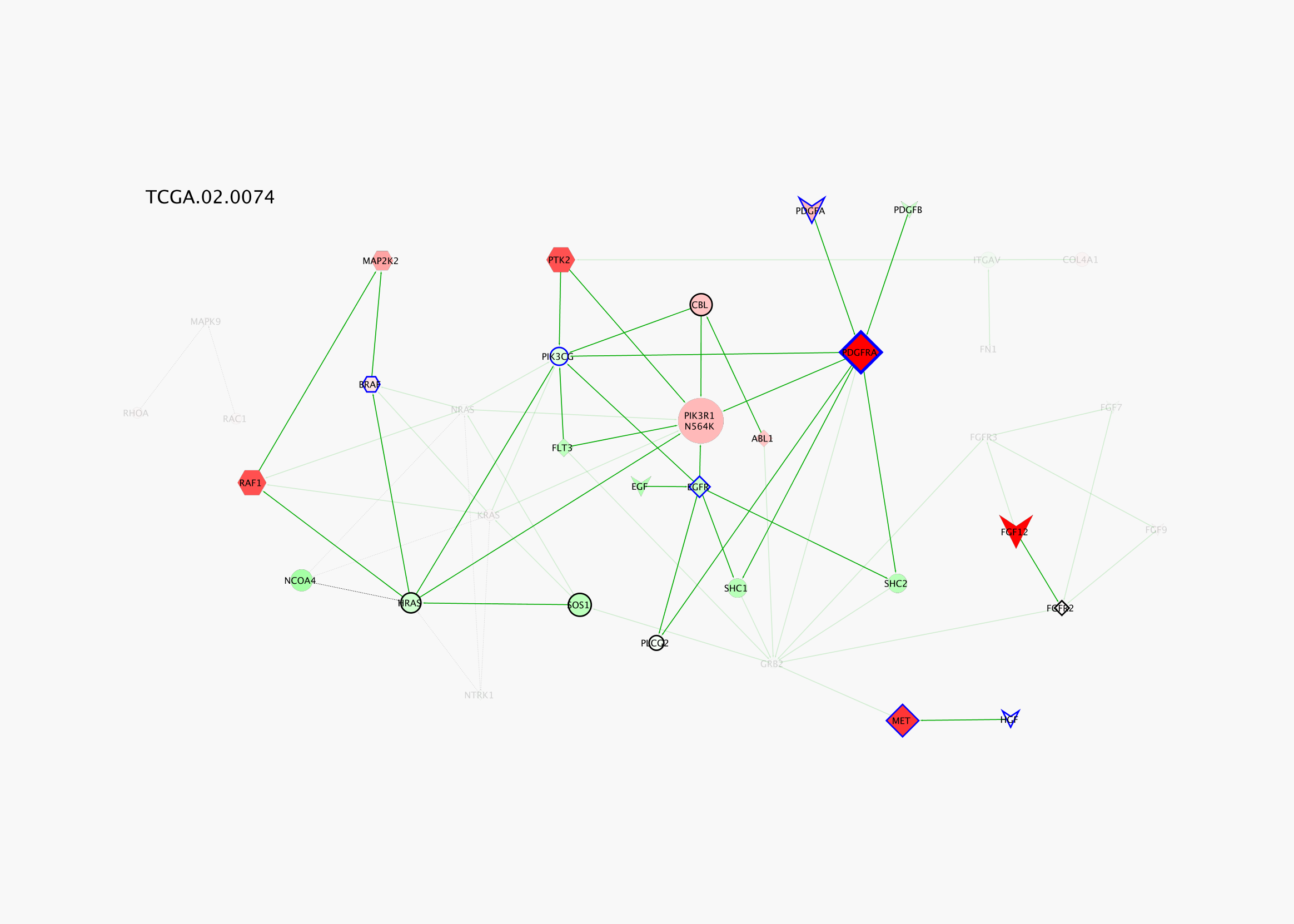

Supplement: Additional file 5 — (Proneural Heterogeneity vignette). [file 1471-2105-14-217-S5.gz › ProneuralHeterogeneity/inst/extdata/TCGA.02.0074.png]

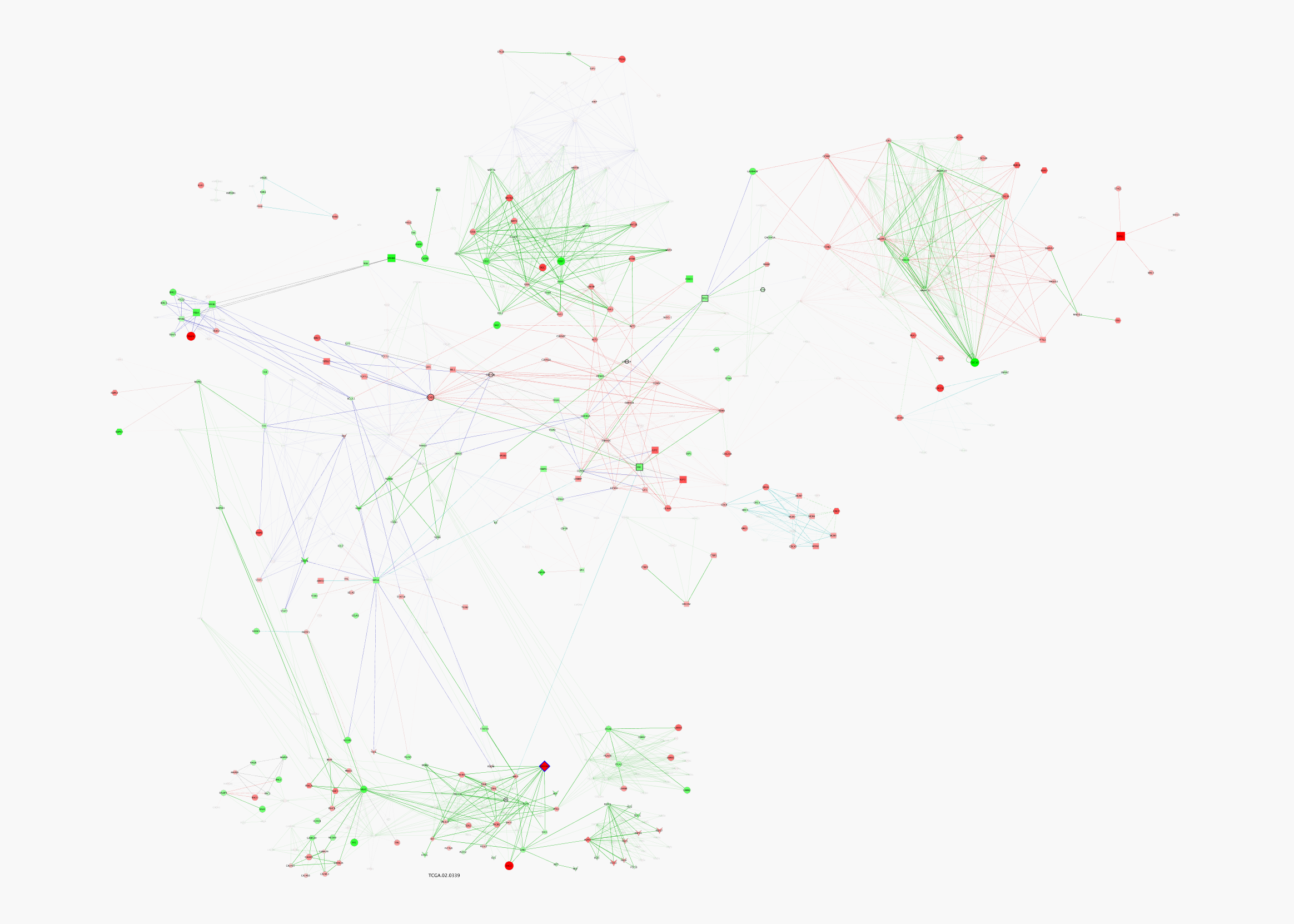

Supplement: Additional file 5 — (Proneural Heterogeneity vignette). [file 1471-2105-14-217-S5.gz › ProneuralHeterogeneity/inst/extdata/TCGA.02.0339-full.png]

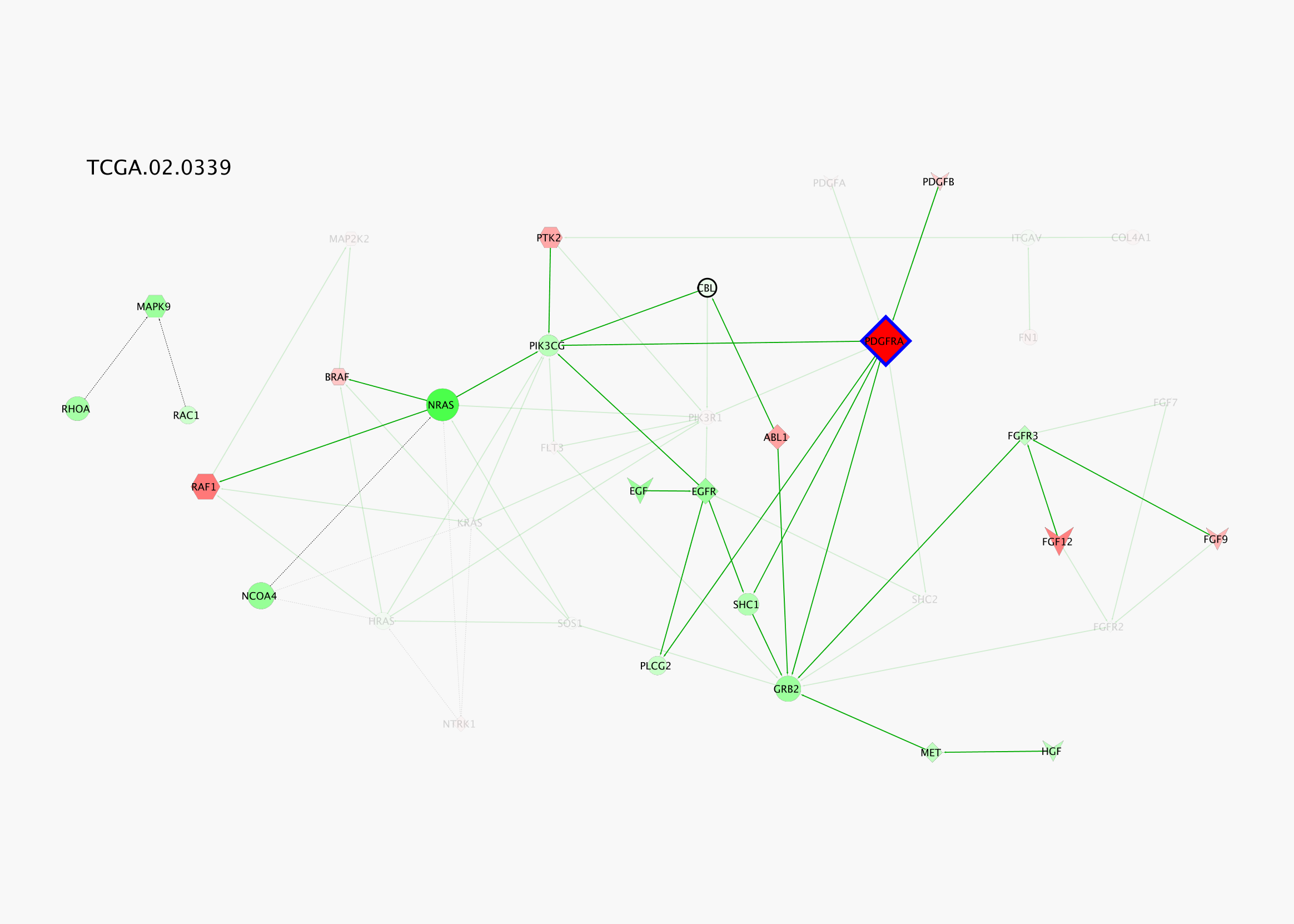

Supplement: Additional file 5 — (Proneural Heterogeneity vignette). [file 1471-2105-14-217-S5.gz › ProneuralHeterogeneity/inst/extdata/TCGA.02.0339.png]

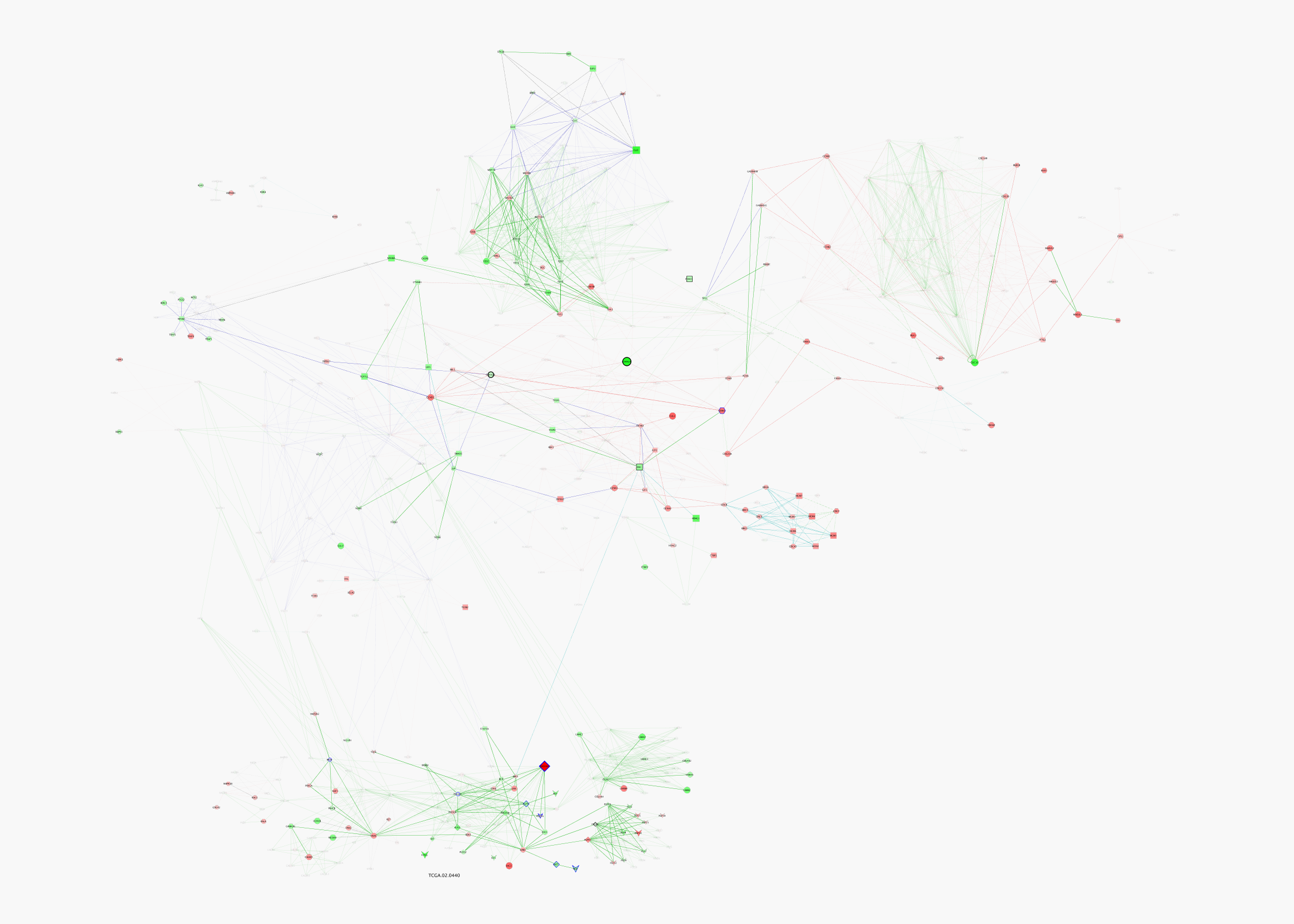

Supplement: Additional file 5 — (Proneural Heterogeneity vignette). [file 1471-2105-14-217-S5.gz › ProneuralHeterogeneity/inst/extdata/TCGA.02.0440-full.png]

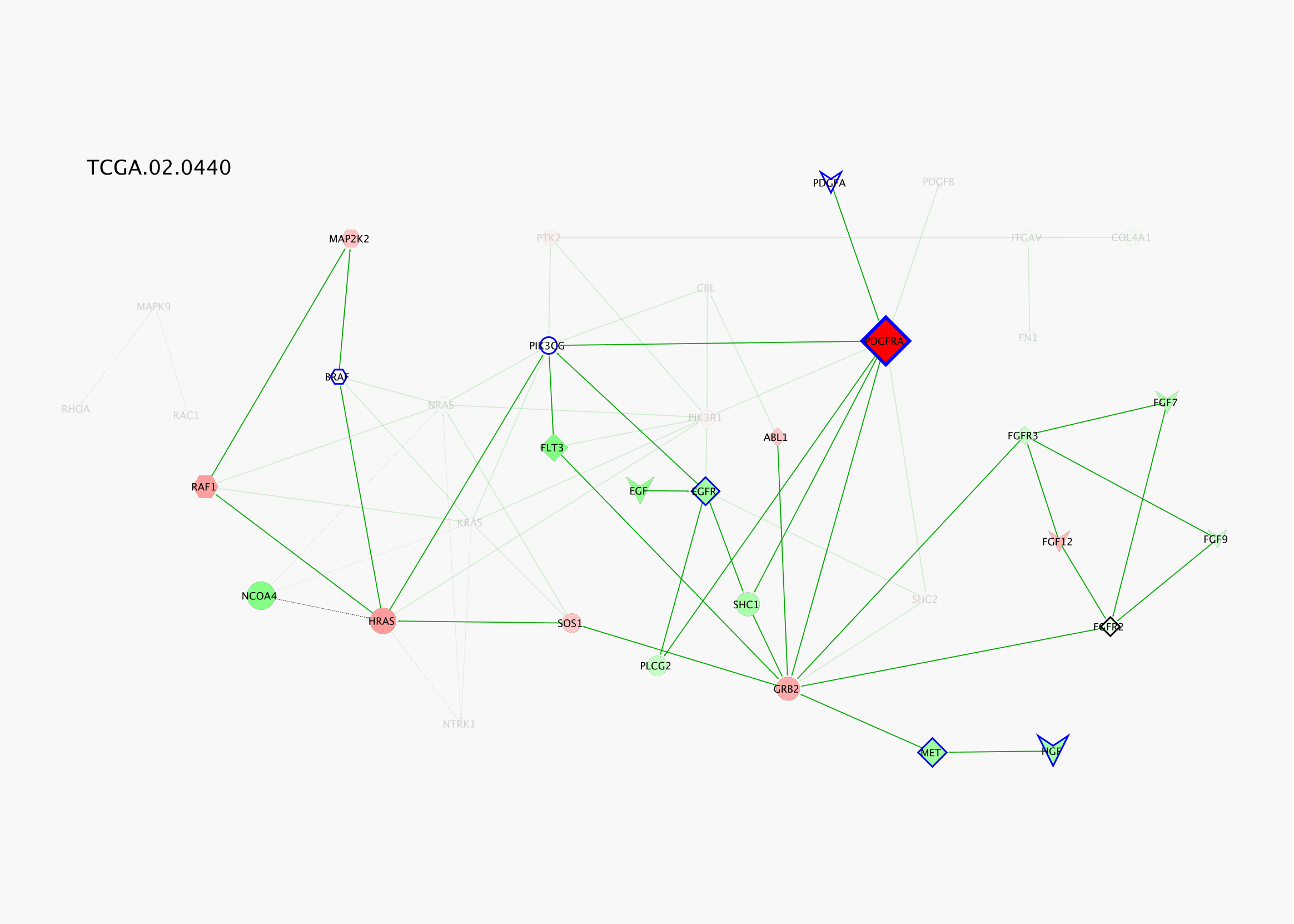

Supplement: Additional file 5 — (Proneural Heterogeneity vignette). [file 1471-2105-14-217-S5.gz › ProneuralHeterogeneity/inst/extdata/TCGA.02.0440.png]

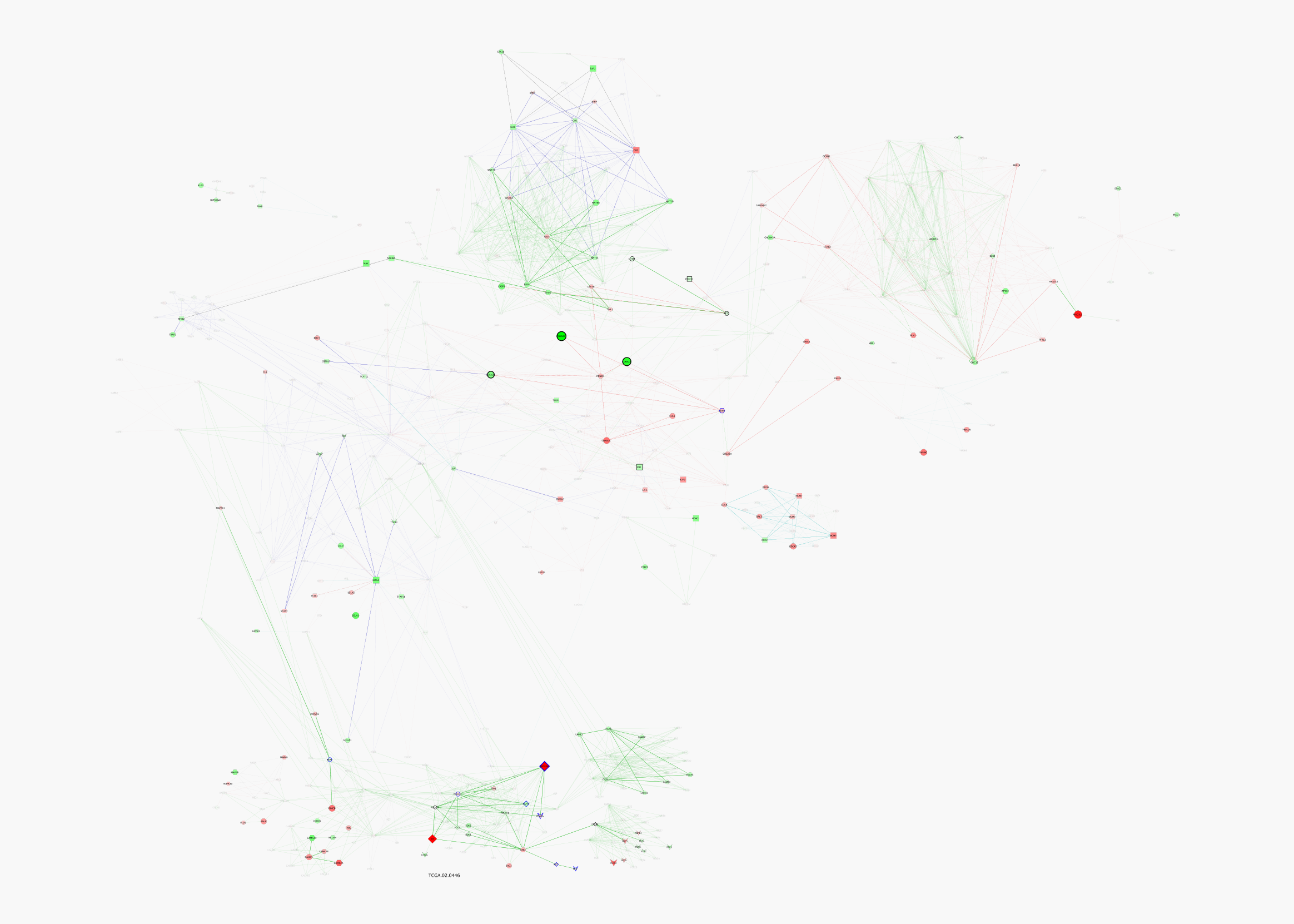

Supplement: Additional file 5 — (Proneural Heterogeneity vignette). [file 1471-2105-14-217-S5.gz › ProneuralHeterogeneity/inst/extdata/TCGA.02.0446-full.png]

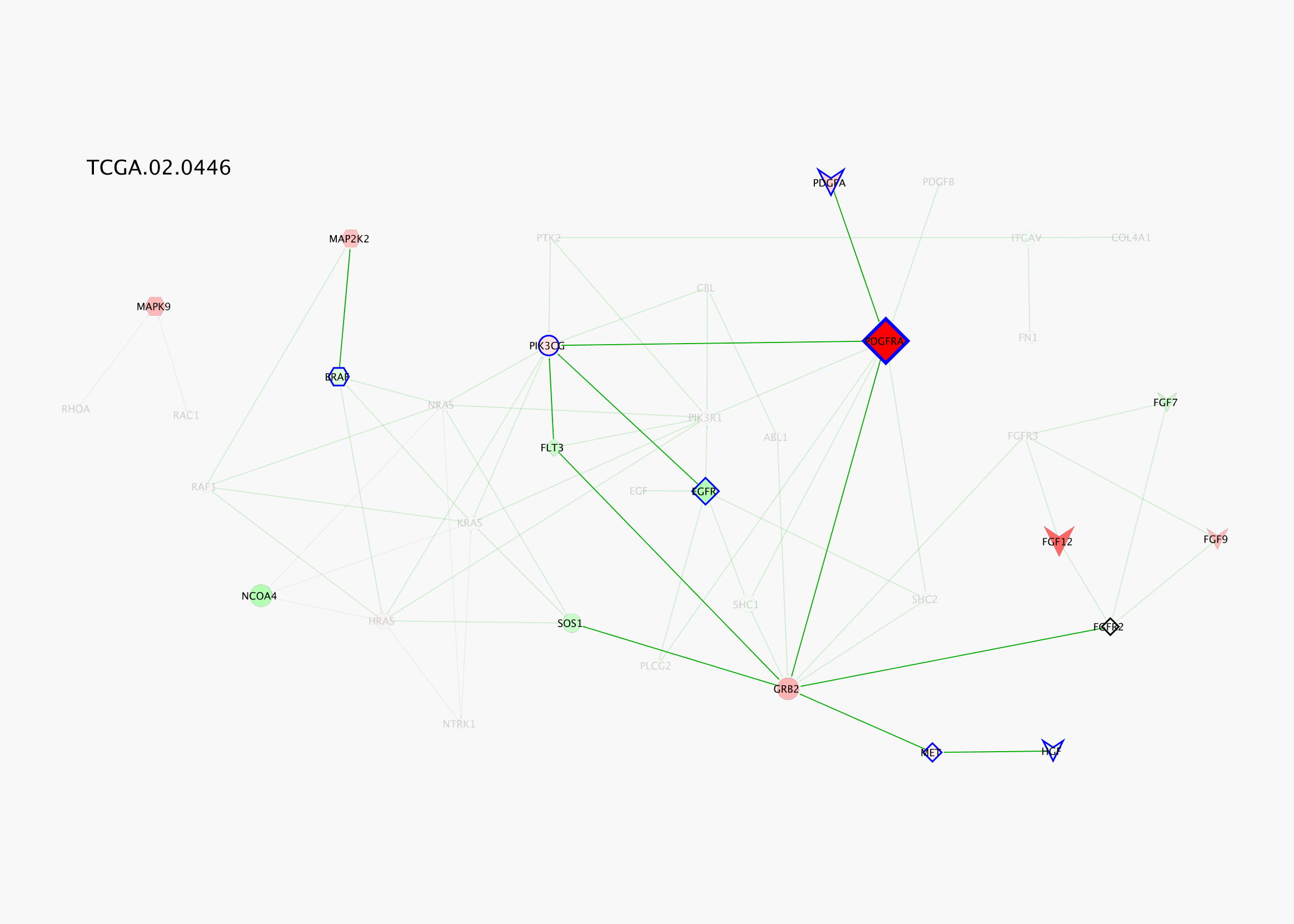

Supplement: Additional file 5 — (Proneural Heterogeneity vignette). [file 1471-2105-14-217-S5.gz › ProneuralHeterogeneity/inst/extdata/TCGA.02.0446.png]

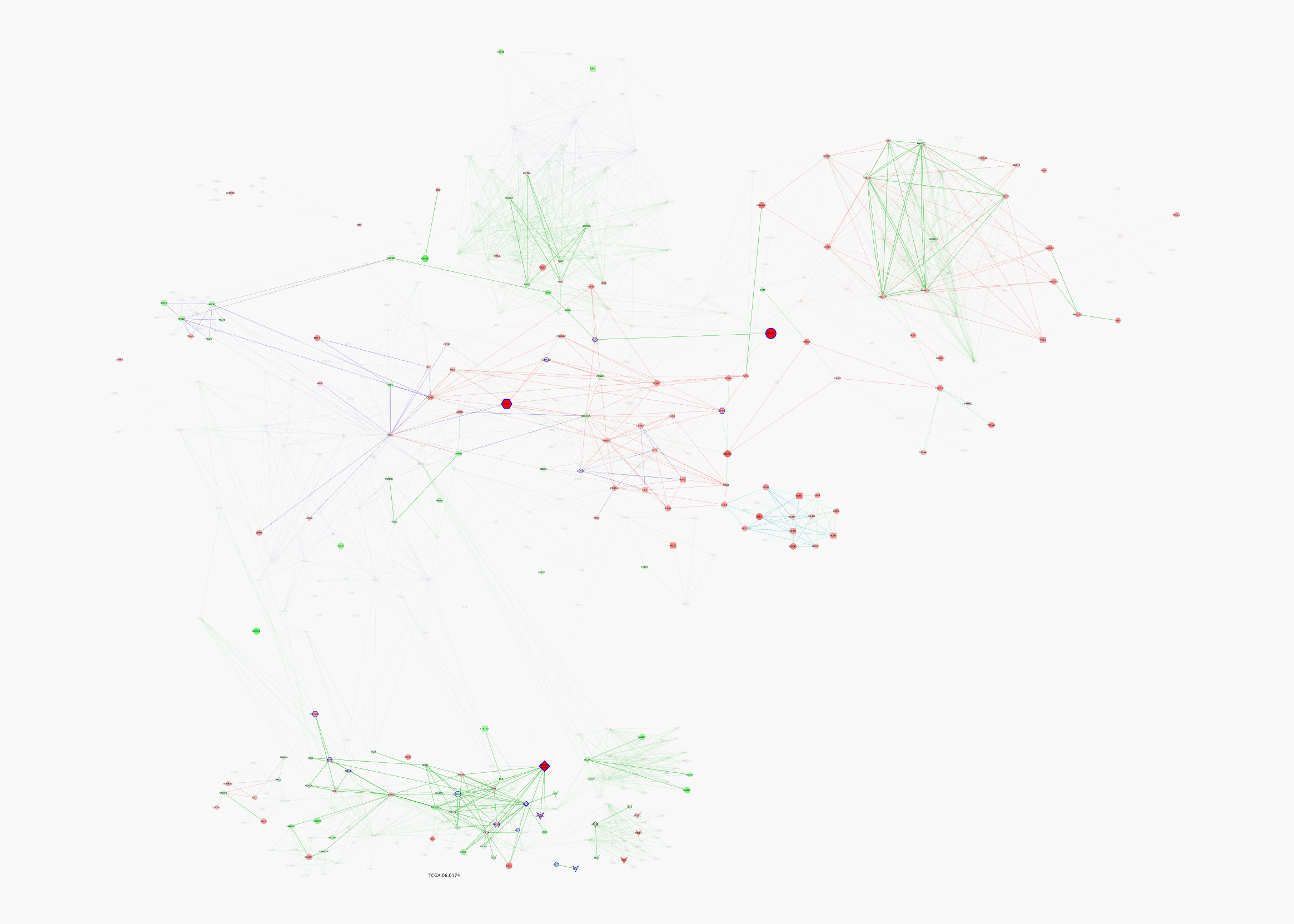

Supplement: Additional file 5 — (Proneural Heterogeneity vignette). [file 1471-2105-14-217-S5.gz › ProneuralHeterogeneity/inst/extdata/TCGA.06.0174-full.png]

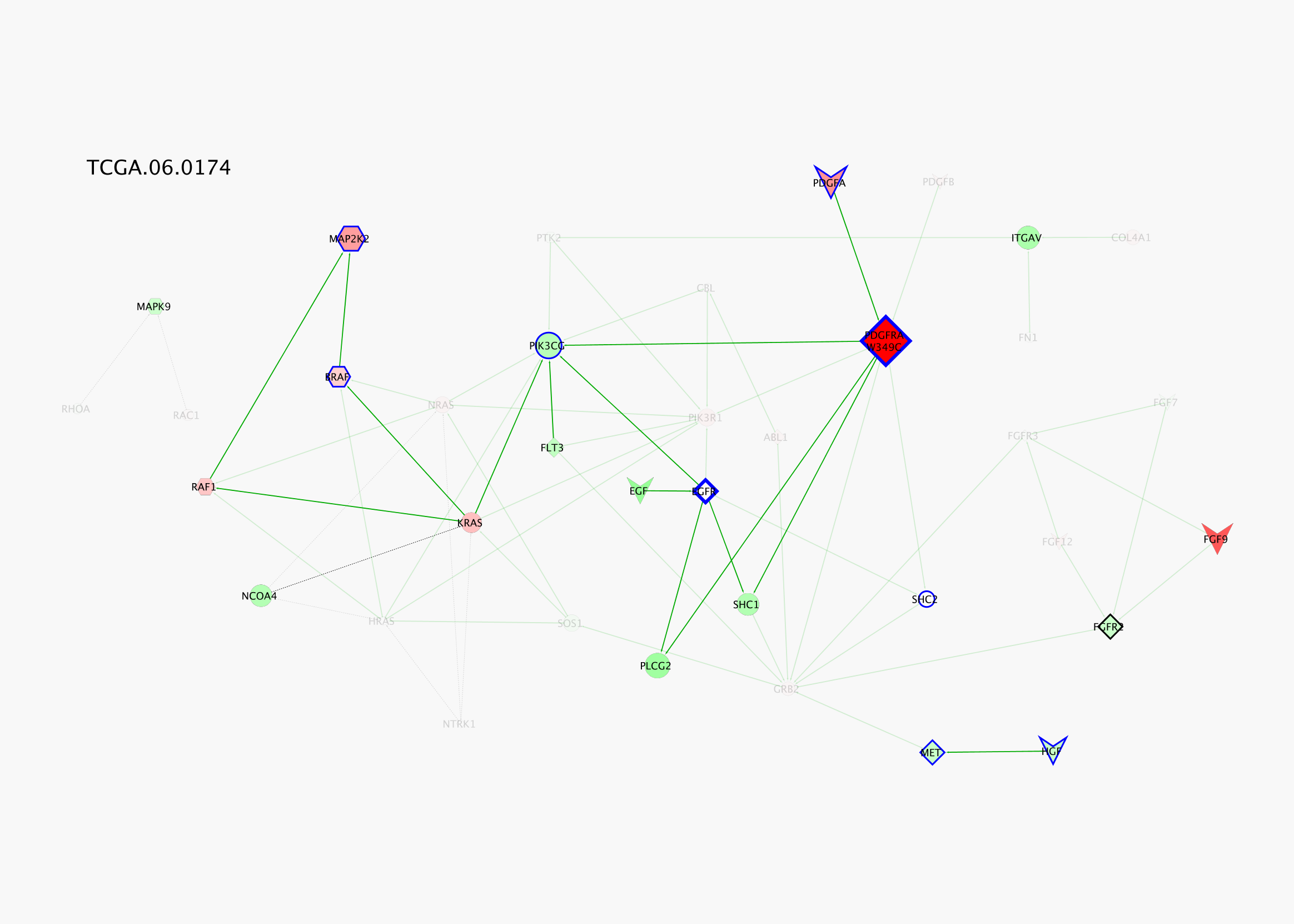

Supplement: Additional file 5 — (Proneural Heterogeneity vignette). [file 1471-2105-14-217-S5.gz › ProneuralHeterogeneity/inst/extdata/TCGA.06.0174.png]

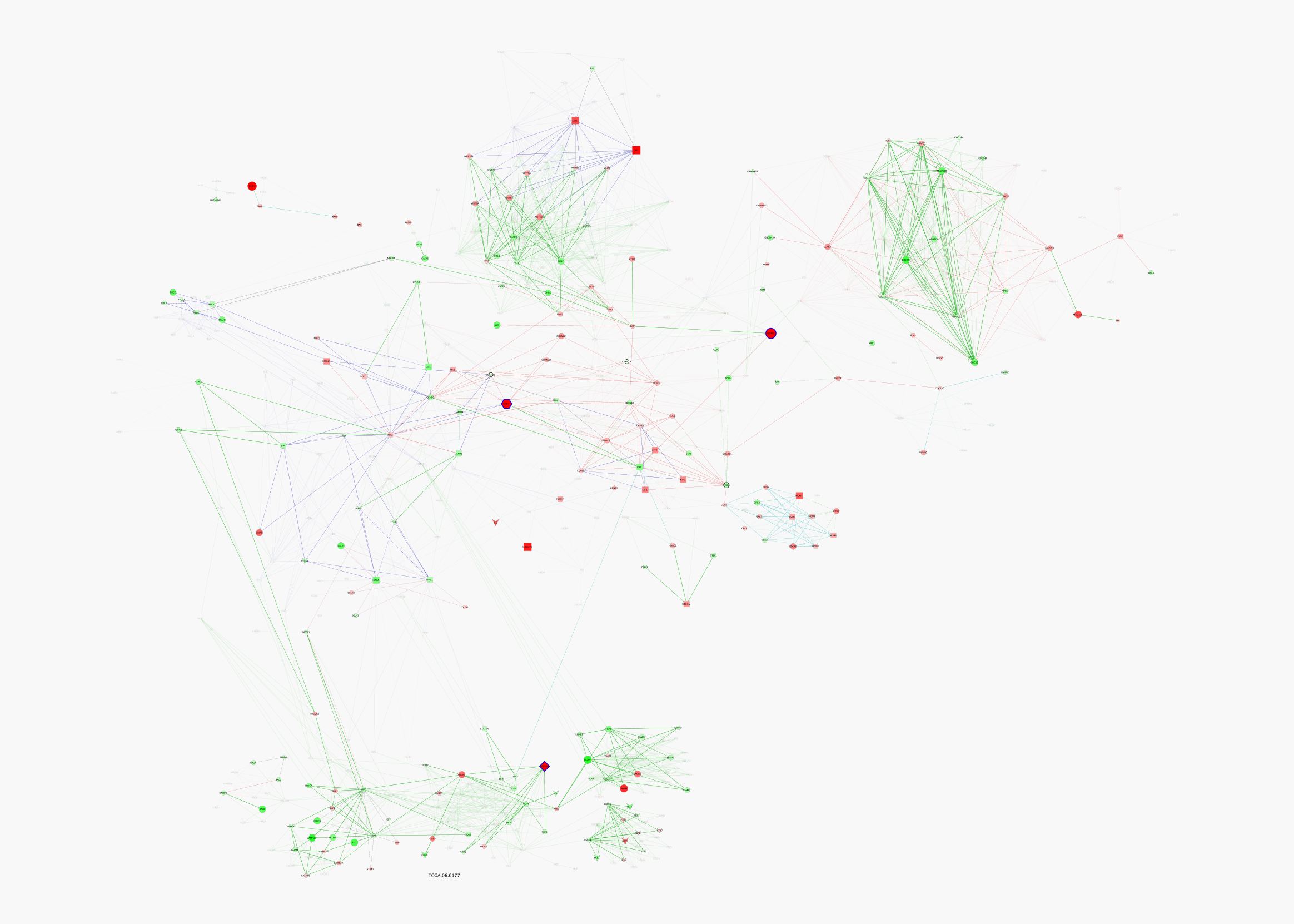

Supplement: Additional file 5 — (Proneural Heterogeneity vignette). [file 1471-2105-14-217-S5.gz › ProneuralHeterogeneity/inst/extdata/TCGA.06.0177-full.png]

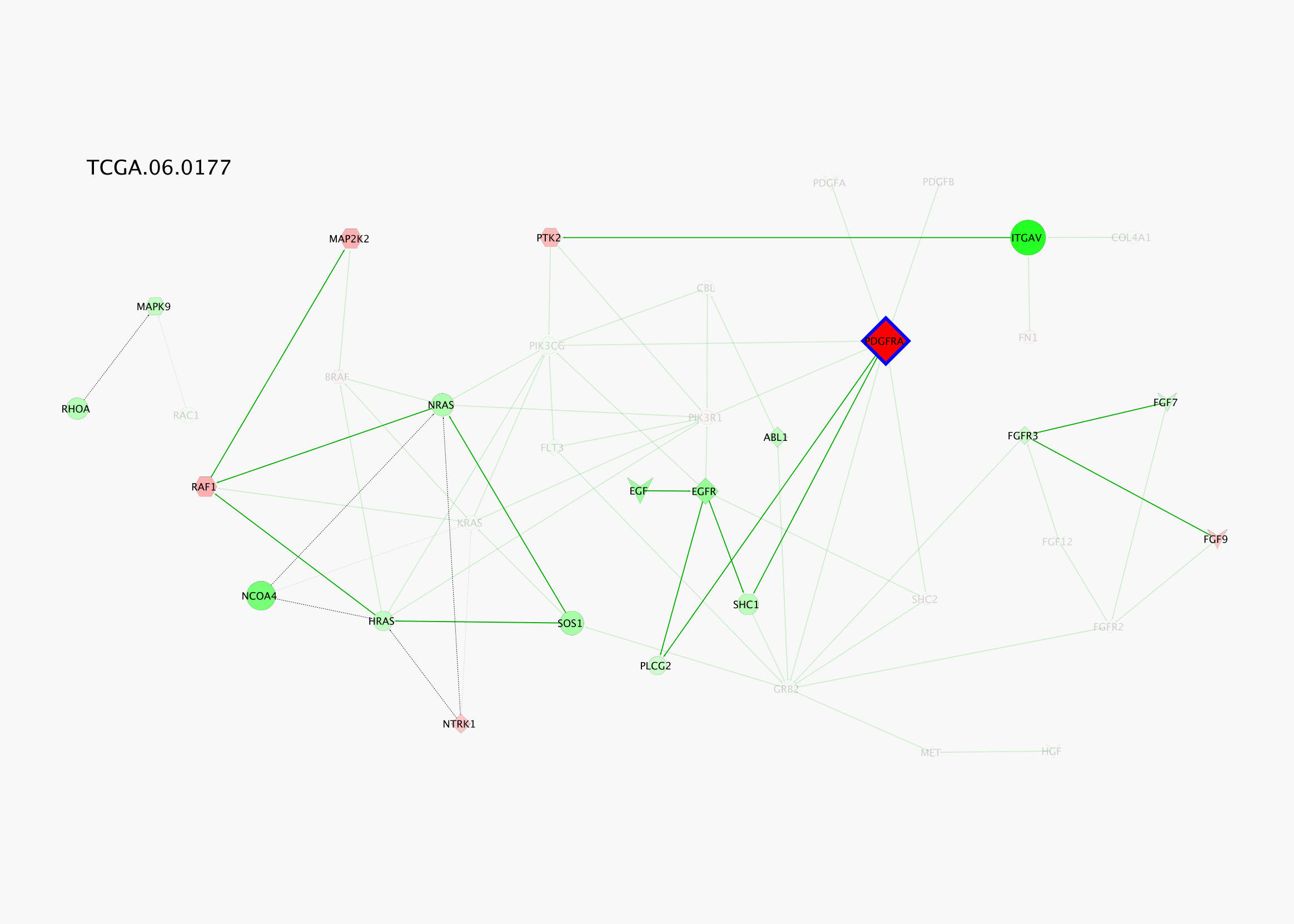

Supplement: Additional file 5 — (Proneural Heterogeneity vignette). [file 1471-2105-14-217-S5.gz › ProneuralHeterogeneity/inst/extdata/TCGA.06.0177.png]

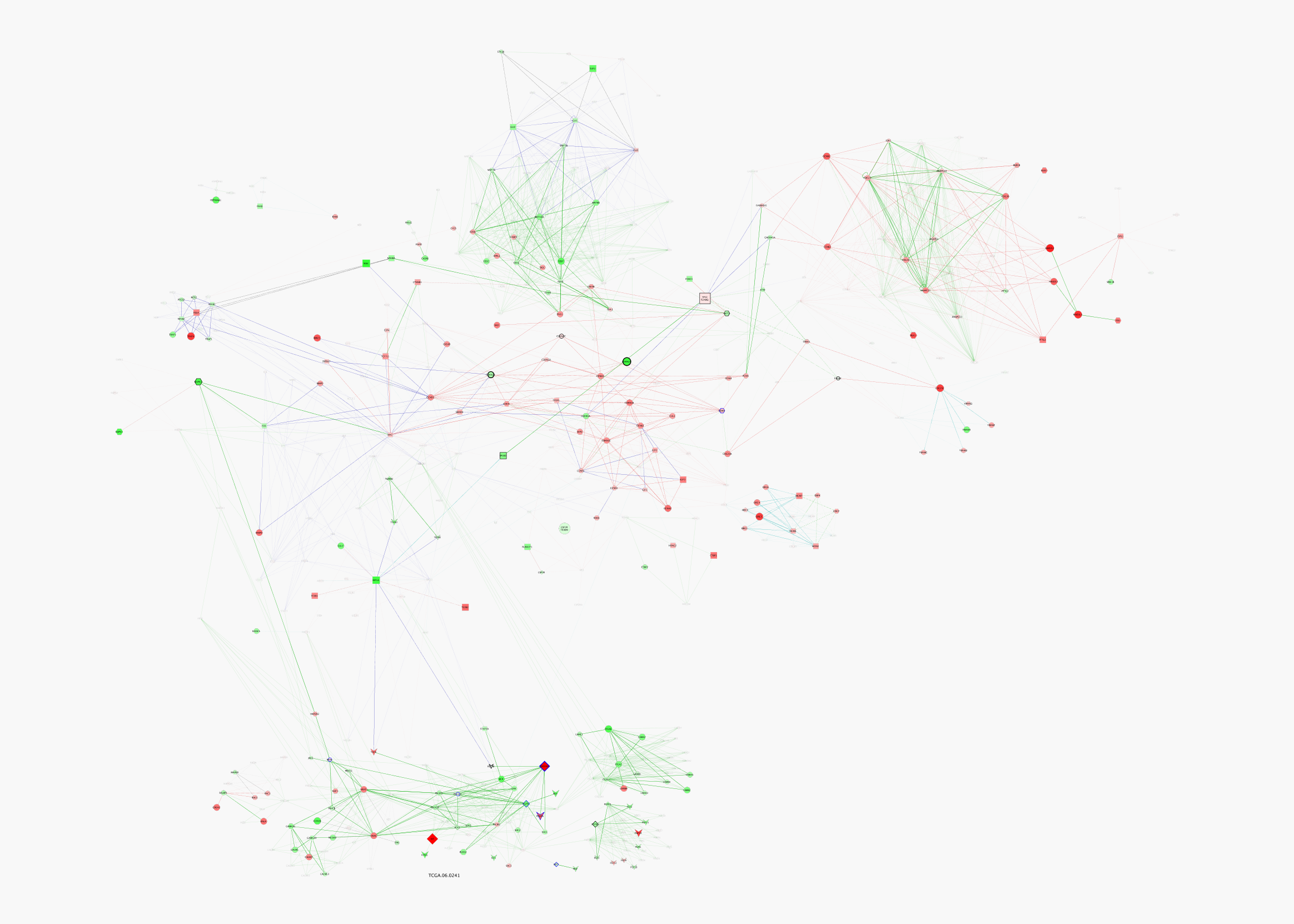

Supplement: Additional file 5 — (Proneural Heterogeneity vignette). [file 1471-2105-14-217-S5.gz › ProneuralHeterogeneity/inst/extdata/TCGA.06.0241-full.png]

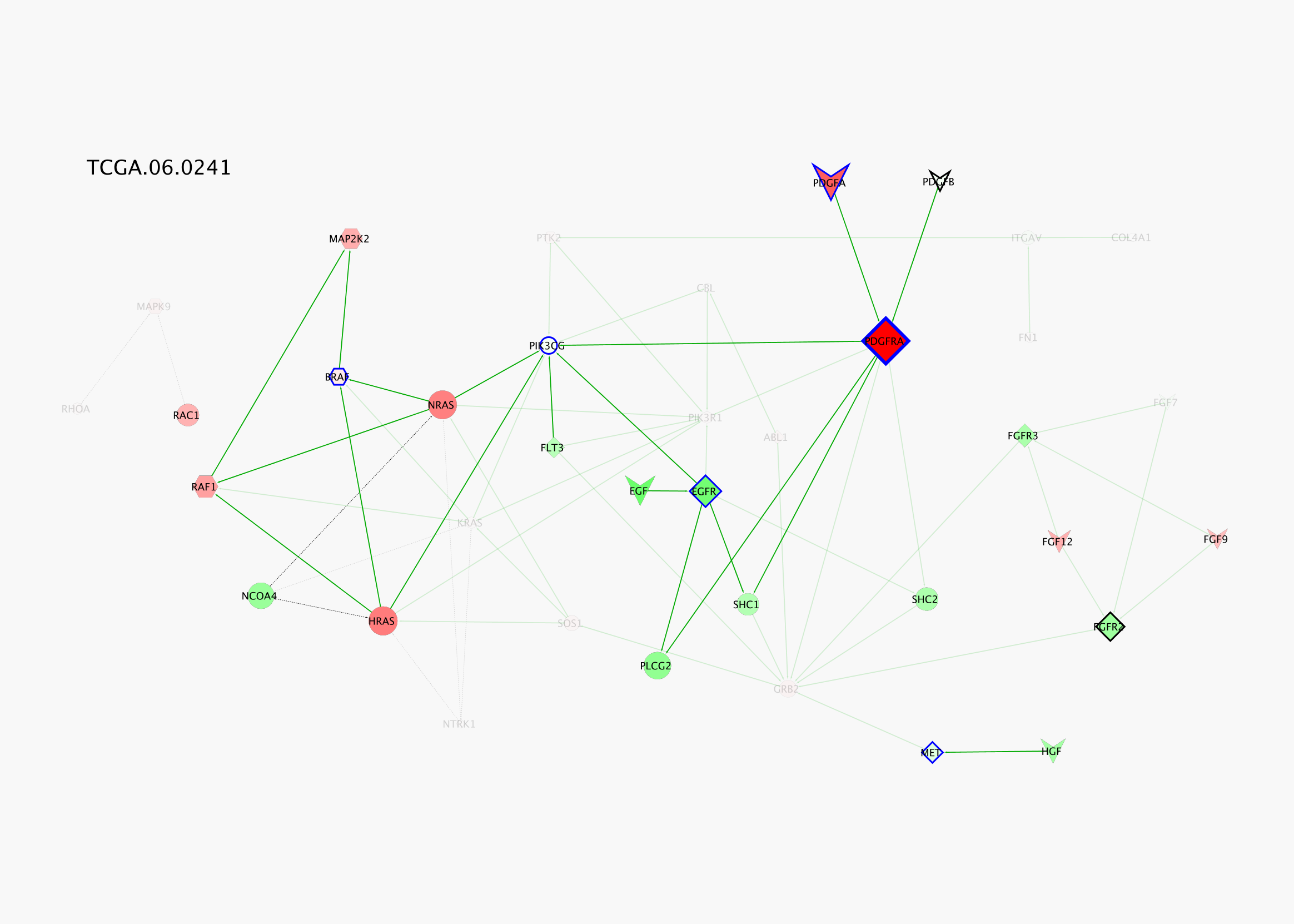

Supplement: Additional file 5 — (Proneural Heterogeneity vignette). [file 1471-2105-14-217-S5.gz › ProneuralHeterogeneity/inst/extdata/TCGA.06.0241.png]

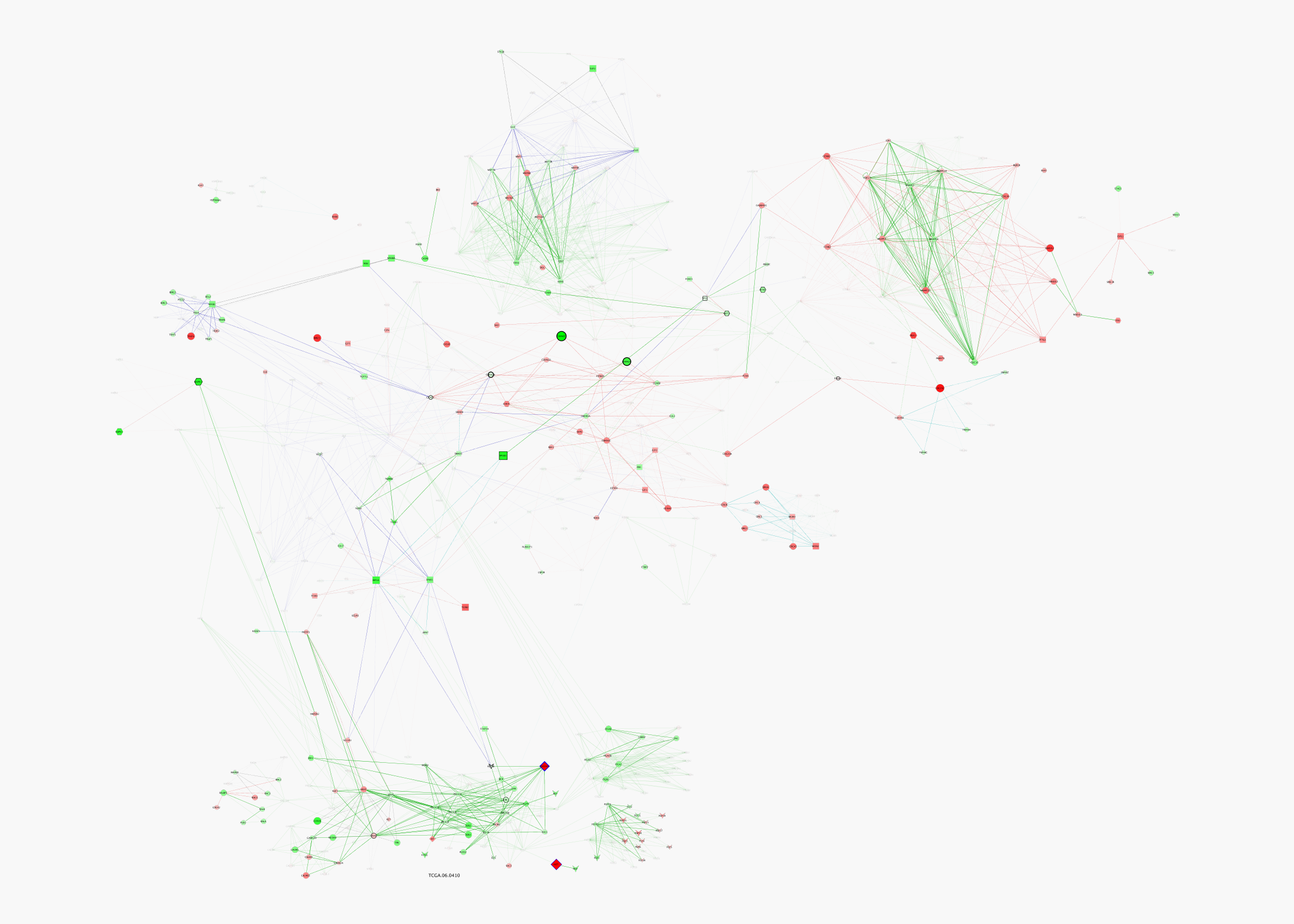

Supplement: Additional file 5 — (Proneural Heterogeneity vignette). [file 1471-2105-14-217-S5.gz › ProneuralHeterogeneity/inst/extdata/TCGA.06.0410-full.png]

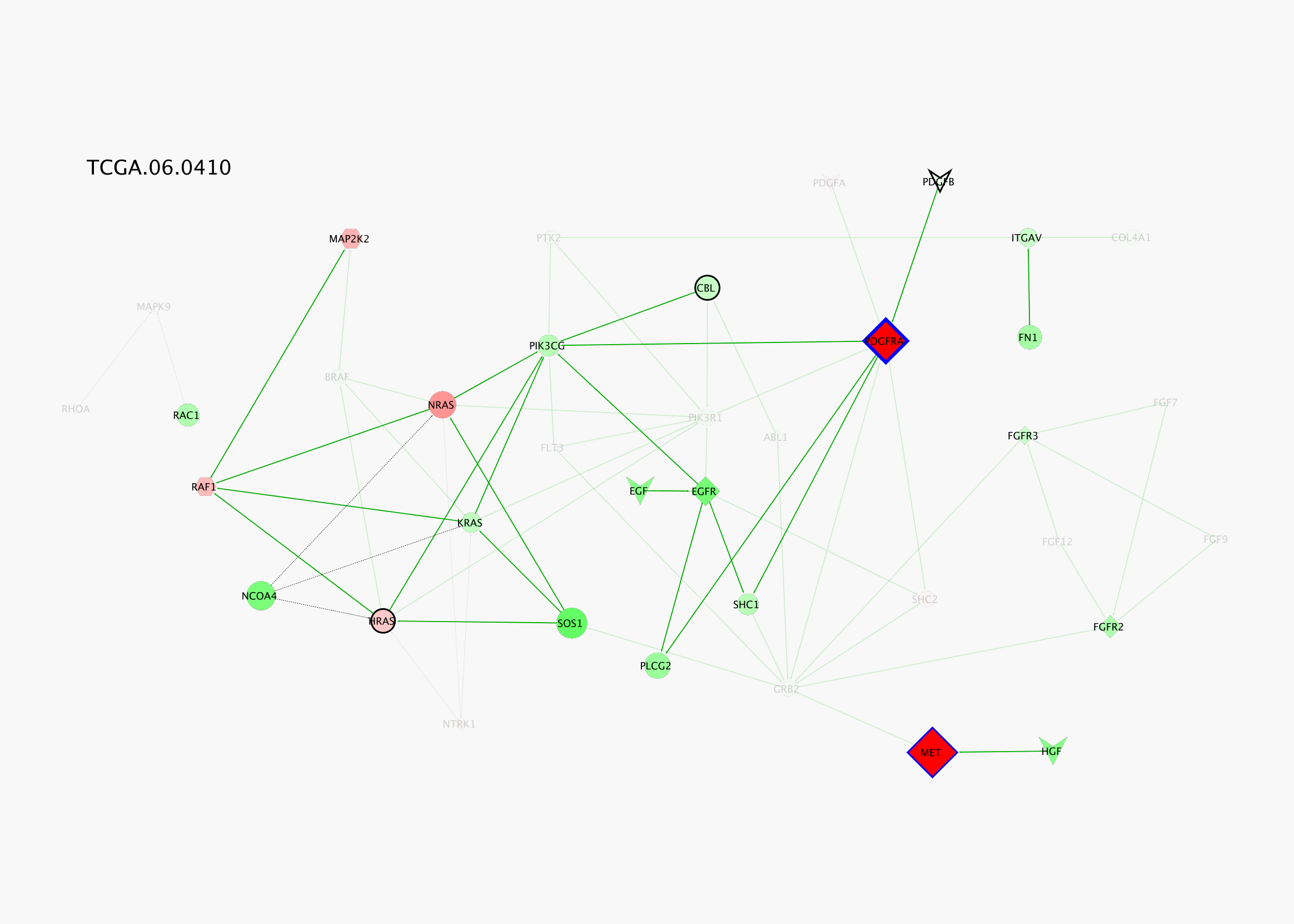

Supplement: Additional file 5 — (Proneural Heterogeneity vignette). [file 1471-2105-14-217-S5.gz › ProneuralHeterogeneity/inst/extdata/TCGA.06.0410.png]

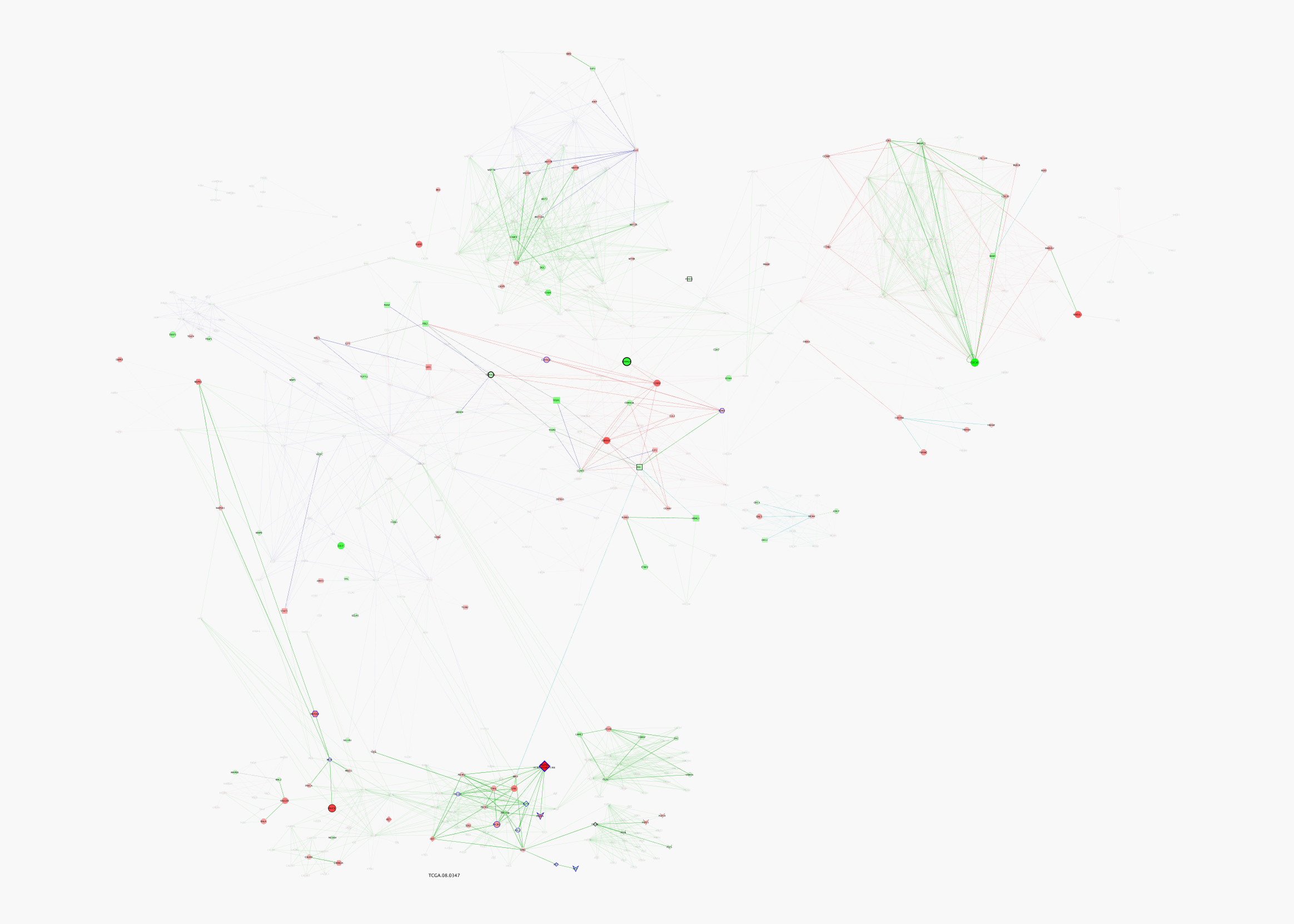

Supplement: Additional file 5 — (Proneural Heterogeneity vignette). [file 1471-2105-14-217-S5.gz › ProneuralHeterogeneity/inst/extdata/TCGA.08.0347-full.png]

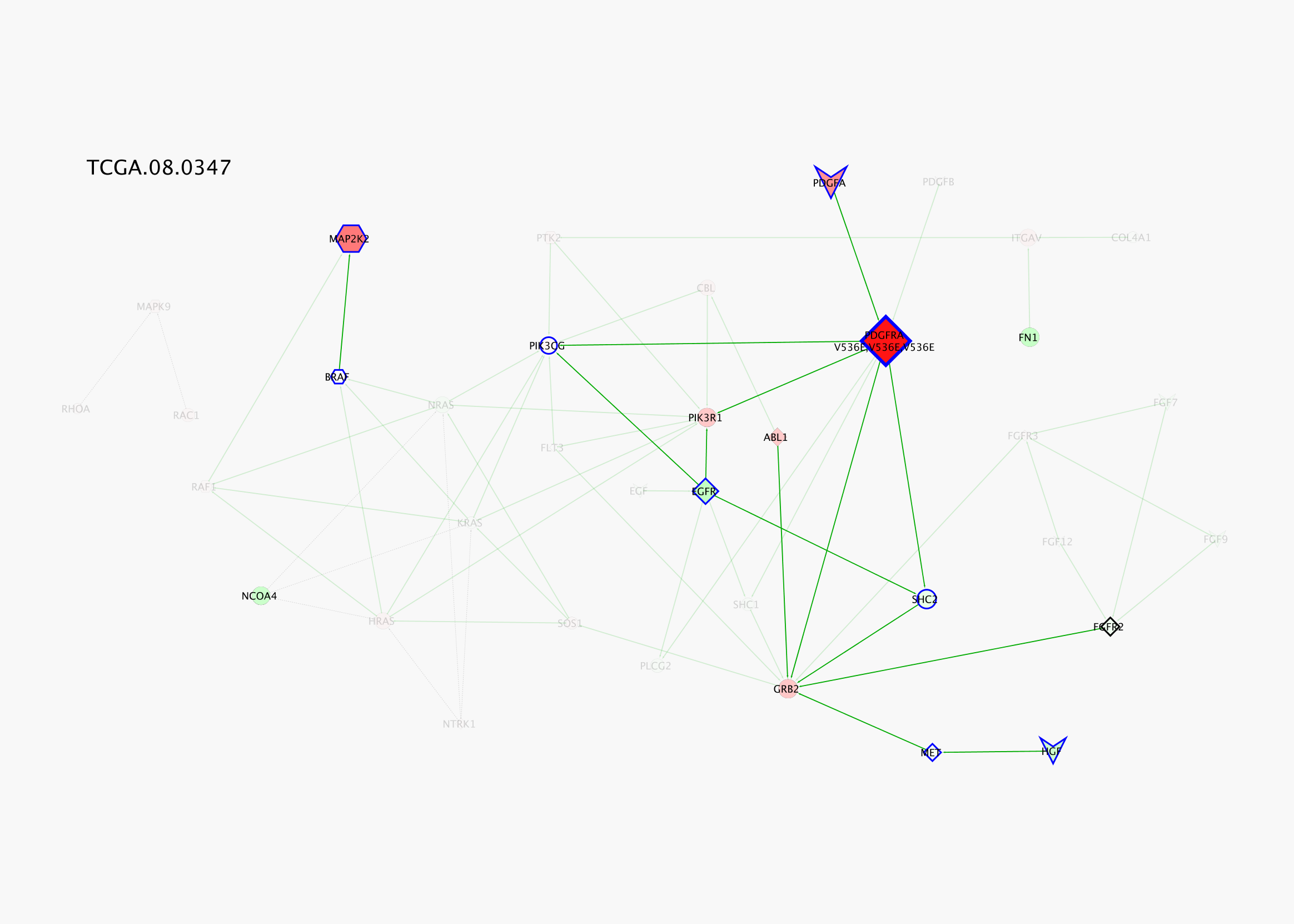

Supplement: Additional file 5 — (Proneural Heterogeneity vignette). [file 1471-2105-14-217-S5.gz › ProneuralHeterogeneity/inst/extdata/TCGA.08.0347.png]

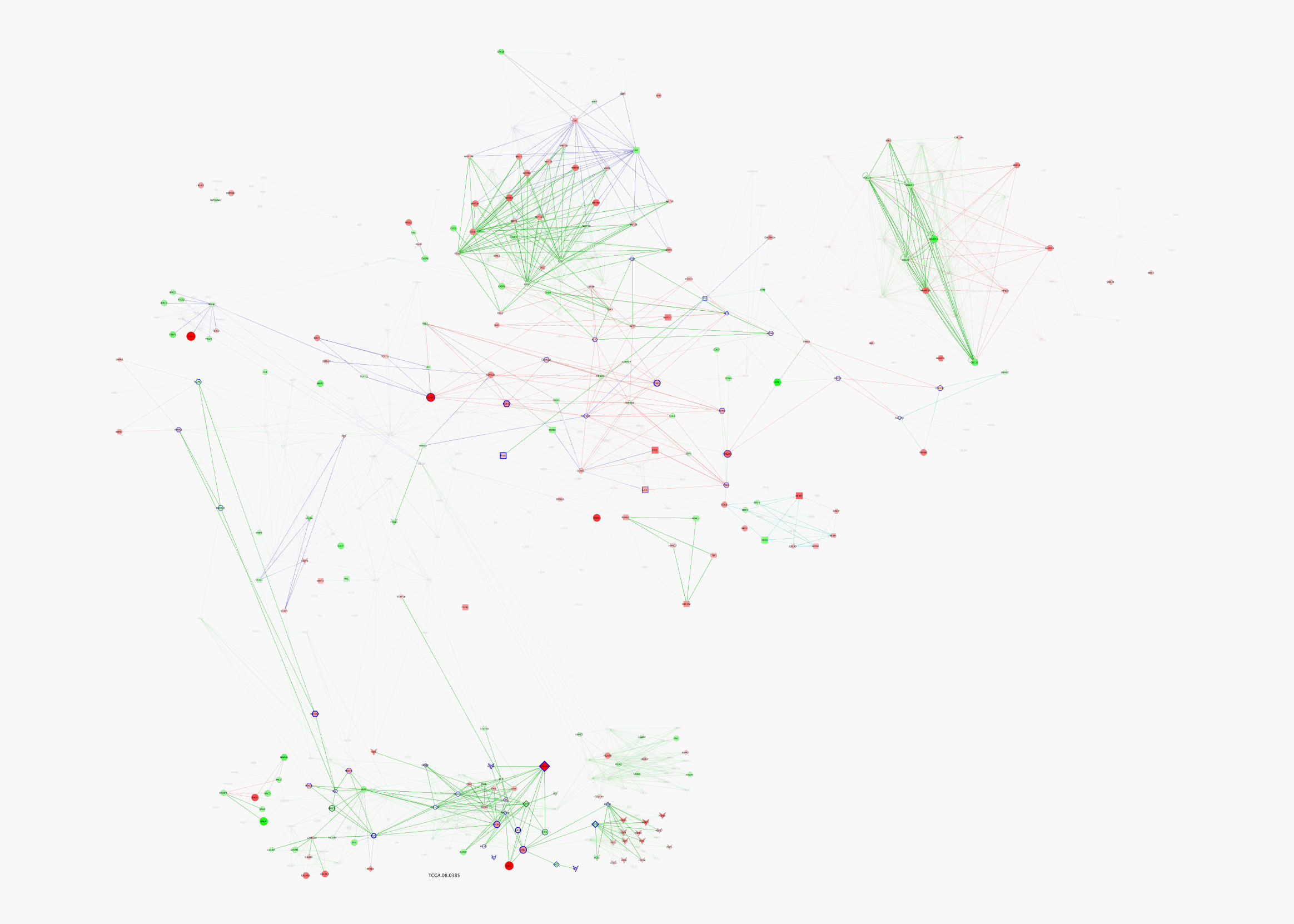

Supplement: Additional file 5 — (Proneural Heterogeneity vignette). [file 1471-2105-14-217-S5.gz › ProneuralHeterogeneity/inst/extdata/TCGA.08.0385-full.png]

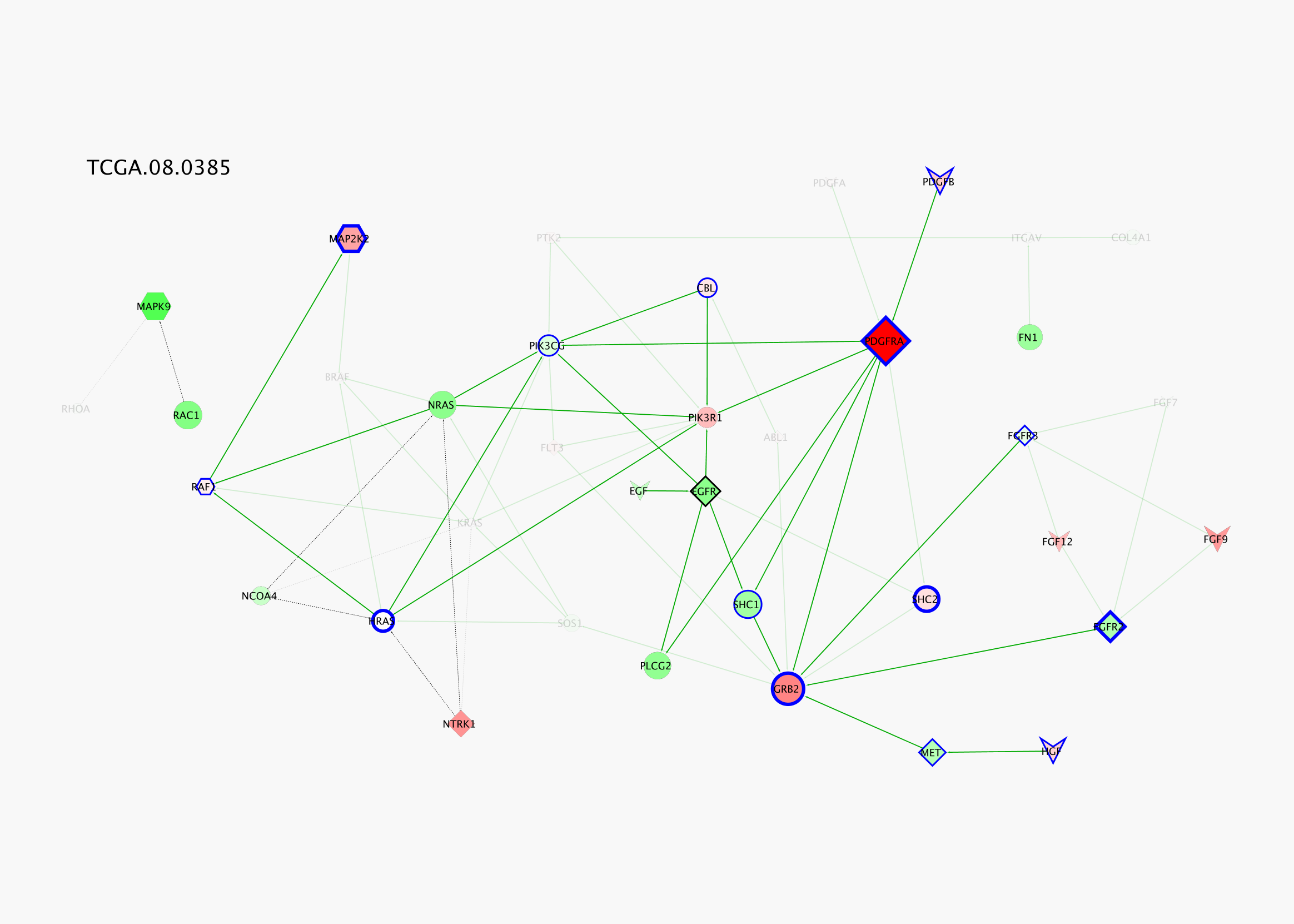

Supplement: Additional file 5 — (Proneural Heterogeneity vignette). [file 1471-2105-14-217-S5.gz › ProneuralHeterogeneity/inst/extdata/TCGA.08.0385.png]

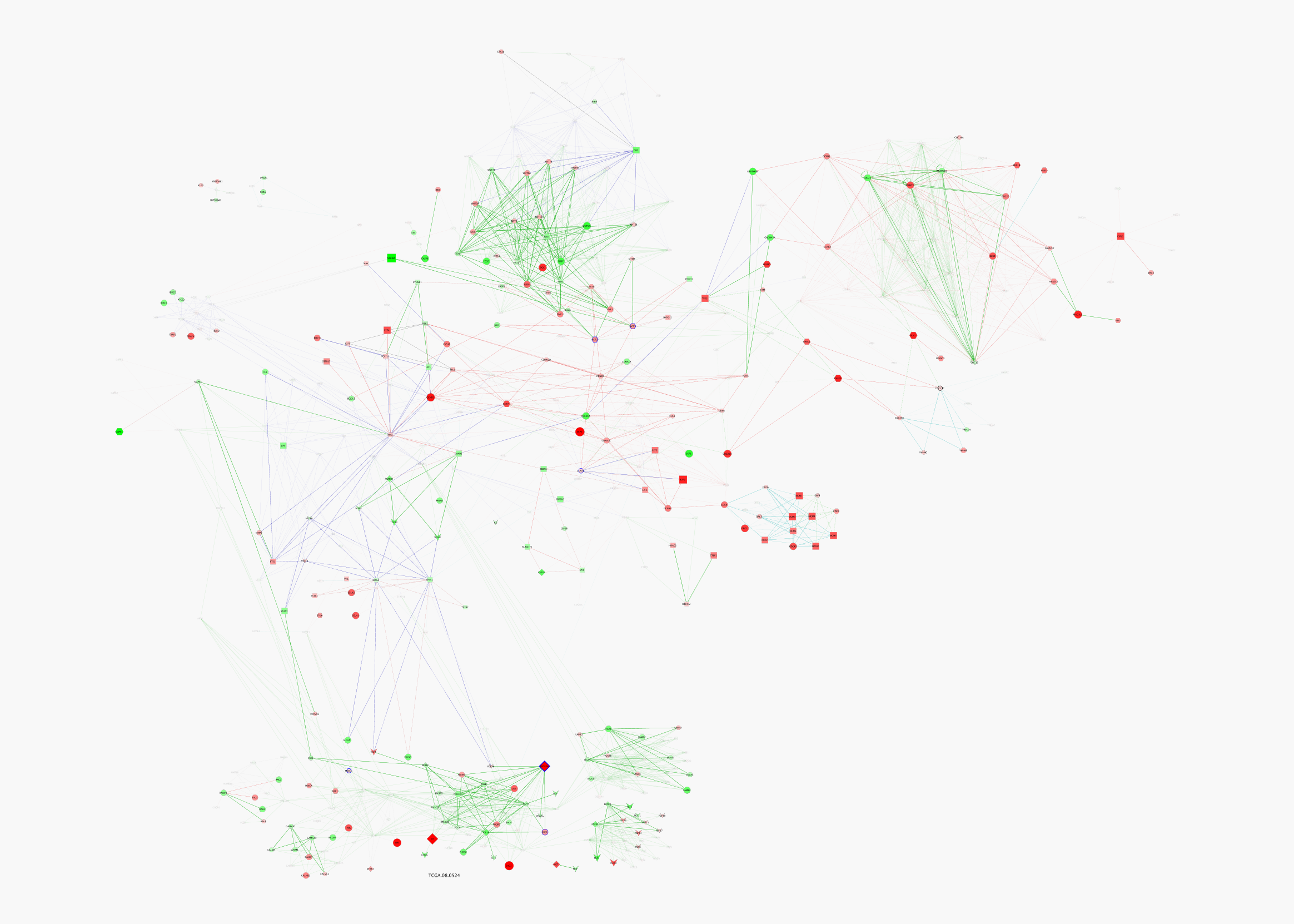

Supplement: Additional file 5 — (Proneural Heterogeneity vignette). [file 1471-2105-14-217-S5.gz › ProneuralHeterogeneity/inst/extdata/TCGA.08.0524-full.png]

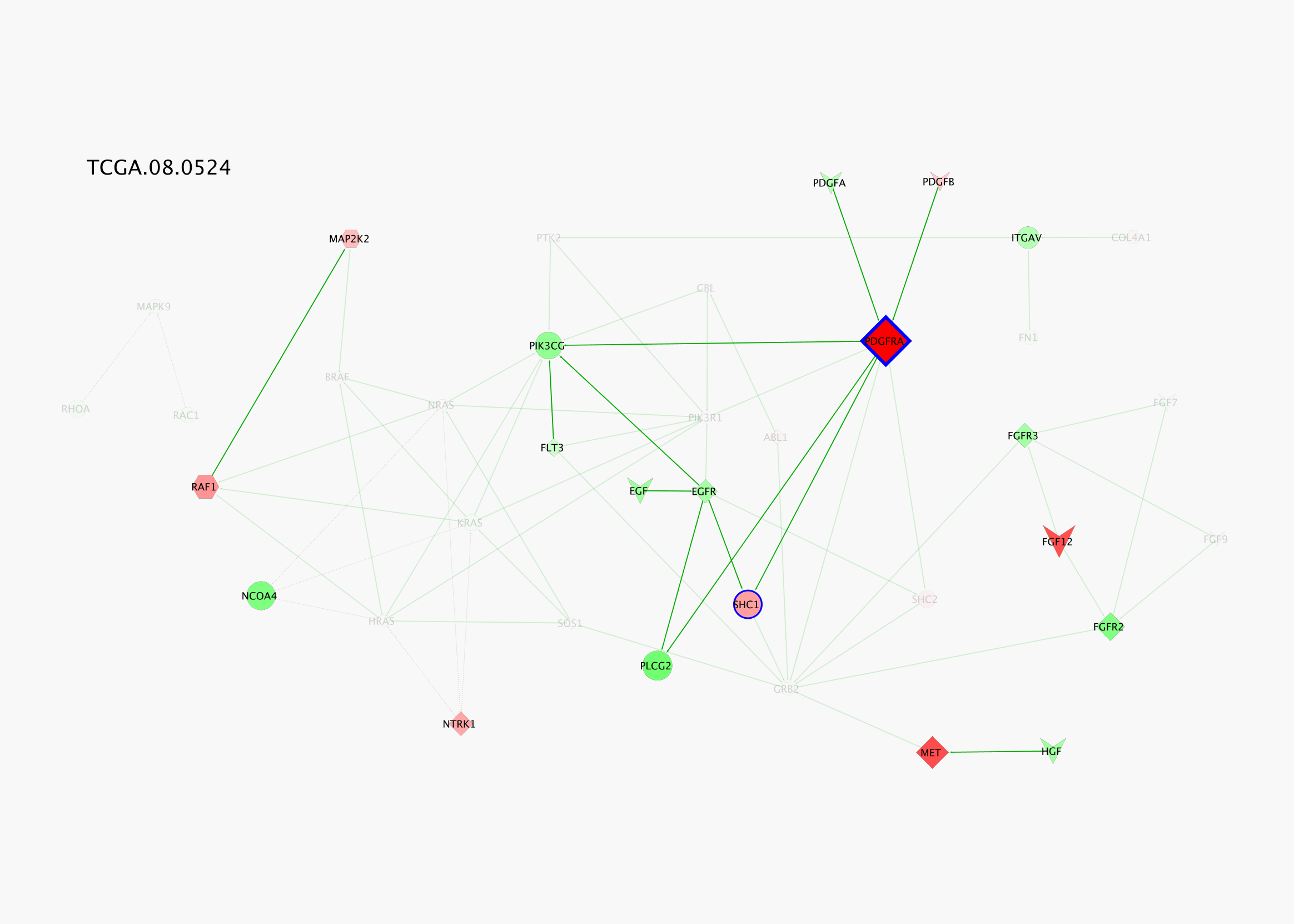

Supplement: Additional file 5 — (Proneural Heterogeneity vignette). [file 1471-2105-14-217-S5.gz › ProneuralHeterogeneity/inst/extdata/TCGA.08.0524.png]
