# Supplementary material for: RCytoscape: tools for exploratory network analysis
Source: BMC Bioinformatics. 2013 Jul 9;14:217. doi: 10.1186/1471-2105-14-217 (PMC3751905; doi:10.1186/1471-2105-14-217)

Proneural Tumors Clustered on Gene Expression
in Three KEGG Cancer−related Pathways

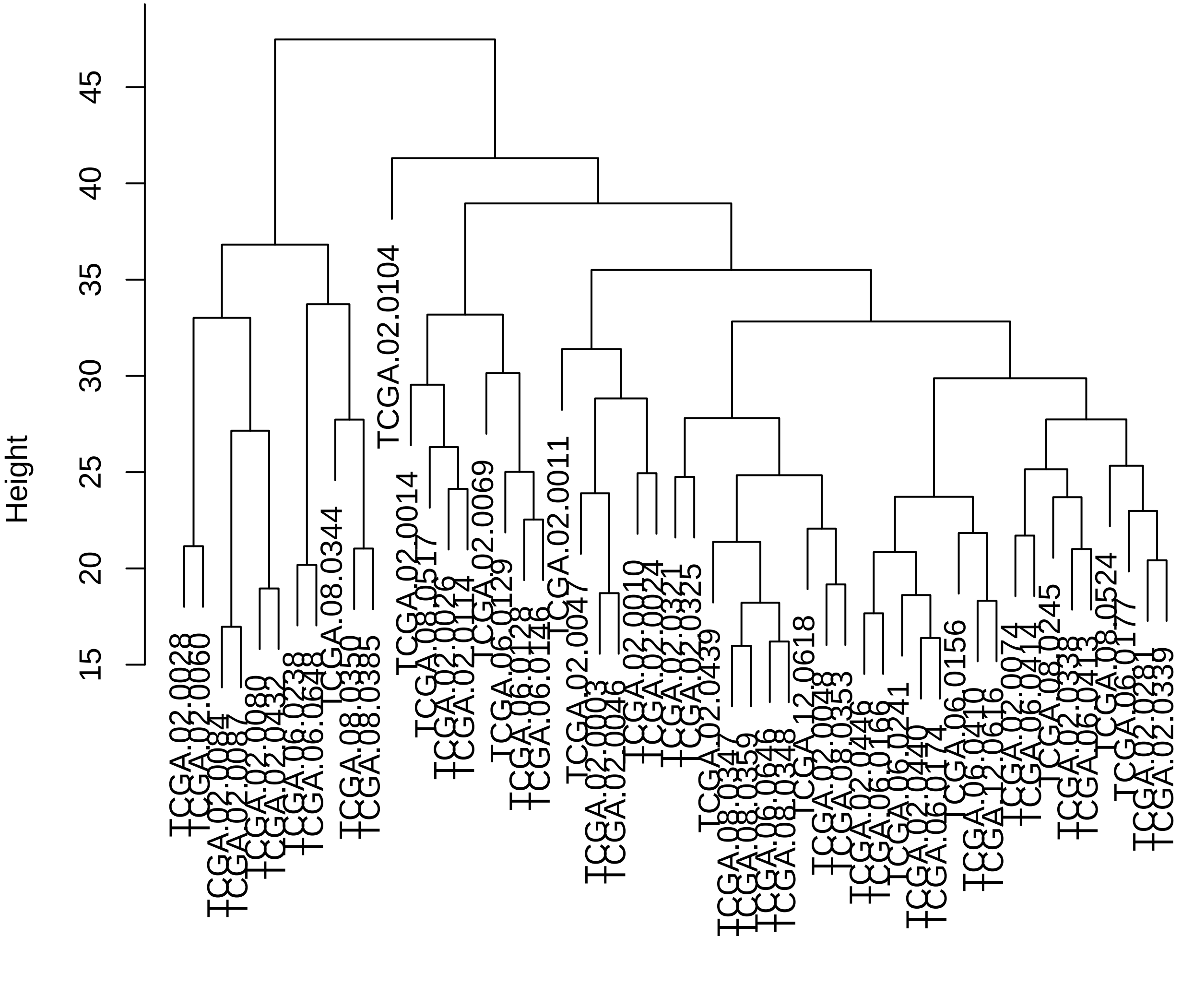

dist(scale(tbl[proneural.tumors,]))
hclust (*, "complete")

Strong Proneural Tumors Clustered on Gene Expression in Three KEGG Pathways

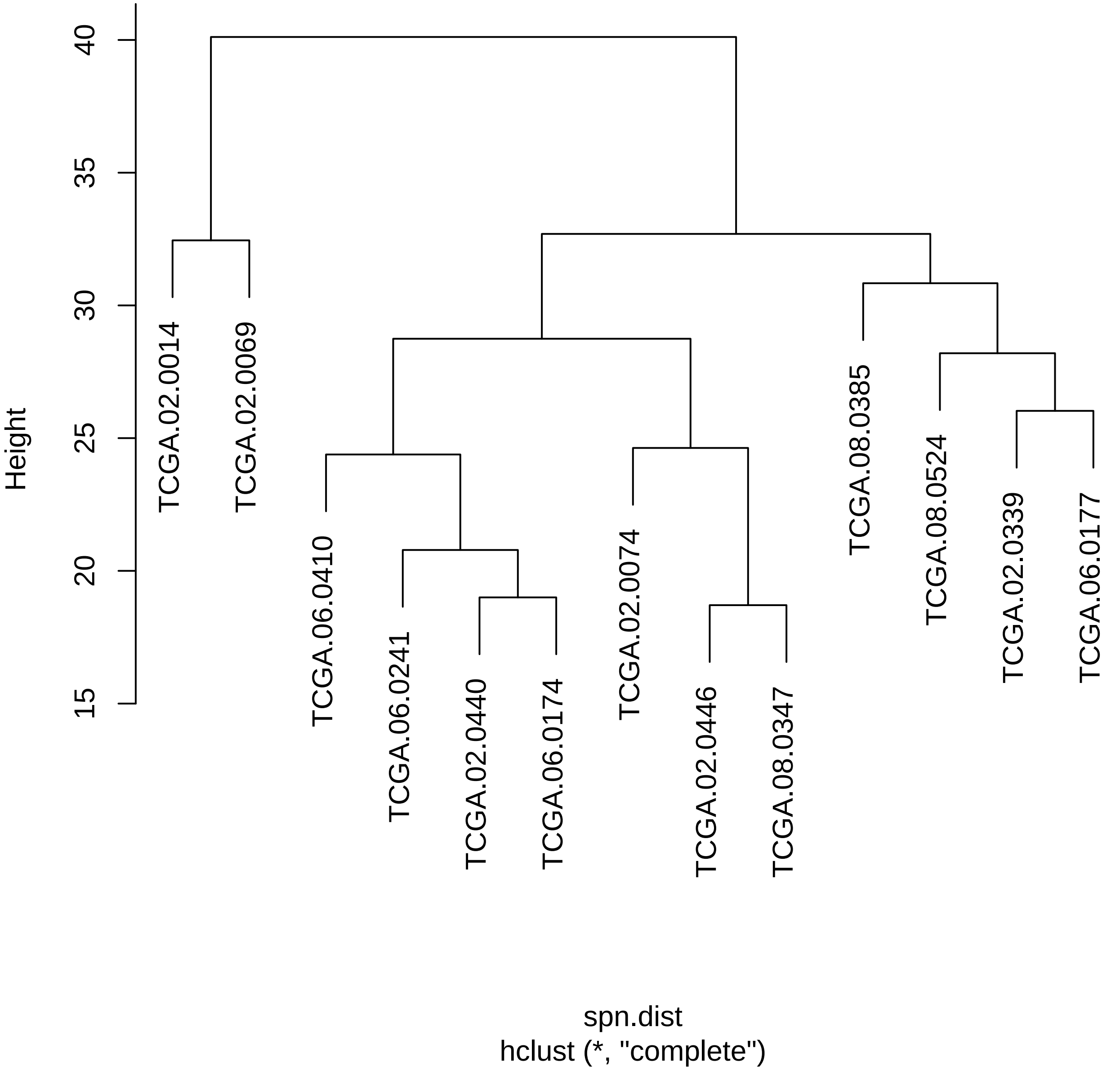

Supplement: Additional file 5 — (Proneural Heterogeneity vignette). [file 1471-2105-14-217-S5.gz › ProneuralHeterogeneity/inst/Rplots.pdf]
