# Supplementary material for: RCytoscape: tools for exploratory network analysis
Source: BMC Bioinformatics. 2013 Jul 9;14:217. doi: 10.1186/1471-2105-14-217 (PMC3751905; doi:10.1186/1471-2105-14-217)
Supplement: Additional file 5 — (Proneural Heterogeneity vignette). [file 1471-2105-14-217-S5.gz › ProneuralHeterogeneity/inst/doc/ProneuralHeterogeneity.pdf]

# The Heterogeneity of TCGA's *Strong Proneural* Tumors

Paul Shannon

March 4, 2013

## Contents

**1 Introduction** **1**

1.1 Background: Verhaak's Classification . . . . . 2

1.2 Background: John Tukey's Exploratory Data Analysis . . . . . 3

1.3 Strategy . . . . . 3

**2 Identify the *Strong Proneural* Tumors and Explore Their Gene Expression Heterogeneity** **4**

2.1 Load the Data . . . . . 4

2.2 Combine Three KEGG Cancer-Related Pathways . . . . . 8

2.3 Choose a Pair of Strong Proneural Tumors for Network Visualization . . . . . 10

**3 Display The Two Selected Strong Proneural Tumors** **14**

**4 Create an RCytoscape Movie of all Strong Proneural Tumors** **16**

# 1 Introduction

This document accompanies and supports the paper *RCytoscape: Tools for Exploratory Network Analysis* [Shannon et al. (2013)]. It provides:

- A summary of relevant portions of the [Verhaak et al. (2010)] Glioblastoma analysis.
- All of the R code needed to reproduce the molecular maps in [Shannon et al. (2013)] of two Glioblastoma multiforme "Strong Proneural" tumors.
- Full code, narrative and details behind the selection of those two tumors from all of those available.
- How to create an animated tour of all thirteen Proneural tumors in a small network, a subset of a larger network merged from three KEGG pathways, centered upon the PDGFRA receptor tyrosine kinase.

## 1.1 Background: Verhaak's Classification

In *Integrated Genomic Analysis Identifies Clinically Relevant Subtypes of Glioblastoma Characterized by Abnormalities in PDGFRA, IDH1, EGFR, and NF1* [Verhaak et al. (2010)], propose a

> "robust gene expression-based molecular classification of GBM into Proneural, Neural, Classical, and Mesenchymal subtypes [based upon their integration of] multidimensional genomic data to establish patterns of somatic mutations and DNA copy number ... [revealing] a strong relationship between subtypes and different neural lineages."

Current clinical practice uses such robust molecular classification of tumors with some success to choose effective therapies. With Glioblastoma multiforme (GBM), such treatments are sorely needed: GBM is the most aggressive and most common of brain cancers; the median patient survival is one year. Verhaak et al describe four categories of GBM tumors (Proneural, Neural, Classical, and Mesenchymal), each with a molecular signature, each from a different neural lineage, and note that response to treatement differs by category:

> "Temozolomide and radiation, a common treatment for glioblastoma, has demonstrated a significant increase in survival. Our analysis illustrates that a survival advantage in heavily treated patients varies by subtype, with Classical or Mesenchymal subtypes having significantly delayed mortality that was not observed in the Proneural subtype."

They describe their method:

> "Factor analysis, a robust method to reduce dimensionality, was used to integrate data from 200 GBM and two normal brain samples assayed on three gene expression platforms (Affymetrix HuEx array, Affymetrix U133A array, and Agilent 244K array) into a single, unified data set. Using the unified data set, we filtered the data to 1740 genes with consistent but highly variable expression across the platforms. Consensus average linkage hierarchical clustering [Monti et al. (2003)] of 202 samples and 1740 genes identified four robust clusters."

And this result:

> "[t]wo major features of the Proneural class were alterations of PDGFRA and point mutations in IDH1. Focal amplifications of the locus at 4q12 harboring PDGFRA were seen in all subtypes of GBM but at a much higher rate in Proneural samples($p = 0.01$, adjusted two-sided Fisher's exact test, ...). The characteristic signature of PDGFRA in Proneural samples, however, is best described as the concomitant focal amplification in conjunction with high levels of PDGFRA gene expression, which is seen almost exclusively in this tumor type ($p < 0.01$, two-sided Student's t test...). Four of the Proneural samples amplifying PDGFRA also harbor a PDGFRA mutation."

This figure[Verhaak et al. (2010)] indicates the subset of Proneural tumors from which the thirteen "Strong Proneural" tumors have been drawn.

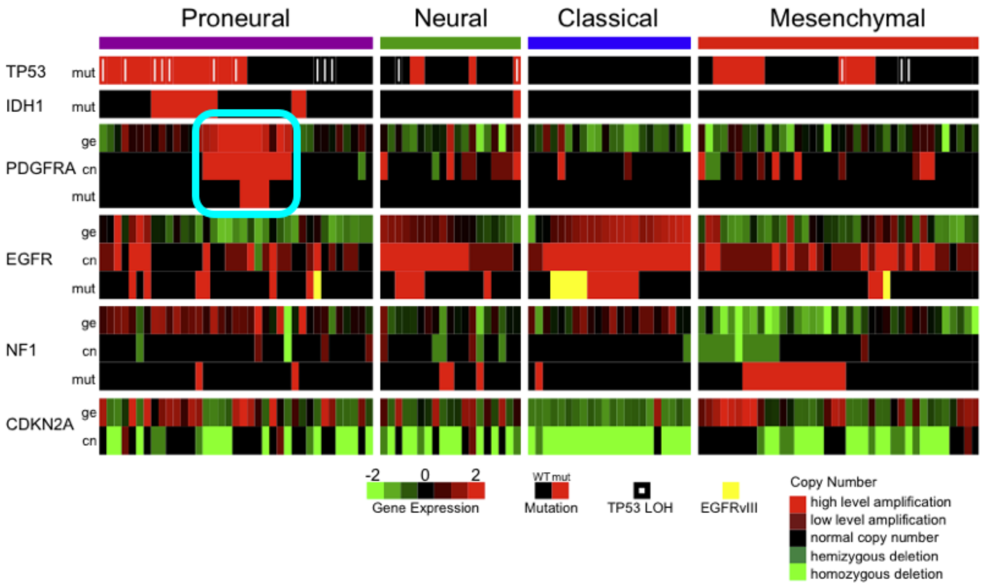


## 1.2 Background: John Tukey's Exploratory Data Analysis

The resistance of Proneural tumors to treatment motivates this case study. We approach the question with a bias towards synoptic analysis, and the expectation that more often than not, biological explanations will not be simple. We explore the hypothesis that amplification and over-expression of PDGFRA may be a necessary, but not sufficient, explanation of Pronueral tumors. We employ exploratory data analysis, in the style of John Tukey [Tukey (1977)], and using *R* and *RCytoscape* for detailed network visualization. We focus on one subgroup of the treatement-resistant Proneural tumors: those which best fit the amplification/expression signature described above.

Tukey's position, summarized in his own words:

> "Numerical quantities focus on expected values, graphical summaries on unexpected values."

> "[Visualization methods] ... are there, not as a technique, but rather as recognition that the picture-examining eye is the best finder we have of the wholly unanticipated."

## 1.3 Strategy

We proceed in three stages. Our first task is to assess the heterogeneity of the 53 tumors Verhaak classifies as Proneural, which we will accomplish by examining their gene expression across a set of cancer-related genes. We turn to KEGG for this set of genes, while acknowledging that KEGG's curated pathways will include some false (or insufficiently detailed) claims, and will omit some genes and interactions which bear upon glioblastoma. The many well-known and tumorigenic-related genes and interactions KEGG does provide are however adequate for a first round of exploratory data analysis.

Next, we focus upon the thirteen "Strong Proneural" tumors, that is, those which exhibit Verhaak's "concomitant focal amplification in conjunction with high levels of PDGFRA gene expression", and explore their gene expression

heterogeneity across the same KEGG genes. This sets the stage for selecting two tumors for closer examination, as reported in [Shannon et al. (2013)], and providing the code which reproduces those maps.

Third, we conclude by presenting a RCytoscape animations. The same network map is rendered in sequence, a frame at a time, using a different tumor's data for each frame. This animation illustrates the means by which an extra dimension of information can be added to a network display. Here, the extra dimenion is tumor heterogeneity. Another application of this RCytoscape technique, with time-series data, in which the extra dimenion is cell state across time, promises to yield rich insights into molecular processes.

# 2 Identify the *Strong Proneural* Tumors and Explore Their Gene Expression Heterogeneity

We begin by examining the fifty-three tumors previously identified as Proneural (for this classification, see below). Thirteen of these are exemplary in that they have both high mRNA expression and strong amplification of PDGFRA. Inasmuch as these thirteen tumors are the best fit to the Proneural signature, discovering heterogeneity here should be of greater biological interest than if it were found among the full set of fifty-three, which includes tumors less well matched to the signature.

The analysis contains these steps:

- Load the data: annotation, TCGA experimental data, and KEGG cancer-related networks.
- Identify the tumors which meet the *strong Proneural* PDGFRA mRNA and amplification signature.
- Construct a data.frame from three KEGG cancer-related pathways.
- Calculate and visualize the Euclidean distance between the 13 tumors with respect to genes in the combined KEGG pathways.
- Select, from among these 13, the tumor pair exhibiting the largest dissimilarity.

The skeptical reader may wonder if, by choosing the most dissimilar tumor pair from among the thirteen strong Proneurals, the subsequent argument for heterogeneity is compromised. To allay those concerns, we include below an analysis of gene expression heterogeneity for all possible pairs among those thirteen tumors, establishing that the dissimilarity of the chose tumor pair is not unusual, and thus that these two may reasonbably, vividly, yet without distortion illustrate the kinds of heterogeneity found in the strong Proneural group as a whole.

## 2.1 Load the Data

Here we load the packages and data we need:

- *ProneuralHeterogenity*: includes all the code, convenience functions, and data needed to run this analysis. Three tables of experimental data were previously downloaded from the TCGA, are included here.
- *org.Hs.eg.db*: The Bioconductor genome-wide annotation for Human, used here to interconvert gene symbols (used by TCGA) and geneIDs (used by KEGG and Bioconductor annotation resources).
- Define a convenience function *printf*.

- tbl.mrna: Z-scores of gene expression, tumor by tumor, with four normal brain tissue samples for comparision, downloaded in Feburary 2012. Though these data are available quite reliably in real-time from cdgsr, reproducability leads us to include it here.
- tbl.cnv: Genomic copy number for each gene in every tumor. The qualitative scale is explained below.
- tbl.mut: Mutations, expressed as mino acid substitutions
- geneTypes: an informal collection of gene category assignements we created for this project, extracted selectively from GO: TF, kinase, growth factor, receptor, and gene (for otherwise uncategorized genes)

```
> options(width=80)
```

```
> library(ProneuralHeterogeneity)
> library(org.Hs.eg.db)
> printf = function(...) print(noquote(sprintf(...)))
> data(tbl.mrna)
> data(tbl.cnv)
> data(tbl.mut)
> data(tbl.subtypes)
> data(geneTypes)
```

Let us examine each of these data objects. The gene expression data.frame, *tbl.mrna* allows us to understand the provenance of these data. Our analysis here is applied to data describing 201 GBM tumors released by the TCGA in the summer of 2011. At this point in our project, we obtained all TCGA experimental data live and online each time we reran the analysis, from the Memorial Sloan-Kettering Cancer Center cBio Cancer Genomics Portal's R package *cgdsr* (see `http://cran.r-project.org/web/packages/cgdsr`).

In the autumm of 2011, the TCGA added to the GBM tumor data, increasing the total tumor count to 577. We have continued to focus on the original 201 tumors. A standard technique ofr comparing mRNA expression levels across many experiments (in this case, across many tumors). gene expression Z-scores [**?**] a statistic which takes into account all of the gene expression arrays under consideration. When the TCGA expanded the publicly available GBM tumor data from 201 tumors to 577, the gene-expression Z-scores of our original Pronueral tumors changed. At that point we decided to download and save the Z-scores based on the larger tumor set; these mRNA expression values, current as of January 2012, and accompanied by mutation, copy number and categorization data, are contained in and used by this package.

Identify the tumors classified as Proneural by Verhaak et al:

```
> proneural.tumors.raw <- colnames(tbl.subtypes) [which(tbl.subtypes [1,] == 'Proneural')]
> proneural.tumors <- gsub('.01[ABC].0[123]', '', proneural.tumors.raw)
```

In order to identify the subset which best fit the class signature (high expression, high copy number) we must first understand the qualitative convention TCGA used to describe genomic amplification. The distinction between 'gain' and 'amplification' is not entirely clear, but seems to be a matter of degree. We opt for the stronger attribute in the filtering which follows.

- -2 Homo deletion
- -1 Hetero deletion

- 0 Diploid
- 1 Gain
- 2 Amplification

Use this scheme and gene expression to identify the tumors which fit the *strong proneural* signature:

```
> PDGFRA.geneID = '5156'
> pdgfra.highly.expressed.tumors <-
+      rownames(tbl.mrna) [which(tbl.mrna [, PDGFRA.geneID] >= 2)]
> pdgfra.amplified.tumors <-
+      rownames(tbl.cnv) [which(tbl.cnv [, PDGFRA.geneID] == 2)]
> proneural.tumors.strong <-
+     intersect(proneural.tumors, intersect(pdgfra.highly.expressed.tumors,
+                                          pdgfra.amplified.tumors))
> proneural.tumors.strong
```

```
 [1] "TCGA.02.0014" "TCGA.02.0069" "TCGA.02.0074" "TCGA.02.0339" "TCGA.02.0440"
 [6] "TCGA.02.0446" "TCGA.06.0174" "TCGA.06.0177" "TCGA.06.0241" "TCGA.06.0410"
[11] "TCGA.08.0347" "TCGA.08.0385" "TCGA.08.0524"
```

Examine the distribution of gene expression Z-scores:

```
> pdgfra.expression.scores <- tbl.mrna [, PDGFRA.geneID]
> pdgfra.expression.in.proneurals <- tbl.mrna[proneural.tumors, PDGFRA.geneID]
> pdgfra.expression.in.strong.proneurals <- tbl.mrna[proneural.tumors.strong, PDGFRA.geneID]
>    # establish bottom, left, top, right margins for the boxplot
> par(oma=c(1,0,1,0))
> boxplot(pdgfra.expression.scores,
+           pdgfra.expression.in.proneurals,
+           pdgfra.expression.in.strong.proneurals,
+           names=c("All", 'Proneural', 'Proneural-Strong'),
+           main="PDGFRA Z-score log fold mRNA expression", xlab="Tumors")
```

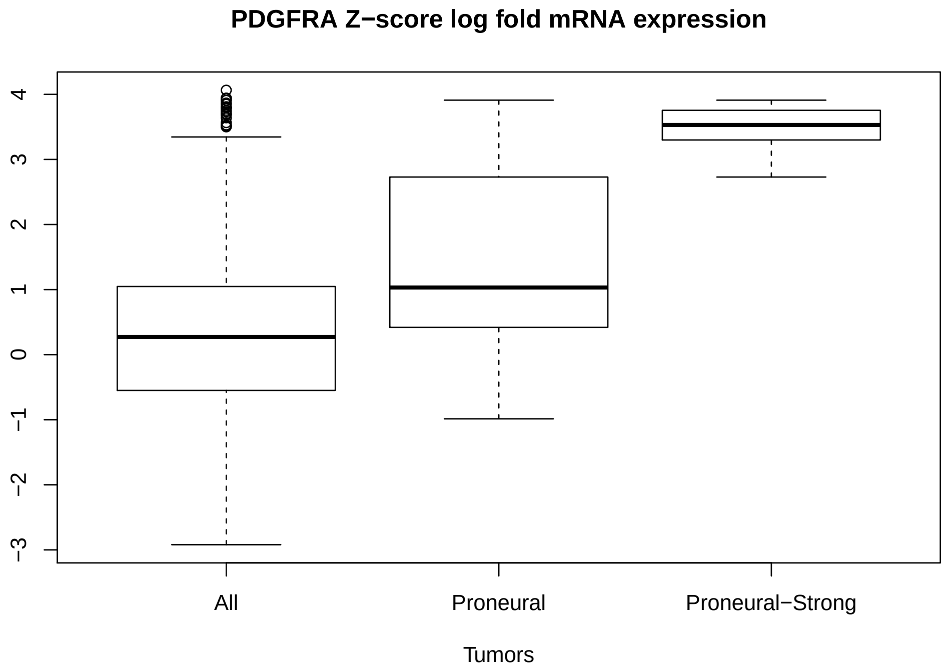


Learn the distribution of these alterations for PDGFRA across the full 204 tumors:

```
> pdgfra.cnv.scores <- tbl.cnv [, PDGFRA.geneID]
> pdgfra.cnv.in.proneurals <- tbl.cnv[proneural.tumors, PDGFRA.geneID]
> pdgfra.cnv.in.strong.proneurals <- tbl.cnv[proneural.tumors.strong, PDGFRA.geneID]
> xtab.all = table(pdgfra.cnv.scores)
> xtab.proneurals = table(pdgfra.cnv.in.proneurals)
> xtab.strong = table(pdgfra.cnv.in.strong.proneurals)
> xtab.combined <- matrix(0, nrow=3, ncol=5, dimnames=list(c("allTumors", 'proneuralTumors', "strongPr
> xtab.combined["allTumors", names(xtab.all)] = as.integer(xtab.all)
> xtab.combined["proneuralTumors", names(xtab.proneurals)] = as.integer(xtab.proneurals)
> xtab.combined["strongProneuralTumors", names(xtab.strong)] = as.integer(xtab.strong)
> colnames(xtab.combined) <- c("Homo deletion", "Hetero deletion", "Diploid", "Gain", "Amplification")
> print(xtab.combined)
```

```
                      Homo deletion Hetero deletion Diploid Gain Amplification
allTumors                         0               4     168   12            22
proneuralTumors                   0               1      34    2            15
strongProneuralTumors             0               0       0    0            13
```

We note in passing that of the 206 tumors from the larger TCGA GBM data release for which copy number is reported, none have suffered a homozygous deletion: PDGFRA is apparently present in at least one copy in all tumors. It is evident from these two summaries, PDGFRA gene expression and copy number, that the "strong proneural" tumors, when analyzed with respect to PDGFRA, is a well-defined group.

## 2.2 Combine Three KEGG Cancer-Related Pathways

We now prepare to add a network view of the data. KEGG provides us with annotated cancer-related pathways, of which we selected three for download using the Bioconductor *KEGGgraph* [Zhang and Wiemann (2009)] package. We will use these combined pathways to create a Cytoscape network upon which to display the GBM tumor data, and to create a gene list which, when intersected with the genes measured by TCGA, will allow us to determine the Euclidean distance between all strong proneural tumor pairs, as promised above.

The pathways we selected:

- Glioma (hsa05214)
- Cell Cycle (hsa04110)
- Pathways in Cancer (hsa05200)

Additional KEGG pathways could be included here: Wnt Signaling, for instance, and the MAP kinase pathway. Pathways from from REACTOME could be included as well. Exploratory data analysis, however, does not require us to begin our survey with a comprehensive molecular map. We propose that these three KEGG pathways, are adequate to start with, and that further pathways, or more accurate pathways (as well as additional experimental and annotation data) could be added in subsequent analyses, complementing or replacing those used here, and selected in response to this initial exploration.

Create a data.frame based upon downloads from of three cancer-related pathways from KEGG, using the *KEGGgraph* package. We will display this "full network" in Cytoscape. Later, we zoom into a considerably smaller subnework restricted to the neighborhood of PDGFRA, over which we will display tumor-specific experimental data. For now, we want the full view of these three combined networks.

Note that here, and later in this vignette, we provide and call several convenience functions that are provided by this, the *PronerualHeterogeneity* package. The full text of these functions is displayed at the conclusion of this vignette, and the R code can be extracted from the package itself. Both of these options may be of interest to those who wish to adopt and adapt these functions to other projects.

The function `tumorViz`, however will be of immediate interest. It contains the vizmap rules which control, among other things, the colors used for different interactions (edges) in the network. Let us look at one object in particular defined there, `edge.map`, a list of lists.

```
edge.map <-  list(
"activation"                 = list(line="SOLID",       targetArrow="Arrow",    sourceArrow="No Arrow", color="#00AA00", width=6),
"activation;phosphorylation" = list(line="SINEWAVE",    targetArrow="Arrow",    sourceArrow="No Arrow", color="#00AA00", width=3),
"binding/association"        = list(line="SOLID",       targetArrow="Arrow",    sourceArrow="Arrow",    color="#00AAAA", width=3),
"inhibition;phosphorylation" = list(line="SINEWAVE",    targetArrow="T",        sourceArrow="No Arrow", color="#DD0000", width=3),
"inhibition"                 = list(line="DASH_DOT",    targetArrow="T",        sourceArrow="No Arrow", color="#DD0000", width=3),
"ubiquination"               = list(line="SOLID",       targetArrow="Arrow",    sourceArrow="No Arrow", color="#DD00DD", width=3),
"repression"                 = list(line="SOLID",       targetArrow="T",        sourceArrow="No Arrow", color="#DD0000", width=3),
"dissociation"               = list(line="DASH_DOT",    targetArrow="No Arrow", sourceArrow="No Arrow", color="#000000", width=3),
"indirect effect"            = list(line="EQUAL_DASH",  targetArrow="Arrow",    sourceArrow="No Arrow", color="#000000", width=3),
"missing interaction"        = list(line="DOT",         targetArrow="No Arrow", sourceArrow="No Arrow", color="#AA00AA", width=3),
"expression"                 = list(line="SOLID",       targetArrow="Delta",    sourceArrow="No Arrow", color="#0000AA", width=3),
"missing interaction"        = list(line="DOT",         targetArrow="No Arrow", sourceArrow="No Arrow", color="#AA0000", width=3),
"dimerize"                   = list(line="DOT",         targetArrow="No Arrow", sourceArrow="No Arrow", color="#00AA00", width=3),
"phosphorylation"            = list(line="SINEWAVE",    targetArrow="Arrow",    sourceArrow="No Arrow", color="#00AA00", width=3),
"dephosphorylation"          = list(line="DOT",         targetArrow="Arrow",    sourceArrow="No Arrow", color="#00AA00", width=3),
"compound"                   = list(line="DOT",         targetArrow="No Arrow", sourceArrow="No Arrow", color="#000000", width=3),
"unspecified"                = list(line="DOT",         targetArrow="No Arrow", sourceArrow="No Arrow", color="#000000", width=3),
"functional"                 = list(line="DOT",         targetArrow="No Arrow", sourceArrow="No Arrow", color="#000000", width=3)
)
```

Notice Many other rules are found in this function, and will be worthy of study if you wish to create your own vizmap rules.

Now, to display the full network:

```
> tbl.kegg = combineKeggPathways(c('05214', '04110', '05200'), organism='hsa')
> kegg.geneIDs <- unique(c(tbl.kegg$from, tbl.kegg$to))
> layout.file <- system.file("extdata", "layoutFull.RData",
+                              package="ProneuralHeterogeneity")
> cw <- displayNetwork(tbl.kegg, layout.file, geneTypes, genes.of.interest=NULL)
```

Manipulate some aspects of the Cytoscape GUI:

setWindowSize(cw, 800, 600) hideAllPanels(cw) showGraphicsDetails(cw, TRUE) tumorViz(cw, tumor.name=NULL) fitContent(cw) setZoom(cw, 0.9 * getZoom(cw))

We now ask Cytoscape to save this map. The resulting image is displayed below, after being copied from a temporary directory into this package.

```
> imageFile <- file.path(tempdir(), "fullNetwork.png")
> saveImage(cw, imageFile, image.type="png", scale=2)
> printf("saving image to %s", imageFile)
```

```
[1] saving image to /var/folders/sq/k3wp6zzn2bn86jdr1z_ckd8r0000gr/T//RtmpJvIRdg/fullNetwork.png
```

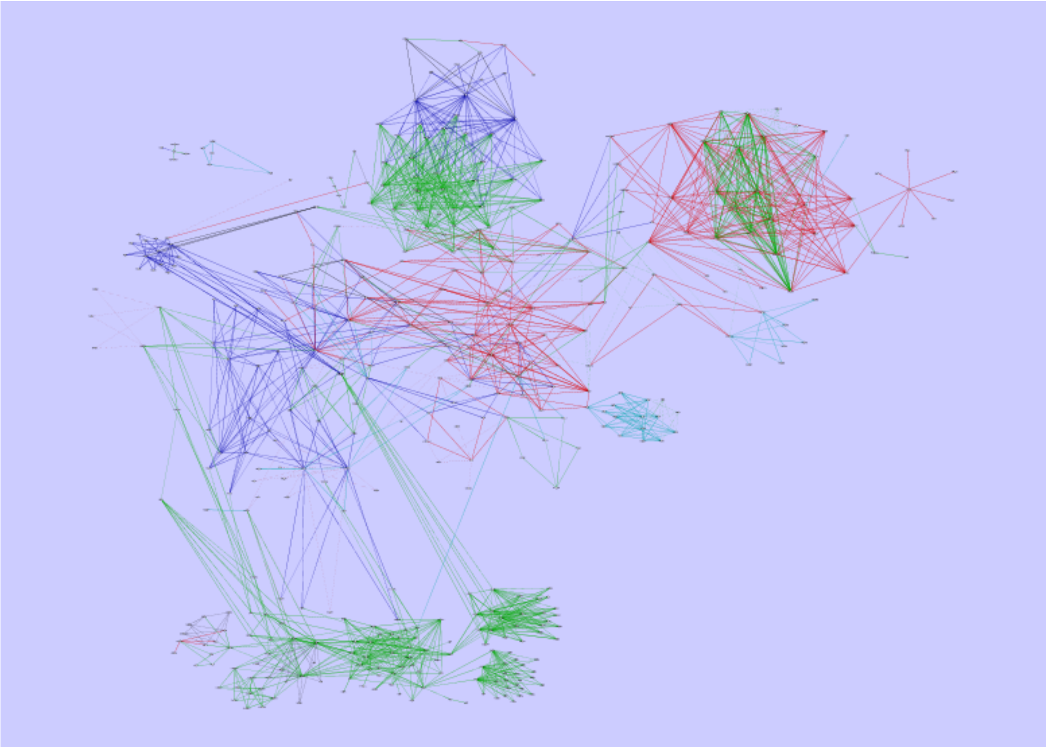

This Cytoscape map is large, with 414 nodes and 1767 edges. We will zoom in on a region of the network to see more detail

```
> selectNodes(cw, PDGFRA.geneID)
> selectFirstNeighborsOfSelectedNodes(cw)
> getSelectedNodeCount(cw)
```

```
[1] 18
```

```
> fitSelectedContent(cw)
> clearSelection(cw)
> setZoom(cw, 0.8 * getZoom(cw))
```

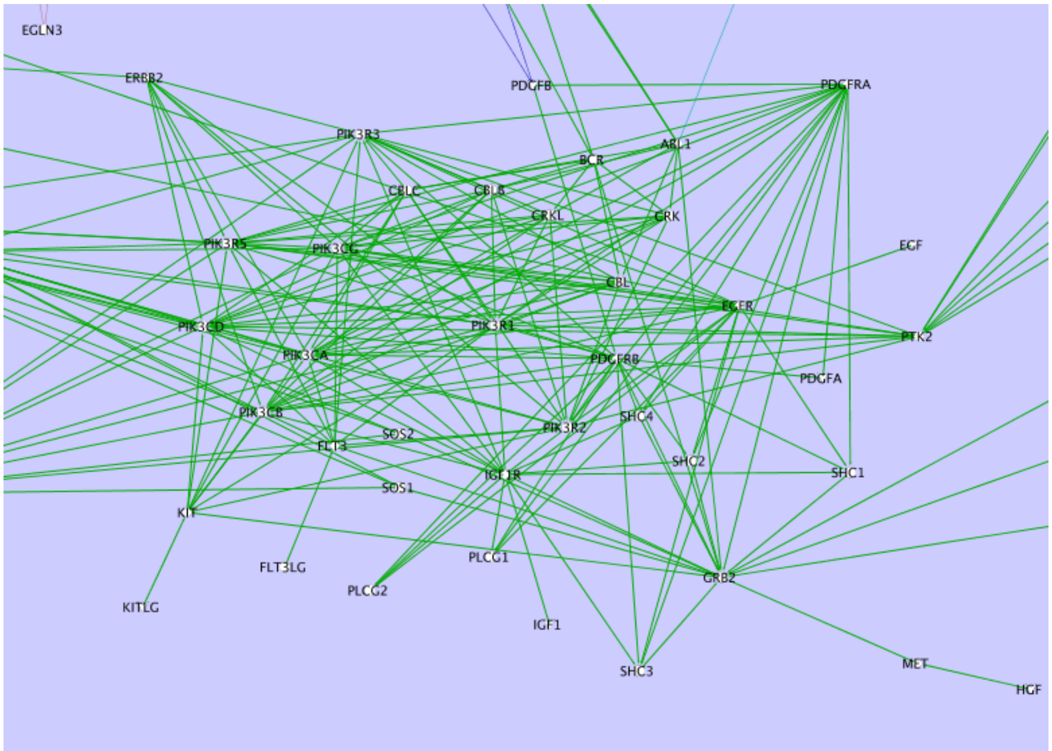


The result is still not very interesting: most of the edges represent activation, and with no experimental data yet added, all of the gene/protein nodes are small and uncolored. We will see more interesting displays below.

## 2.3 Choose a Pair of Strong Proneural Tumors for Network Visualization

Now we look for the two most dissimilar tumors in the 13 member strong proneural group via hierarchical clustering on gene expression. Recall that this group is characterized by essentially identical high PDGFRA expression and amplification. We now turn to an examination of their heterogeneity, choosing the pair of tumors which are the most distant, when the thirteen tumors are hierarchically clustered by mRNA expression across 410 genes. In order to establish that this tumor pair are not anomalies within the group, we calculate the pairwise correlation of all tumor pairs in the group. As will become clear, the selected pair (TCGA.02.0014 and TCGA.08.0385) are sufficently low in correlation to provide an interesting case study, but not so low as to be unrepresentative of the strong proneural group as a whole

We first perform hierarchical clustering, plot the result, and determine the tumor pair with the greatest Euclidean between them.

```
> common.geneIDs <- intersect(kegg.geneIDs, colnames(tbl.mrna))
> tbl <- as.matrix(tbl.mrna [proneural.tumors.strong, common.geneIDs])
> distance.table <- dist(scale(tbl))
> title.1 <- "Hierarchical Clustering of Strong Proneural Tumors"
> title.2 <- "Based on mRNA Expression of 410 KEGG Cancer Genes"
> title <- paste(title.1, title.2, sep="\n")
> plot(hclust(distance.table), main=title, xlab="")
> distance.matrix <- as.matrix(distance.table)
> distance.vector <- as.numeric(distance.matrix * upper.tri(distance.matrix))
> max.distance <- max(distance.vector)
> index.in.vector <- which(distance.vector == max.distance)
>    # determine which matrix element this is
> zero.based.index <- index.in.vector - 1L
> tumors.by.distance <- colnames(as.matrix(distance.matrix))
> count <- nrow(distance.matrix)
> i <- zero.based.index %% count  + 1L
> j <- zero.based.index %/% count + 1L
> tumor.1 <- rownames(distance.matrix)[i]
> tumor.2 <- colnames(distance.matrix)[j]
> printf("most dissimilar tumors: %s and %s", tumor.1, tumor.2)
```

```
[1] most dissimilar tumors: TCGA.02.0014 and TCGA.08.0385
```

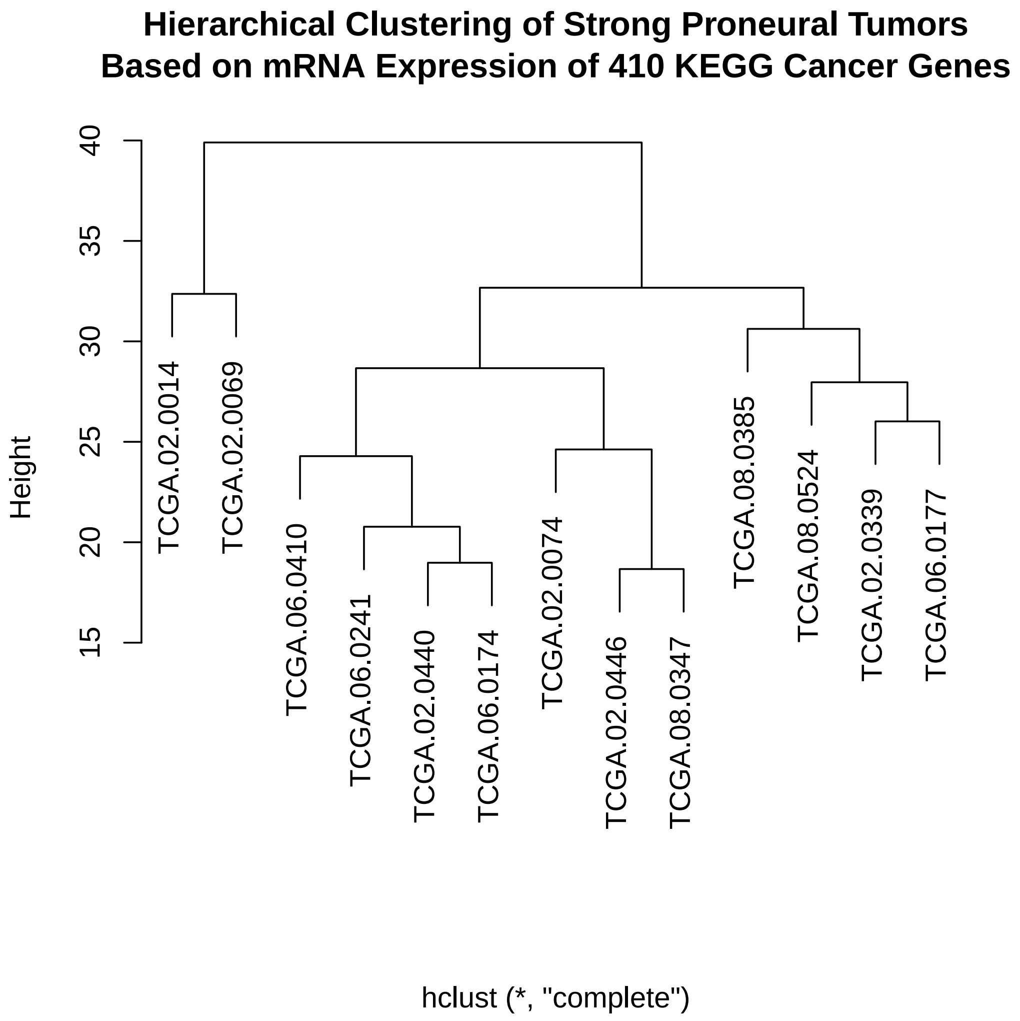


The second step is situate this pair of tumors within the distribution of all possible pairwise mRNA correlations within the strong proneural group, establishing that these two tumors, the most distant in a hierarchical clustering, are not outliers from the perspective of expression correlation.

```
> pairwise.correlations <- matrix (0, nrow=nrow(tbl), ncol=nrow(tbl),
+                                  dimnames=list(rownames(tbl), rownames(tbl)))
> tumor.names <- rownames(tbl)
> for (i in 1:nrow(tbl))
+     for (j in 1:nrow(tbl)) {
+        tumor.1 <- tumor.names[i]
+        tumor.2 <- tumor.names[j]
+        pair.correlation <- cor (tbl [i,], tbl [j,], use='pairwise.complete.obs')
+        pairwise.correlations[tumor.1, tumor.2] <- pair.correlation
+        } # for j
> diag(pairwise.correlations) <- NA
> title.1 <- "Density Plot of All Pairwise Tumor Corrlations"
> title.2 <- "(Marker Indicates Correlation of TCGA.02.0014 and TCGA.08.0385)"
```

```
> title <- paste(title.1, title.2, sep="\n")
> plot(density(pairwise.correlations, na.rm=TRUE), main=title, xlab="")
> text(pairwise.correlations[1,12], 1.65, "O", col="red")
```

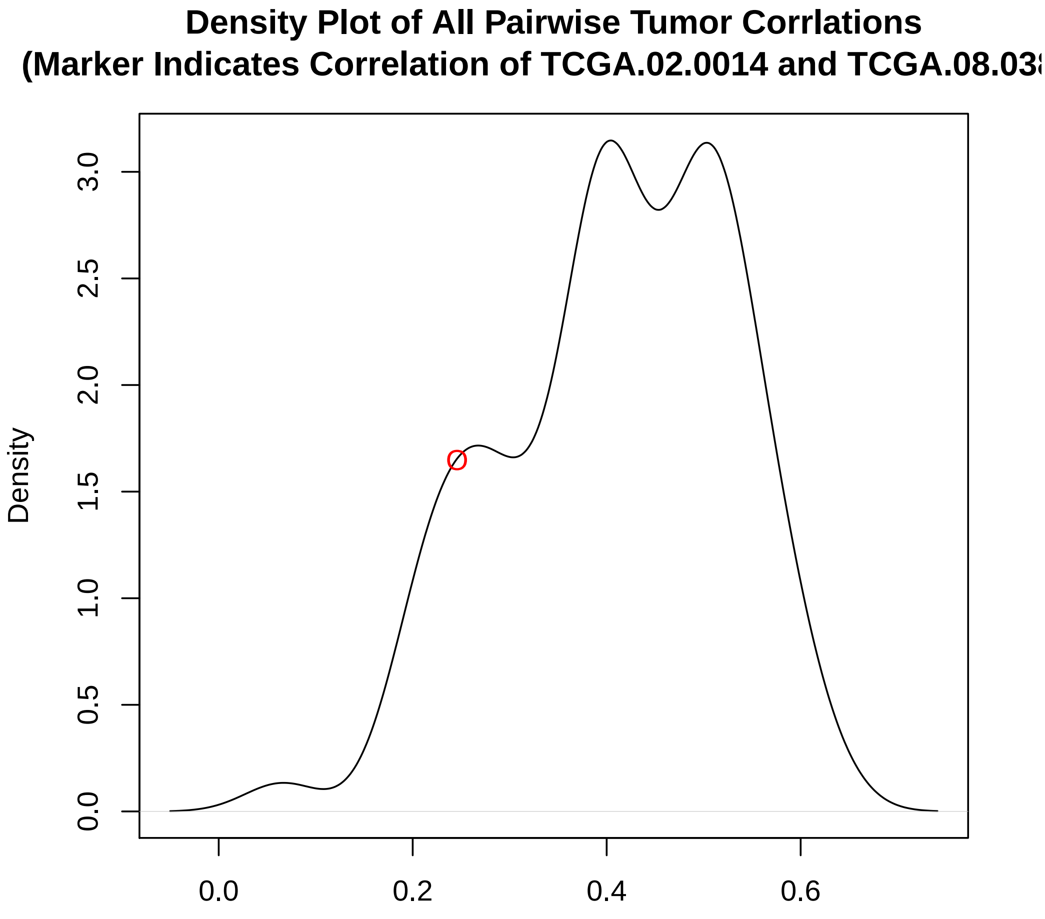

## 3 Display The Two Selected Strong Proneural Tumors

This section reproduces the two Cytoscape maps contained in [Shannon et al. (2013)].

```
> pdgfra.neighborhood.genes <- c("1282","1950","1956","2252","2254","2257","2261",
+                                "2263","2322","2335","25","25759","2885","3082",
+                                "3265","3479","3685","3845","387","4233","4893",
+                                "4914","5154","5155","5156","5294","5295","5336",
+                                "5337","5601","5605","5747","5879","5894","6464",
+                                "6654","673","801","8031","805","808","867","998")
> tbl.kegg = combineKeggPathways(c('05214', '04110', '05200'), organism='hsa')
> layout.file <- system.file("extdata", "layoutSmall.RData",
+                             package="ProneuralHeterogeneity")
> cw <- displayNetwork(tbl.kegg, layout.file, geneTypes,
+                      genes.of.interest=pdgfra.neighborhood.genes)
> setWindowSize(cw, 800, 600)   # laptop,  big screen: 1800, 1200
> setDefaultBackgroundColor(cw, '#F8F8F8')
> showGraphicsDetails(cw, TRUE)
> tumor <- "TCGA.02.0014"
> displayTumor(cw, tumor, tbl.mrna, tbl.cnv, tbl.mut)
> dimInactiveNodesAndEdges (cw, tumor, pdgfra.neighborhood.genes,
+                           tbl.mrna, tbl.cnv, tbl.mut,
+                           mrna.threshold=0.58, opacity=40)
> fitContent(cw)
> setZoom(cw, 0.9 * getZoom(cw))
```

Now display the other tumor:

```
> tumor <- "TCGA.08.0385"
> displayTumor(cw, tumor, tbl.mrna, tbl.cnv, tbl.mut)
```

```
[1] homo.deletion.gene count: 0
[1] hetero.deletion.gene count: 2
[1] gain.gene count: 33
[1] amplified.gene count: 10
[1] score: 397
[1] geneIDs: 397
An object of class "CytoscapeWindowClass"
Slot "title":
[1] "KEGG Cancer-Related"

Slot "window.id":
[1] "3"

Slot "graph":
A graphNEL graph with directed edges
Number of Nodes = 38
Number of Edges = 64
```

```
Slot "collectTimings":
[1] FALSE

Slot "uri":
[1] "http://localhost:9000"
```

```
> dimInactiveNodesAndEdges (cw, tumor, pdgfra.neighborhood.genes,
+                              tbl.mrna, tbl.cnv, tbl.mut,
+                              mrna.threshold=0.58, opacity=40)
```

```
 [1] "5154" "6654" "673"  "3845" "1282" "3685" "5747" "25"   "2322" "387"
[11] "2252"
```

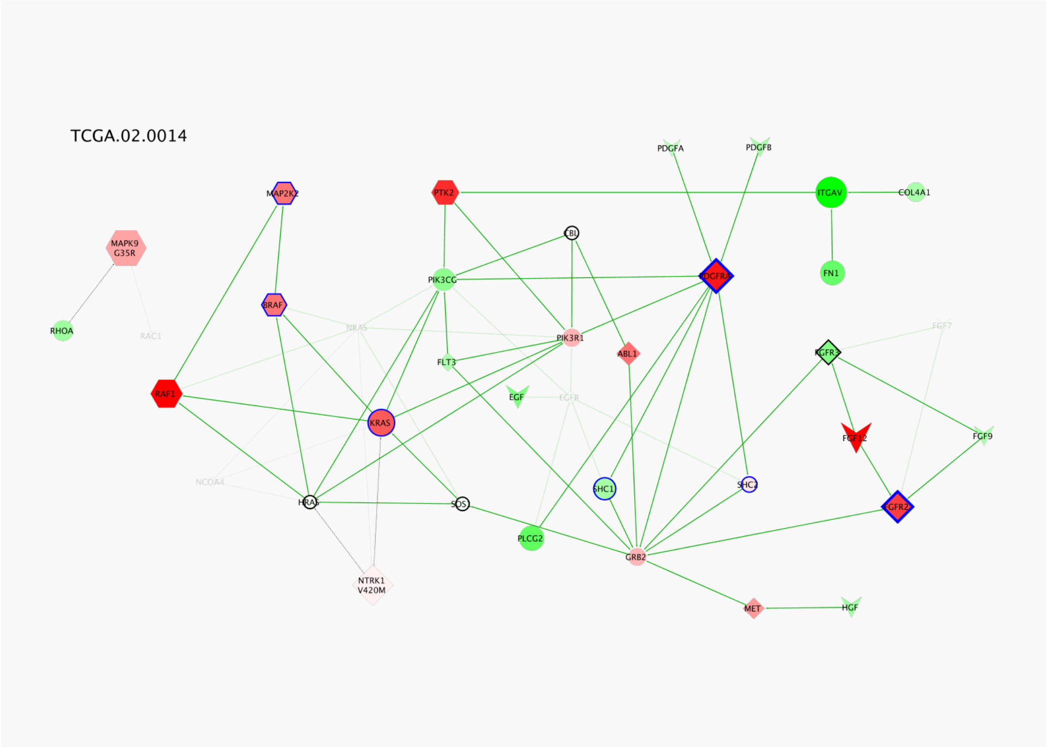

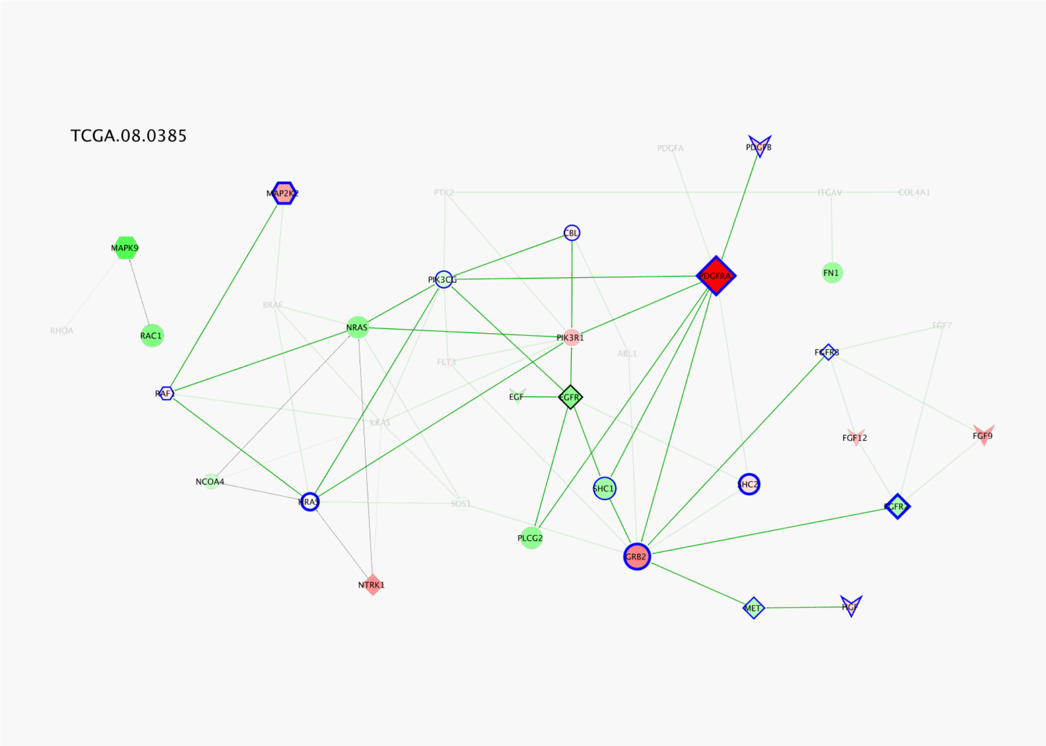


# 4 Create an RCytoscape Movie of all Strong Proneural Tumors

Here we build upon the (saveImage) method, calling it after each of the thirteen strong proneural tumors, and then (outside of R) assembling these *png* files into an animated gif to be viewed in a web browser.

```
for(tumor in proneural.tumors.strong){
    printf('now displaying tumor %s for movie', tumor)
    displayTumor(cw, tumor, tbl.mrna, tbl.cnv, tbl.mut)
    dimInactiveNodesAndEdges (cw, tumor, pdgfra.neighborhood.genes,
                              tbl.mrna, tbl.cnv, tbl.mut,
                              mrna.threshold=0.58, opacity=40)
    filename <- sprintf ("/Users/pshannon/s/bioc/trunk/RpacksTesting/ProneuralHeterogeneity/inst/extda
    saveImage(cw, filename, image.type="png", scale=3)
    }
```

The result, running at 500 millseconds per frame, may be seen here:

`http://rcytoscape.systemsbiology.net/versions/current/gallery/strongProneurals/strong50.gif`

```
fitContent(cw)
clearSelection(cw)
setZoom(cw, 0.8 * getZoom(cw))
for(tumor in proneural.tumors.strong){
```

```
printf('now displaying tumor %s for movie', tumor)
displayTumor(cw, tumor, tbl.mrna, tbl.cnv, tbl.mut)
dimInactiveNodesAndEdges (cw, tumor, nodes(cw@graph), #pdgfra.neighborhood.genes,
                          tbl.mrna, tbl.cnv, tbl.mut,
                          mrna.threshold=0.58, opacity=40)
filename <- sprintf ("/Users/pshannon/s/bioc/trunk/RpacksTesting/ProneuralHeterogeneity/inst/extda
saveImage(cw, filename, image.type="png", scale=3)
}
```

# References


S. Monti, P. Tamayo, J. Mesirov, and T. Golub. Consensus clustering: a resampling-based method for class discovery and visualization of gene expression microarray data. *Machine learning*, 52(1):91–118, 2003.

P. Shannon, M. Grimes, B. Kutlu, J. Bot, and D. Galas. RCytoscape: Tools for exploratory network analysis (in preparation). 2013.

J.W. Tukey. Exploratory data analysis. *Reading, MA*, 231, 1977.

R.G.W. Verhaak, K.A. Hoadley, E. Purdom, V. Wang, Y. Qi, M.D. Wilkerson, C.R. Miller, L. Ding, T. Golub, J.P. Mesirov, et al. An integrated genomic analysis identifies clinically relevant subtypes of glioblastoma characterized by abnormalities in PDGFRA, IDH1, EGFR and NF1. *Cancer cell*, 17(1):98, 2010.

J.D. Zhang and S. Wiemann. Kegggraph: a graph approach to kegg pathway in r and bioconductor. *Bioinformatics*, 25(11):1470–1471, 2009.
