# Supplementary material for: RCytoscape: tools for exploratory network analysis
Source: BMC Bioinformatics. 2013 Jul 9;14:217. doi: 10.1186/1471-2105-14-217 (PMC3751905; doi:10.1186/1471-2105-14-217)
Supplement: Additional file 6 — (An extended discussion of Receptor Tyrosine Kinase activation in heterogeneous Proneural GBM tumors (an abbreviated version appears in the body of the article). [file 1471-2105-14-217-S6.pdf]

Results and Discussion of Receptor Tyrosine Kinase Activation

To demonstrate the contribution which RCytoscape's molecular maps can make to biological understanding via exploratory data analysis, we here examine two tumors from one of the four categories of genomic abnormalities identified in glioblastoma multiforme (GBM) tumors in a TCGA study by Verhaak et al [[1]]. Though John Tukey recommends that EDA be the first step in data analysis, prior to model selection and model assessment, EDA is also useful at many recurring points throughout an analysis, as we will show. In this current case, we use network EDA to examine some results from Verhaak et al's consensus average linkage hierarchical clustering.

Our analysis is summarized below, and presented in full reproducible, statistical and narrative detail in the ProneuralHeterogeneity R package included in Supplemental Materials.

Though all cases of GBM are resistant to treatment, the Proneural subtype tumors are uniquely so[26]. We display molecular maps of the signaling network neighborhood of the growth factor receptor *PDGFRA*, whose high copy number and overexpression are the hallmark of Proneural tumors. *PDGFRA* and its neighbors are likely participants in the chronic proliferative signaling

---

[1] Verhaak, Roel GW, et al. "An integrated genomic analysis identifies clinically relevant subtypes of glioblastoma characterized by abnormalities in *PDGFRA*, *IDH1*, *EGFR* and *NF1*." *Cancer cell* 2010. 17.1: 98.

required for tumorigenesis [2]. In the first round of the TCGA study, 201 tumors were analyzed, 53 were classified as Proneural of which 13 exhibited the strongest signal (which we here identify as the "strong proneural" group): high copy number and mRNA expression for the *PDGFRA* gene. We chose two tumors for exploratory analysis from this set; (criteria and code for this selection is available in the supplemental materials). We suggest that the recalcitrance of Proneural tumors to treatment may be related to the heterogeneity of *PDGFRA*-related receptor tyrosine kinase (RTK) activity, and thus to robustly and diversely supported proliferative signaling, which is revealed by molecular maps of these two representative tumors.

RTKs play an important role in the regulation of cell growth; their dysregulation can contribute to cancer. In normal growth conditions, extracellular growth factors, whose production is highly regulated, bind to transmembrane RTKs, initiating signaling cascades which lead to orderly cell growth and division. The mechanism of activation for many RTKs is via the "receptor dimerization" [3] model, in which receptors move freely and laterally across the cell membrane, are bound by a growth factor ligand, and then "seek out" a second receptor, after which receptor dimerization takes place and signaling begins. Under this model, an excess of receptor, ligand, or both, can cause dysregulated activation. When cells exhibit these kinds of activation, we loosely describe them as autocrine loops -- "loosely" since a true autocrine loop occurs within a single cell, and the measurements used here are, as with most high throughput measurements, drawn from a large population of (mostly) homogeneous cells. Lastly, ligand-independent

---

[2] Hanahan, Douglas, and Robert A. Weinberg. "Hallmarks of cancer: the next generation." *Cell* 2011. 144.5: 646-674.

[3] Weinberg RA: *The Biology of Cancer*. New York: Garland, 2006, p 137.

activation of an RTK can be caused by non-synomymous mutations leading to constitutive dimerization of the receptors. Using mRNA expression as a proxy for signaling activity [4], active autocrine loops may be inferred in the maps below, based upon from genomic amplification and/or over-expression, at various levels, of an RTK ligand, an RTK receptor, or both.

The two maps shown below are a subset of a larger network assembled by merging three overlapping KEGG cancer-related signaling pathways: “Pathways in Cancer”, “Glioma” and “Cell Cycle”. The subset is organized around the *PDGFRA* receptor kinase and its network neighborhood, including members of the *PI3K*/*Akt*, and *MAPK*/*ERK* pathways. Nodes in the graphs simultaneously represent genes and the proteins for which they code. Gene copy number, mRNA expression, and amino acid substitutions are displayed on the nodes; signaling and regulatory relationships are portrayed by lines(edges) connecting the nodes.

(figure2.pdf goes here)

Instances of each of the three varieties of possible autocrine loops may be seen in these two RCytoscape maps. Different autocrine loops in the two tumors are strongly suggested by

---

[4] Graeber, Thomas G., and David Eisenberg. "Bioinformatic identification of potential autocrine signaling loops in cancers from gene expression profiles." *Nature genetics* 2001. 29.3: 295-300.

contrasting patterns of differential expression and high copy number involving three receptor-ligand pairs, each of which is implicated in Glioblastoma: *PDGFB-PDGFRA*[5], *HGF*/*MET*[6], *FGF12*/*FGFR2* [7,8].

Tumor TCGA.02.0014 shows neither over-expression nor gene duplication in any *PDGFRA* ligand, but does exhibit over-expression of both members of the *FGF12*/*FGR2* ligand/receptor pair (as well as gene duplication in the receptor). This activation may be a necessary complement to possible *PDGRFA* activation despite down-regulated ligand expression.

Tumor TCGA.08.0385 offers a sharp contrast. Here we see that the signature *PDGFRA* gene duplication and over-expression is seen also in its ligand *PDGFB*. We also see high expression and duplication in the *MET* ligand, *HGF*, a phenomenon not seen in the other tumor.

Other differences between the tumors stand out in these two maps: mutation or high expression is evident in TCGA.02.0014 for the MAP kinase pathway members, *KRAS*, *BRAF*, *MAP2K2* (*MEK2*), and *MAPK9* (*JNK2*), whereas in TCGA.08.0385, HRAS is amplified and only *MAP2K2* (*MEK2*) is amplified and over-expressed. These signaling pathway hotspots suggest

---

[5] Dai, Chengkai, et al. "PDGF autocrine stimulation dedifferentiates cultured astrocytes and induces oligodendrogliomas and oligoastrocytomas from neural progenitors and astrocytes in vivo." *Genes & development* 2001. 15.15: 1913-1925.

[6] Xie, Qian, et al. "Hepatocyte growth factor (HGF) autocrine activation predicts sensitivity to MET inhibition in glioblastoma." *Proceedings of the National Academy of Sciences* 2012. 109.2: 570-575.

[7] Loilome, Watcharin, et al. "Glioblastoma cell growth is suppressed by disruption of fibroblast growth factor pathway signaling." *Journal of Neuro-Oncology* 2009. 94.3: 359-366.

[8] Katoh, Masaru. "Cancer genomics and genetics of FGFR2 (Review)." *International journal of oncology* 2008. 33.2: 233.

other possible contributions to tumorigencity.   The heterogeneity of these two identically-classified Proneural tumors, as seen in these RCytocape network maps, is consistent with the hypothesis proposed by Stommel et al[21] “that multiple RTKs are coactivated in these [glioblastoma] tumors and that redundant inputs drive and maintain downstream signaling, thereby limiting the efficacy of therapies targeting single RTKs ... effective GBM therapy may require combined regimens targeting multiple RTKs.”
